# Frequentist Model Averaging in Structure Equation Model With Ordinal Data

**DOI:** 10.1007/s11336-021-09837-3

**Published:** 2022-01-29

**Authors:** Shaobo Jin

**Affiliations:** Department of Statistics, Uppsala University, Uppsala, Sweden

**Keywords:** mean squared error, confidence interval, goodness-of-fit test, model selection uncertainty, local asymptotic framework, pseudo maximum likelihood

## Abstract

In practice, it is common that a best fitting structural equation model (SEM) is selected from a set of candidate SEMs and inference is conducted conditional on the selected model. Such post-selection inference ignores the model selection uncertainty and yields too optimistic inference. Using the largest candidate model avoids model selection uncertainty but introduces a large variation. Jin and Ankargren (Psychometrika 84:84–104, 2019) proposed to use frequentist model averaging in SEM with continuous data as a compromise between model selection and the full model. They assumed that the true values of the parameters depend on $n^{-1/2}$ with *n* being the sample size, which is known as a local asymptotic framework. This paper shows that their results are not directly applicable to SEM with ordinal data. To address this issue, we prove consistency and asymptotic normality of the polychoric correlation estimators under the local asymptotic framework. Then, we propose a new frequentist model averaging estimator and a valid confidence interval that are suitable for ordinal data. Goodness-of-fit test statistics for the model averaging estimator are also derived.

**Supplementary Information:**

The online version contains supplementary material available at 10.1007/s11336-021-09837-3.

Structural equation models (SEMs) with ordinal data are widely used in social and behavioral sciences. As many other statistical models, a common practice is to choose an optimal model from a number of candidate models according to some criteria. A long-standing critique of model selection is that the post-selection inference is often conducted in such a way that model selection was never present. Because of the randomness in the data, the model selection step is also stochastic and ignoring such uncertainty yields too optimal inference. The reader is directed to Preacher and Merkle (2012) and Lubke et al. (2017) for discussions on the consequences of ignoring model selection uncertainty within SEM.

A well-known remedy to acknowledge the contribution of all candidate models is Bayesian model averaging. Instead of purely relying on the optimal model, Bayesian model averaging combines different candidate models together by a weighted average (Hoeting et al., 1999; Madigan & Raftery, 1994). In the past decades, the interest on frequentist model averaging (FMA) has grown exponentially in the statistics literature. The reader is directed to Fletcher (2018) for a long list of FMA related references.

The main purpose of the paper is to generalize the FMA principle to SEMs with ordinal data. Jin and Ankargren (2019) have applied the FMA technique to SEMs with continuous data, using the likelihood-based FMA machinery developed by Hjort and Claeskens (2003a). Similar to FMA in the other context, they showed that FMA tends to produce a mean squared error (MSE) that is lower than that of the full model if the population parameter value is small and is lower than that of model selection if the population value is moderate. Hence, the FMA estimator is a robust compromise between model selection and the full model in the SEM context. Since ordinal data are often encountered in practice, it is of interest to extend the FMA technique for SEM with continuous data to SEM with ordinal data. As we shall see in the later sections, some results in Jin and Ankargren (2019) need to be revised for ordinal data.

The rest of the paper is organized as follows. First, the results in Jin and Ankargren (2019) are briefly reviewed. Second, the necessary modifications for the FMA estimator in ordinal SEM are presented. Third, a simulation study is conducted to investigate the small sample properties of the FMA technique. Fourth, FMA is applied to an empirical example as an illustration. A discussion ends the paper.

## Background

Consider the SEM

$$\begin{aligned} \varvec{x}^{*}= & {} \varvec{\Lambda }_{x}\varvec{\xi }+\varvec{\delta }_{x},\\ \varvec{y}^{*}= & {} \varvec{\Lambda }_{y}\varvec{\eta }+\varvec{\delta }_{y}, \\ \varvec{\eta }= & {} \varvec{B}\varvec{\eta }+\varvec{\Gamma }\varvec{\xi }+\varvec{\varepsilon }, \end{aligned}$$

where $\varvec{x}^{*}$ ($p_x \times 1$) and $\varvec{y}^{*}$ ($p_y \times 1$) are the vectors of continuous indicators, $\varvec{\xi }$ and $\varvec{\eta }$ are the latent variables, $\varvec{\delta }_{x}$, $\varvec{\delta }_{y}$, and $\varvec{\varepsilon }$ are the error terms. Here, $\varvec{\Lambda }_{x}$ and $\varvec{\Lambda }_{y}$ are the loading matrices, $\varvec{B}$ contains the linear effects among $\varvec{\eta }$, and $\varvec{\Gamma }$ contains linear effects of $\varvec{\xi }$ on $\varvec{\eta }$. The joint distribution of $\varvec{x}^{*}$ and $\varvec{y}^{*}$ is assumed to be multivariate normal. Throughout the paper, $\varvec{\Sigma }$ is used to denote the model-implied covariance of the SEM, $\varvec{\sigma }$ the vector of unique entries in $\varvec{\Sigma }$, and $\varvec{\beta }$ the vector of all free parameters needed to determine $\varvec{\Sigma }$ (i.e., free parameters in $\varvec{\Lambda }_{x}$, $\varvec{\Lambda }_{y}$, $\varvec{B}$, $\varvec{\Gamma }$, $\text {var}(\varvec{\xi })$, $\text {var}(\varvec{\delta }_{x})$, $\text {var}(\varvec{\delta }_{y})$, $\text {cov}(\varvec{\delta }_{x},\varvec{\delta }_{y}^T)$, and $\text {var}(\varvec{\varepsilon })$).

### Brief Review of Jin and Ankargren (2019)

Jin and Ankargren (2019) investigated FMA in SEM where $\varvec{x}^{*}$ and $\varvec{y}^{*}$ are observed. In order to clarify the modifications needed for ordinal SEM, their results are briefly reviewed in this subsection. Suppose that there exist a number of candidate SEMs, indexed by *s*, in which a full model that nests all other models and a narrow model that is nested in all other models are well-defined. The vector $\varvec{\beta }$ is partitioned into $\varvec{\beta }^{T}=\left( \varvec{\theta }^{T},\varvec{\gamma }^{T}\right)$, where $\varvec{\theta }$ is contained in all candidate models and $\varvec{\gamma }=\varvec{\gamma }_0$ is known in the narrow model. For candidate model *s*, the elements in $\varvec{\gamma }$ to be estimated are $\varvec{\gamma }_{s}=\varvec{\pi }_{s}\varvec{\gamma }$, where $\varvec{\pi }_{s}$ is a selection matrix. The candidate models are fitted by maximum likelihood, i.e., minimizing $F_{ML}(\varvec{\beta }) = n\log \left| \varvec{\Sigma }\right| + n \text {tr}\left\{ \varvec{S}\varvec{\Sigma }^{-1}\right\} - n\log \left| \varvec{S}\right| - n\left( p_x + p_y \right)$, where *n* is the sample size, $\varvec{S}$ is the sample covariance matrix, and $\text {tr} \left\{ \right\}$ is the matrix trace of the enclosed matrix. Suppose that the parameter vector of interest is $\varvec{\mu }=\varvec{\mu }\left( \varvec{\theta },\varvec{\gamma }\right)$, which is continuously differentiable in $\varvec{\theta }$ and $\varvec{\gamma }$. The FMA estimator of $\varvec{\mu }$ is $\bar{\varvec{\mu }} \left( \varvec{c} \right) = \sum _{s}c_{s}\hat{\varvec{\mu }}_{s}$, where $\hat{\varvec{\mu }}_{s}$ is the estimator of $\varvec{\mu }$ in the candidate model *s* and the model weight vector $\varvec{c} = \left\{ c_{s}\right\}$ lies in the unit simplex $\{ c_{s}:\sum _{s}c_{s}=1,\,0\le c_{s}\le 1 \}$.

Jin and Ankargren (2019) assumed that $\varvec{\beta }_{true} = \left( \varvec{\theta }_{0}^T,\varvec{\gamma }_{0}^T+\varvec{\delta }^T/\sqrt{n}\right) ^T$ is the true value of $\varvec{\beta }$, where $\varvec{\theta }_{0}$ is the true value of $\varvec{\theta }$, $\varvec{\gamma }_{0}+\varvec{\delta }/\sqrt{n}$ is the true value of $\varvec{\gamma }$, and $\varvec{\delta }$ is the local parameter. Hence, the true values of $\varvec{\sigma }$ and $\varvec{\mu }$ are $\varvec{\sigma }_{true} = \varvec{\sigma }\left( \varvec{\beta }_{true}\right)$ and $\varvec{\mu }_{true} = \varvec{\mu }\left( \varvec{\beta }_{true} \right)$, respectively. The framework that the true value is drifted in a $n^{-1/2}$ neighborhood is known as the local asymptotic framework. In contrast, the standard asymptotic framework refers to the case where all true values are free of *n*. The local asymptotic framework is a popular choice to study FMA. The reader is directed to Hjort and Claeskens (2003b) for the reasons of using the local asymptotic framework.

Suppose that $n^{-1} \partial ^{2}F_{ML}\left( \varvec{\beta }_{0}\right) / \partial \varvec{\beta }\partial \varvec{\beta }^{T}$ converges in probability to $2\varvec{J}_{full}$, where $\varvec{\beta }_{0} = \left( \varvec{\theta }_{0}^T,\varvec{\gamma }_{0}^T\right) ^T$ and $\varvec{J}_{full}$ can be partitioned to

$$\begin{aligned} \varvec{J}_{full} = \begin{pmatrix}\varvec{J}_{\theta \theta } &{} \varvec{J}_{\theta \gamma }\\ \varvec{J}_{\theta \gamma }^{T} &{} \varvec{J}_{\gamma \gamma } \end{pmatrix}. \end{aligned}$$

Let $\varvec{M}$ and $\varvec{N}$ be the random variables such that

$$\begin{aligned} -\frac{1}{2\sqrt{n}}\frac{\partial F_{ML} \left( \varvec{\beta }_{0}\right) }{\partial \varvec{\beta }}-\begin{pmatrix} \varvec{J}_{\theta \gamma }\\ \varvec{J}_{\gamma \gamma } \end{pmatrix} \varvec{\delta }&\overset{d}{\rightarrow } \begin{pmatrix}\varvec{M}\\ \varvec{N} \end{pmatrix}. \end{aligned}$$

For fixed weights, Jin and Ankargren (2019) derived that

1 $$\begin{aligned} \sqrt{n}\left( \bar{\varvec{\mu }}-\varvec{\mu }_{true}\right) =&\frac{\partial \varvec{\mu }_{0}}{\partial \varvec{\theta }^{T}}\varvec{J}_{\theta \theta }^{-1}\varvec{M}+\varvec{W}\left\{ \varvec{\delta }-\left( \sum _{s}c_{s}\varvec{K}^{(s)}\right) \varvec{K}^{-1}\varvec{D}\right\} +O_{\text {P}}\left( n^{-1/2}\right) , \end{aligned}$$

where $\varvec{\mu }_{0} = \varvec{\mu }\left( \varvec{\theta }_{0},\varvec{\gamma }_{0}\right)$, $\varvec{W}= \frac{\partial \varvec{\mu }_{0}}{\partial \varvec{\theta }^{T}}\varvec{J}_{\theta \theta }^{-1}\varvec{J}_{\theta \gamma }-\frac{\partial \varvec{\mu }_{0}}{\partial \varvec{\gamma }^{T}}$, $\varvec{K}^{-1}=\varvec{J}_{\gamma \gamma }-\varvec{J}_{\theta \gamma }^{T}\varvec{J}_{\theta \theta }^{-1}\varvec{J}_{\theta \gamma }$, $\varvec{K}_{s}=\left( \varvec{\pi }_{s}\varvec{K}^{-1}\varvec{\pi }_{s}^{T}\right) ^{-1}$, $\varvec{K}^{(s)} = \varvec{\pi }_{s}^{T}\varvec{K}_{s}\varvec{\pi }_{s}$, and $\varvec{D}= \varvec{\delta }-\varvec{K}\varvec{J}_{\theta \gamma }^{T}\varvec{J}_{\theta \theta }^{-1}\varvec{M}+\varvec{K}\varvec{N}$^1^. To estimate $\varvec{c}$, the limit of $nE\left( \bar{\varvec{\mu }}-\varvec{\mu }_{true}\right) ^{T}\left( \bar{\varvec{\mu }}-\varvec{\mu }_{true}\right)$ is minimized, which is equivalent to minimizing

2 $$\begin{aligned} Q\left( \varvec{c}\right) =&\sum _{s}c_{s}tr\left\{ \varvec{\Delta }_1 \varvec{K}^{(s)} \varvec{W}^{T}\right\} +\frac{1}{2}\sum _{s}\sum _{t}c_{s}c_{t} \text {tr} \left\{ \varvec{W}\varvec{K}^{(s)}\varvec{\Delta }_2 \varvec{K}^{(t)} \varvec{W}^{T}\right\} , \end{aligned}$$

subject to the unit simplex, where $\varvec{\Delta }_1 = -\varvec{W}\varvec{\delta }\varvec{\delta }^{T}\varvec{K}^{-1}$, and $\varvec{\Delta }_2 = \varvec{K}^{-1} + \varvec{K}^{-1}\varvec{\delta }\varvec{\delta }^{T}\varvec{K}^{-1}$.

In the spirit of Hjort and Claeskens (2003a) and Liu (2015), Jin and Ankargren (2019) also proposed a confidence interval for $\mu _{i}$, the *i*th entry in $\varvec{\mu }$, which is given by

3 $$\begin{aligned} \left[ {\bar{\mu }}_{i}-\frac{{\hat{u}}_{i}}{\sqrt{n}}-z_{1-\alpha /2}\frac{{\hat{\kappa }}_{i}}{\sqrt{n}},\qquad {\bar{\mu }}_{i}-\frac{{\hat{u}}_{i}}{\sqrt{n}}+z_{1-\alpha /2}\frac{{\hat{\kappa }}_{i}}{\sqrt{n}}\right] , \end{aligned}$$

where ${\hat{u}}_{i}$ is the *i*th entry of the vector $\hat{\varvec{W}}\left( \hat{\varvec{\delta }} - \tilde{\varvec{\delta }} \right)$ with $\tilde{\varvec{\delta }} = \sum _{s}{\hat{c}}_{s} \hat{\varvec{K}}^{(s)} \hat{\varvec{K}}^{-1}\hat{\varvec{\delta }}$, $z_{1-\alpha /2}$ is the $1-\alpha /2$ quantile of the standard normal distribution, and $\kappa _{i}$ is (*i*, *i*)th entry of the covariance matrix of $\frac{\partial \varvec{\mu }_{0}}{\partial \varvec{\theta }^{T}}\varvec{J}_{\theta \theta }^{-1}\varvec{M}-\varvec{W}\varvec{D}$ with ${\hat{\kappa }}_i$ being its estimator.

### Ordinal Data Model

In the ordinal SEM, the ordinal counterparts $\varvec{x}$ and $\varvec{y}$ are observed, which are obtained by discretizing $\varvec{x}^{*}$ and $\varvec{y}^{*}$. In the ordinal data model, the diagonal entries in $\varvec{\Sigma }$ are assumed to be 1. Accordingly, $\varvec{\Sigma }$ is a polychoric correlation matrix. In practice, a multi-step procedure is commonly used to fit an ordinal SEM. First, the polychoric correlation matrix and its asymptotic covariance matrix are estimated. Second, the least squares fit function $F_{LS}\left( \varvec{\beta }\right) = n\left( \hat{\varvec{\rho }}-\varvec{\sigma }\left( \varvec{\beta }\right) \right) ^{T} \hat{\varvec{V}} \left( \hat{\varvec{\rho }}-\varvec{\sigma }\left( \varvec{\beta }\right) \right)$ is minimized, where $\varvec{\rho }$ is the vector of unique polychoric correlation coefficients and $\varvec{V}$ is some weight matrix.

In the current study, we will extend Jin and Ankargren (2019) to SEM with ordinal data. Since both $F_{ML}$ and $F_{LS}$ can be viewed as distance functions between $\varvec{\rho }$ and $\varvec{\sigma }$, many results from Jin and Ankargren (2019) still hold. However, as we shall explain in the next section, some modifications are also needed, due to the choice of $\varvec{V}$.

## Frequentist Model Averaging Estimator

### Polychoric Correlation Estimation

Throughout the paper, we estimate the polychoric correlation coefficients using the two-step procedure of Olsson (1979). First, the thresholds are estimated from the univariate standard normal distribution. Second, the correlation coefficient is estimated conditional on the estimated thresholds. Estimation of the polychoric correlation matrix and its asymptotic covariance matrix has been extensively studied (e.g., Jin & Yang-Wallentin, 2017; Jöreskog, 1994; Monroe, 2017; Muthén, 1984) within the standard asymptotic framework. For example, Jöreskog (1994) showed that

4 $$\begin{aligned} \sqrt{n}\left( \hat{\varvec{\rho }} - \varvec{\sigma }_0\right) \overset{d}{\rightarrow } N\left( \varvec{0},\varvec{\Upsilon }\right) , \end{aligned}$$

where $\varvec{\sigma }_0=\varvec{\sigma }\left( \varvec{\beta }_0 \right)$ and $\varvec{\Upsilon }$ is the asymptotic covariance matrix.

We still assume that the thresholds are not locally drifted. However, the true values of the polychoric correlation coefficients depend on *n*. To the best of our knowledge, none of the above mentioned studies on polychoric correlations are conducted under the local asymptotic framework. Further, the polychoric correlation estimator from the two-step procedure is a pseudo-maximum likelihood estimator (Gong & Samaniego, 1981), making the results in Hjort and Claeskens (2003a) not directly applicable. For these reasons, we establish consistency and asymptotic normality of the polychoric correlation estimators in this subsection. For ease of presentation, all regularity conditions and mathematical proofs are placed in the online appendix.

#### Theorem 1

Under the regularity conditions stated in the online appendix, $\hat{\varvec{\rho }} \overset{p}{\rightarrow } \varvec{\sigma }_{0}$.

Theorem 1 shows that $\hat{\varvec{\rho }}$ remains a consistent estimator of $\varvec{\sigma }_{0}$ under the local asymptotic framework. The following theorem shows asymptotic normality.

#### Theorem 2

Under the regularity conditions stated in the online appendix, $\sqrt{n}\left( \hat{\varvec{\rho }}-\varvec{\sigma }_{true}\right) \overset{d}{\rightarrow }N\left( \varvec{0},\varvec{\Upsilon }\right)$, where $\varvec{\Upsilon }$ is the same as the covariance matrix in (4).

Theorem 2 shows that the estimator of the asymptotic covariance matrix under the standard asymptotic framework is also valid under the local asymptotic framework. The implication is that the estimated asymptotic covariance matrix can simply be extracted from the standard SEM packages. Theorem 2 also shows that the mean of the limiting distribution of $\sqrt{n}\left( \hat{\varvec{\rho }}-\varvec{\sigma }_{0}\right)$ is nonzero under the local asymptotic framework. In contrast, the mean of the limiting distribution of $\sqrt{n}\left( \hat{\varvec{\rho }}-\varvec{\sigma }_{0}\right)$ is zero under the standard asymptotic framework.

### Frequentist Model Averaging: From Continuous Data to Ordinal Data

Since both $F_{LS}$ and $F_{ML}$ can be viewed as distance functions between $\varvec{\rho }$ and $\varvec{\sigma }$, the expansion (1) still holds if $F_{ML}$ is replaced by $F_{LS}$ when obtaining $\varvec{J}_{full}$, $\varvec{M}$, and $\varvec{N}$. However, Jin and Ankargren (2019) derived the weight estimation criterion (2) and the confidence interval (3) under the assumption that $\varvec{M}$ and $\varvec{D}$ are independent, which holds for observed multivariate normal data. Regarding ordinal SEM, it is shown in the appendix that the joint distribution of $\varvec{M}$ and $\varvec{N}$ is multivariate normal with mean $\varvec{0}$ and covariance matrix

5 $$\begin{aligned} \varvec{H} \overset{\text {def}}{=} \left( \frac{\partial \varvec{\sigma }_{0}}{\partial \varvec{\beta }^{T}}\right) ^{T}\varvec{V}\varvec{\Upsilon }\varvec{V}\frac{\partial \varvec{\sigma }_{0}}{\partial \varvec{\beta }^{T}}. \end{aligned}$$

Consequently,

6 $$\begin{aligned} \text {cov}\left( \varvec{M},\varvec{D}^{T}\right) =&\left( \frac{\partial \varvec{\sigma }_{0}}{\partial \varvec{\theta }^{T}}\right) ^{T}\varvec{V}\varvec{\Upsilon }\varvec{V}\left( \frac{\partial \varvec{\sigma }_{0}}{\partial \varvec{\gamma }^{T}}\right) \varvec{K}-\left( \frac{\partial \varvec{\sigma }_{0}}{\partial \varvec{\theta }^{T}}\right) ^{T}\varvec{V}\varvec{\Upsilon }\varvec{V}\left( \frac{\partial \varvec{\sigma }_{0}}{\partial \varvec{\theta }^{T}}\right) \varvec{J}_{\theta \theta }^{-1}\varvec{J}_{\theta \gamma }\varvec{K}, \end{aligned}$$

which depends on the choice of $\varvec{V}$. Commonly used $\varvec{V}$ includes $\varvec{V}=\varvec{I}$ in unweighted least squares (ULS; Muthén, 1978), the inverse of diagonal elements of $\varvec{\Upsilon }$ in diagonally weighted least squares (DWLS; Muthén et al., 1997), and $\varvec{V}=\varvec{\Upsilon }^{-1}$ in weighted least squares (WLS; Browne, 1984). If WLS is used, then $\text {cov}\left( \varvec{M},\varvec{D}^{T}\right) = \varvec{0}$ and the results in Jin and Ankargren (2019) remain applicable. However, if ULS or DWLS is used, $\varvec{M}$ and $\varvec{D}$ are not necessarily independent. Consequently, modifications are needed.

### Weight Estimation

It is shown in the online appendix that, in the context of ordinal SEM, minimizing the limit of $nE\left( \bar{\varvec{\mu }}-\varvec{\mu }_{true}\right) ^{T}\left( \bar{\varvec{\mu }}-\varvec{\mu }_{true}\right)$ is equivalent to minimizing $Q\left( \varvec{c}\right)$ given in (2), but with modified $\varvec{\Delta }_1$ and $\varvec{\Delta }_2$ as

$$\begin{aligned} \varvec{\Delta }_1 =&\frac{\partial \varvec{\mu }_{0}}{\partial \varvec{\theta }^{T}}\varvec{J}_{\theta \theta }^{-1}\left( \text {var}\left( \varvec{M}\right) \varvec{J}_{\theta \theta }^{-1}\varvec{J}_{\theta \gamma }-\text {cov}\left( \varvec{M},\varvec{N}^{T}\right) \right) -\varvec{W}\varvec{\delta }\varvec{\delta }^{T}\varvec{K}^{-1} , \\ \varvec{\Delta }_2 =&\varvec{J}_{\theta \gamma }^{T}\varvec{J}_{\theta \theta }^{-1}\text {var}\left( \varvec{M}\right) \varvec{J}_{\theta \theta }^{-1}\varvec{J}_{\theta \gamma }+\text {var}\left( \varvec{N}\right) -2\varvec{J}_{\theta \gamma }^{T}\varvec{J}_{\theta \theta }^{-1}\text {cov}\left( \varvec{M},\varvec{N}^{T}\right) +\varvec{K}^{-1}\varvec{\delta }\varvec{\delta }^{T}\varvec{K}^{-1}, \end{aligned}$$

where the covariance matrix of $\varvec{M}$ and $\varvec{N}$ is shown in (5).

In practice, the unknown population values (e.g., $\varvec{\Delta }_{1}$, $\varvec{\Delta }_{2}$, $\varvec{K}^{(s)}$, and $\varvec{W}$) are replaced by their estimators from the candidate models, yielding the estimator ${\hat{Q}}\left( \varvec{c}\right)$ of $Q\left( \varvec{c}\right)$. Then, $\hat{\varvec{c}}$, the estimator of $\varvec{c}$, is obtained by minimizing ${\hat{Q}}\left( \varvec{c}\right)$. Similar to Jin and Ankargren (2019), the unknown population values can be consistently estimated from the full model, except $\varvec{\delta }$. Hjort and Claeskens (2003a) and Liu (2015) showed that $\varvec{\delta }$ can only be asymptotically unbiasedly estimated and suggested to estimate it from the full model as $\hat{\varvec{\delta }}=\sqrt{n}\left( \hat{\varvec{\gamma }}_{full}-\varvec{\gamma }_{0}\right)$, where $\hat{\varvec{\gamma }}_{full}$ is the estimator of $\varvec{\gamma }$ from the full model. It is an unbiased estimator of $\varvec{\delta }$ but a biased estimator of $\varvec{\delta }\varvec{\delta }^{T}$. Jin and Ankargren (2019) showed that, for SEM with continuous data, $\hat{\varvec{\delta }}$ remains an unbiased estimator of $\varvec{\delta }$ and $\hat{\varvec{\delta }}\hat{\varvec{\delta }}^{T} -\varvec{K}$ is an unbiased estimator of $\varvec{\delta }\varvec{\delta }^{T}$. It is shown in the online appendix that, for SEM with ordinal data, $\hat{\varvec{\delta }}$ is still an unbiased estimator of $\varvec{\delta }$, but an unbiased estimator of $\varvec{\delta }\varvec{\delta }^{T}$ becomes $\hat{\varvec{\delta }}\hat{\varvec{\delta }}^{T} - \hat{\varvec{G}} \hat{\varvec{H}} \hat{\varvec{G}} ^T$, where $$\varvec{G} = \begin{pmatrix}-\varvec{K}\varvec{J}_{\theta \gamma }^{T}\varvec{J}_{\theta \theta }^{-1}&\varvec{K}\end{pmatrix}$$.

### Model Averaging Confidence Interval

In the spirit of Hjort and Claeskens (2003a) and Liu (2015), we conjecture that there is joint convergence in distribution of $\hat{\varvec{c}}$ and all $\hat{\varvec{\mu }}_s$ such that

7 $$\begin{aligned} \sqrt{n}\left( \bar{\varvec{\mu }}\left( \hat{\varvec{c}}\right) -\varvec{\mu }_{true}\right) -\hat{\varvec{W}}\left( \hat{\varvec{\delta }}-\tilde{\varvec{\delta }}\right) \overset{d}{\rightarrow } \frac{\partial \varvec{\mu }_{0}}{\partial \varvec{\theta }^{T}}\varvec{J}_{\theta \theta }^{-1}\varvec{M}+\varvec{W}\left( \varvec{\delta }-\varvec{D}\right) . \end{aligned}$$

The reader is directed to the online appendix for a heuristic proof of the joint convergence. From (7), the FMA confidence interval for $\mu _i$ is still of the form (3) for SEM with ordinal data. In Jin and Ankargren (2019),

$$\begin{aligned} \text {cov}\left( \frac{\partial \varvec{\mu }_{0}}{\partial \varvec{\theta }^{T}}\varvec{J}_{\theta \theta }^{-1}\varvec{M}-\varvec{W}\varvec{D} \right) = \frac{\partial \varvec{\mu }_{0}}{\partial \varvec{\theta }^{T}}\varvec{J}_{\theta \theta }^{-1}\left( \frac{\partial \varvec{\mu }_{0}}{\partial \varvec{\theta }^{T}}\right) ^{T}+\varvec{W}\varvec{K} \varvec{W}^{T}. \end{aligned}$$

In an ordinal SEM, the covariance matrix of $\frac{\partial \varvec{\mu }_{0}}{\partial \varvec{\theta }^{T}}\varvec{J}_{\theta \theta }^{-1}\varvec{M}-\varvec{W}\varvec{D}$ should be computed using (6), due to nonzero $\text {cov}(\varvec{M},\varvec{D}^{T})$. Similar to weight estimation, the confidence interval in Jin and Ankargren (2019) remains applicable if WLS is used.

If (7) holds, the interval (3) that accounts for $\text {cov}(\varvec{M},\varvec{D}^{T})$ attains the nominal level asymptotically. Another valid confidence interval is the one from the full model, given by $\left[ {\hat{\mu }}_{i,full} - z_{1-\alpha /2} {\hat{\kappa }}_{i} / \sqrt{n}, \, {\hat{\mu }}_{i,full} + z_{1-\alpha /2} {\hat{\kappa }}_{i} / \sqrt{n}\right]$, where ${\hat{\mu }}_{i,full}$ is the estimator of $\mu _i$ from the full model. Various studies (e.g., Ankargren & Jin, 2018; Kabaila & Leeb, 2006; Wang & Zhou, 2013) have shown that the FMA confidence intervals of the form (3) can be asymptotically equivalent to the confidence interval from the full model. In the likelihood context, Wang and Zhou (2013) proved that

8 $$\begin{aligned} \bar{\varvec{\mu }}\left( \hat{\varvec{c}}\right) - \varvec{W}\left( \hat{\varvec{\delta }}-\tilde{\varvec{\delta }}\right) /\sqrt{n} =&\hat{\varvec{\mu }}_{full}+o_\text {P}\left( n^{-1/2}\right) , \end{aligned}$$

and showed that the equivalence holds for all FMA confidence intervals suggested by Hjort and Claeskens (2003a). Since least squares are used in SEM with ordinal data, their results cannot be directly applied here. Nevertheless, it is shown in the online appendix that (8) still holds in ordinal SEM. Hence, the FMA confidence interval (3) remains asymptotically equivalent to the full model interval. The implication is that the small sample realizations of (3) may be different from the full model interval, but they will be similar to each other when the sample size is large.

### Goodness-of-fit Test

Consider the case where $\varvec{\mu }=\varvec{\beta }$, i.e., we want all parameters (expect the thresholds) to be accurately estimated. In practice, it is often of interest to test the overall fit of a hypothesized model. The full model goodness-of-fit test can be interpreted as testing whether the full model decomposition of the population covariance matrix evaluated at the full model estimator fits the data well. Since FMA aims to combine different models, the population covariance matrix is generally decomposed according to the full model, but evaluated at the FMA estimator. Hence, it is important to test whether the full model evaluated at the FMA estimators fits the data well (Jin & Ankargren, 2019). For this reason, a goodness-of-fit test for model averaged SEM with ordinal data is proposed here.

A residual-based test statistic is

$$\begin{aligned} T_{FMA}=\left( \sqrt{n}\left( \hat{\varvec{\rho }}-\bar{\varvec{\sigma }}\right) +\frac{\partial \varvec{\sigma } \left( \hat{\varvec{\beta }}_{full} \right) }{\partial \varvec{\beta }^{T}} \hat{\varvec{W}} \left( \hat{\varvec{\delta }}-\tilde{\varvec{\delta }}\right) \right) ^{T} \hat{\varvec{V}} \left( \sqrt{n}\left( \hat{\varvec{\rho }}-\bar{\varvec{\sigma }}\right) +\frac{\partial \varvec{\sigma } \left( \hat{\varvec{\beta }}_{full} \right) }{\partial \varvec{\beta }^{T}} \hat{\varvec{W}} \left( \hat{\varvec{\delta }}-\tilde{\varvec{\delta }}\right) \right) , \end{aligned}$$

where $\bar{\varvec{\sigma }}=\varvec{\sigma }\left( \bar{\varvec{\mu }}\left( \hat{\varvec{c}}\right) \right)$. The asymptotic property of $T_{FMA}$ is shown in following theorem.

#### Theorem 3

Let $\varvec{\mu }=\varvec{\beta }$. Suppose that the regularity conditions in the online appendix hold. Then, $T_{FMA}=T_{full}+o_{\text {P}}\left( 1\right)$, where $T_{full}= n\left( \hat{\varvec{\rho }}-\hat{\varvec{\sigma }}_{full}\right) ^{T} \hat{\varvec{V}} \left( \hat{\varvec{\rho }}-\hat{\varvec{\sigma }}_{full}\right)$ is the test statistic for the full model.

The assumption that $\varvec{\mu }=\varvec{\beta }$ plays an important role in the proof of the theorem. It is certainly the case that some applications may be only interested in a subset of $\varvec{\beta }$. In such a case, Theorem 3 is not guaranteed to be applicable. A different test statistic is proposed by Jin and Ankargren (2019) for SEM with continuous data. Both test statistics adjust the fit function ($F_{ML}$ or $F_{LS}$), but with different adjustments. The adjustment $\frac{\partial \varvec{\sigma }_{full}}{\partial \varvec{\beta }^{T}}\varvec{W}\left( \hat{\varvec{\delta }}-\tilde{\varvec{\delta }} \right)$ is used in $T_{FMA}$, since $\varvec{W}\left( \hat{\varvec{\delta }}-\tilde{\varvec{\delta }} \right)$ is also the adjustment term in the FMA confidence interval (3).

Since $T_{FMA}$ is asymptotically equivalent to $T_{full}$, we can define the Satorra and Bentler (1994) mean-scaled statistic and mean-variance adjusted statistic as

$$\begin{aligned} T_{FMA-SB-m} = \frac{r}{\text {tr} \left( \hat{\varvec{\Xi }}_{FMA}\right) }T_{FMA} \text { and } T_{FMA-SB-mv} = \frac{ \text {tr} \left( \hat{\varvec{\Xi }}_{FMA}\right) }{ \text {tr} \left( \hat{\varvec{\Xi }}_{FMA}^{2}\right) }T_{FMA}, \end{aligned}$$

respectively, where *r* is the difference between the number of unique polychoric correlation coefficients and the number of parameters and

$$\begin{aligned} \varvec{\Xi }_{FMA}=&\text {tr} \left\{ \left( \varvec{I}-\frac{\partial \varvec{\sigma }_{0}}{\partial \varvec{\beta }^{T}}\varvec{L}\varvec{V}\right) ^{T}\varvec{V}\left( \varvec{I}-\frac{\partial \varvec{\sigma }_{0}}{\partial \varvec{\beta }^{T}}\varvec{L}\varvec{V}\right) \varvec{\Upsilon } \right\} \end{aligned}$$

with

$$\begin{aligned} \varvec{L} = \left( \frac{\partial \varvec{\mu }_{0}}{\partial \varvec{\theta }^{T}}\varvec{J}_{\theta \theta }^{-1}+\varvec{W}\varvec{K}\varvec{J}_{\theta \gamma }^{T}\varvec{J}_{\theta \theta }^{-1}\right) \left( \frac{\partial \varvec{\sigma }_{0}}{\partial \varvec{\theta }^{T}}\right) ^{T}-\varvec{W}\varvec{K}\left( \frac{\partial \varvec{\sigma }_{0}}{\partial \varvec{\gamma }^{T}}\right) ^{T}. \end{aligned}$$

$T_{FMA-SB-m}$ can be approximated by a Chi-square distribution with *r* degrees of freedom and $T_{FMA-SB-mv}$ can be approximated by a Chi-square distribution with $\left[ \text {tr} \left( \hat{\varvec{\Xi }}_{FMA}\right) \right] ^{2}/ \text {tr} \left( \hat{\varvec{\Xi }}_{FMA}^{2}\right)$ degrees of freedom. The fit indices such as robust RMSEA, CFI, and TLI (Brosseau-Liard & Savalei, 2014; Brosseau-Liard et al., 2012) can also be defined accordingly.

## Simulation Study

In this section, a simulation study is conducted to compare the finite sample properties of FMA with the full model estimation and model selection.

### Simulation Design

The population model is a four-factor SEM, where

$$\begin{aligned} \varvec{\Lambda }_{x}&=\varvec{\Lambda }_{y}=\begin{pmatrix}1.0 &{} 0.95 &{} 0.9 &{} 0 &{} 0 &{} 0\\ 0 &{} 0 &{} 0 &{} 1.0 &{} 0.95 &{} 0.9 \end{pmatrix}^{T}, \\ \text {var}(\varvec{\xi })&=\begin{pmatrix}0.7 &{} 0.3\\ 0.3 &{} 0.7 \end{pmatrix},\quad \varvec{B}=\begin{pmatrix}0.0 &{} 0.0\\ b_{21} &{} 0.0 \end{pmatrix},\quad \varvec{\Gamma }=\begin{pmatrix}0.5 &{} \gamma _{12}\\ \gamma _{21} &{} 0.45 \end{pmatrix}, \end{aligned}$$

Under the local asymptotic framework, we let $\gamma _{12}=\gamma _{0}+\delta /\sqrt{n}$, $\gamma _{21}=\gamma _{0}+0.75\cdot \delta /\sqrt{n}$, and $b_{21}=\gamma _{0}+0.5\cdot \delta /\sqrt{n}$, where $\gamma _{0}=0$ and $\delta =150^{1/2}\zeta$, with $\zeta$ being seven equidistant values between 0 and 0.30 at the step size 0.05. The covariance matrices of $\varvec{\delta }_{x}$ and $\varvec{\delta }_{y}$ are set to be diagonal, of which the diagonal elements make the marginal variances of $\varvec{x}^{*}$ and $\varvec{y}^{*}$ be 1. The distribution of $\varvec{x}^{*}$ and $\varvec{y}^{*}$ is assumed to follow a multivariate normal distribution. These population models are similar to those of Jin and Ankargren (2019), with minor changes of the true values. $\text {var}(\varvec{\varepsilon })$ is set to be a diagonal matrix, and its diagonal elements are 0.45 and 0.5 if the narrow model is the data generation process ($\zeta =0$). For other values of $\zeta$, the diagonal elements are chosen such that the reliability of the measurement models remains the same as the $\zeta =0$.

We fix the number of categories to be five and consider two sets of threshold values. In the first set, the probabilities of belonging to each category are 0.24, 0.41, 0.22, 0.1, and 0.03, which is the moderate asymmetry setting in Rhemtulla et al. (2012). In the second set, the probabilities of belonging to each category are 0.52, 0.15, 0.13, 0.11, and 0.09, which is the extreme asymmetry setting in Rhemtulla et al. (2012). We also take the sample sizes that are used in Rhemtulla et al. (2012), i.e., $n=150$, 350, and $n=600$. The number of replications is set to 10, 000.

Four candidate models are considered in the current study, corresponding to the free parameters in $\varvec{B}$ and $\varvec{\Gamma }$. Model 1, the narrow model, assumes $b_{21}=\gamma _{12}=\gamma _{21}=0$. Model 2 frees $\gamma _{12}$ but assumes $b_{21}=\gamma _{21}=0$. Model 3 freely estimates $\gamma _{12}$ and $\gamma _{21}$, but assumes $b_{21}=0$. Model 4, the full model, freely estimates $b_{21}$, $\gamma _{12}$ and $\gamma _{21}$. Hence, only the full model is the true model if $\zeta \ne 0$. To estimate the local parameter $\varvec{\delta }$, the unbiased estimator $\hat{\varvec{\delta }}=\sqrt{n}\left( \hat{\varvec{\gamma }}_{full}-\varvec{\gamma }_{0}\right)$ is used for simplicity. The parameter of interest is defined to be the vector of free parameters in the full model, excluding the thresholds.

In order to examine the effects of the local asymptotic framework, data are also generated from the standard asymptotic framework, where $\gamma _{12}=\zeta$, $\gamma _{21}=0.75\zeta$, and $b_{21}=0.5\zeta$. The local asymptotic framework coincides with the standard asymptotic framework when $n=150$ but is different when $n=350$ and $n=600$.

The methods considered here include FMA, full model, and model selection. Three implementations of FMA will be considered. The first implementation, denoted by FMAord, is the ordinal data FMA proposed in the current study by accounting for $\text {cov}(\varvec{M},\varvec{D}^T)$. The second implementation, denoted by FMAordcont, uses the weight estimation method and confidence interval from Jin and Ankargren (2019) (i.e., treating $\text {cov}(\varvec{M},\varvec{D}^T) = \varvec{0}$), but ordinal data are still treated as ordinal. Comparing FMAord with FMAordcont allows us to examine the consequence of ignoring a nonzero $\text {cov}(\varvec{M},\varvec{D}^T)$. It is of interest to see whether ordinal data can be treated as continuous in the context of FMA, since it is common that the applied researchers treat ordinal data as continuous. Hence, the third implementation, denoted by FMAcont, treats ordinal data as continuous and directly uses the results from Jin and Ankargren (2019). Since least-squares are used, information criterion is not used for model selection. Rather, we start with whether the narrow model fits the data well by the robust RMSEA (Brosseau-Liard et al., 2012) from lavaan (Rosseel, 2012). In the current simulation, an RMSEA no higher than 0.05 is an indication of a good fit. If the narrow model does not fit the data well, Model 2 is under investigation. If Model 2 does not fit the data well, Model 3 is under investigation. If Model 3 does not fit the data well, the full model is chosen.

All candidate models are estimated in lavaan (Rosseel, 2012) using DWLS. The FMA estimator, confidence interval, and test statistics are programmed in R Core Team (2020). The code can be retrieved from the online appendix.

### Simulation Results

It is likely to encounter non-convergence or Heywood cases when estimating the candidate models. The candidate models that are not converged or have non-positive definite covariance matrices are regarded as inadmissible. The inadmissible candidate models are removed from further analysis. If either the narrow model or full model are inadmissible, the corresponding FMA replication is also considered as inadmissible. Further, outliers are encountered in the current simulation. For simplicity, replications with MSE values that are twice higher than the $99\%$ sample percentile are considered as possible outliers and are removed from further analysis. When $n=150$, at most $0.4\%$ and $1.2\%$ replications are removed if the thresholds are moderately asymmetric and extremely asymmetric, respectively. When $n=600$, at most $0.02\%$ replications are removed. Hence, the percentage of inadmissible solutions are not tabulated here.

**Fig. 1:**
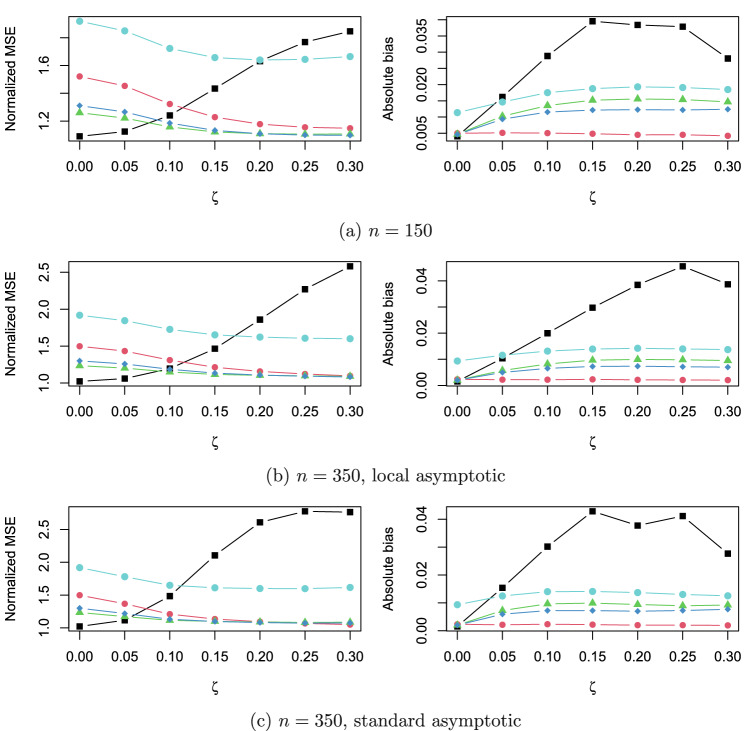
Normalized mean squared error (MSE) and averaged absolute bias of model selection (black square), the full model (red dot), FMAord (green triangle), FMAordcont (blue diamond), and FMAcont (cyan dot) (Color figure online).

To compare the finite sample performance of FMA with model selection and the full model, we compute the normalized MSE, defined as the average of MSE of some method relative to the infeasible minimum MSE across all candidate models, i.e.,

$$\begin{aligned} \frac{1}{R} \sum _{r=1}^{R} \frac{ \text {MSE } \left( \hat{\varvec{\mu }}_{r} - \varvec{\mu }_{true} \right) ^{T} \left( \hat{\varvec{\mu }}_{r} - \varvec{\mu }_{true} \right) \text {at iteration }r }{ \text {Minimum MSE of all admissible candidate models at iteration }r } , \end{aligned}$$

with *R* being the number of replications. We also compute the average of absolute bias $p^{-1}\sum _{i=1}^{p} | \text {median of } \left\{ {\hat{\mu }}_{i,r} - \mu _{i,true}; \, r = 1, \cdots R \right\} |$, where ${\hat{\mu }}_{i,r}$ is the estimate of $\mu _i$ at iteration *r*, $\mu _{i,true}$ is the *i*th entry in $\varvec{\mu }_{true}$, and *p* is the number of parameters. Both the normalized MSE and the averaged bias for moderately asymmetric thresholds are illustrated in Fig. 1. The pattern when $n=600$ is similar to $n=350$ and the pattern for extremely asymmetric thresholds is similar to that for moderately asymmetric thresholds. Hence, they are not reported here due to space limitation. The conclusions that we can draw from Fig. 1 are similar to those in Jin and Ankargren (2019). FMAord tends to yield a lower normalized MSE than the full model, especially when the parameter value is small. Model selection performs well with the lowest normalized MSE when the parameter value is low, whereas it performs the worst when the parameter value is large. This is due to the fact that RMSEA often picks the correct model if the narrow model is the true model, and that RMSEA often implies that a too simple model fits the data well enough. As expected, the cost of MSE reduction is the inclusion of bias. Nevertheless, the induced averaged bias is generally low (Fig. 1), comparing with model selection. It is also seen from Fig. 1 that FMAord tends to have a slightly lower normalized MSE but a higher absolute bias than FMAordcont. A lower normalized MSE is in line with our expectation, since FMAord aims to minimize the correctly derived asymptotic MSE. Further, FMAcont has a higher normalized MSE and a higher absolute bias than FMAord and FMAordcont. Results not presented here show that the normalized MSE and the absolute bias of FMAcont are even higher when the thresholds are extremely asymmetric.

To investigate the coverage probability, the probability of covering $\gamma _{11}=0.5$ when the thresholds are moderately asymmetric is used as an illustration in Table 1. It is seen that the model selection interval is generally accurate when the narrow model is the true model, but is greatly undercovered when the full model is the true model. It is also seen that the FMAord interval performs similar to the full model interval, which is close to the nominal coverage probability $95\%$. These observations are also in line with the findings in Jin and Ankargren (2019). Further, FMAordcont yields a lower coverage probability than FMAord, suggesting that it is important to account for the correlation between $\varvec{M}$ and $\varvec{D}$ when constructing confidence intervals. It is interesting to see that the coverage probability of the FMAcont interval is close to $95\%$ when the thresholds are moderately asymmetric. However, the coverage probability of the FMAcont interval tends to be lower than $95\%$ when the thresholds are extremely asymmetric.

**Table 1:** Coverage probabilities of covering $\gamma _{11}=0.5$ of different methods at the nominal level $95\%$.

Framework	*n*	Method	$\zeta$						
			0.00	0.05	0.10	0.15	0.20	0.25	0.30
Moderately asymmetric thresholds									
Local	150	Model selection	0.92	0.89	0.83	0.75	0.72	0.76	0.81
		Full model	0.92	0.92	0.93	0.92	0.92	0.93	0.93
		FMAord	0.92	0.93	0.93	0.93	0.93	0.93	0.93
		FMAordcont	0.84	0.84	0.84	0.84	0.84	0.84	0.84
		FMAcont	0.93	0.93	0.93	0.93	0.94	0.94	0.94
	600	Model selection	0.94	0.92	0.84	0.72	0.55	0.38	0.30
		Full model	0.94	0.94	0.94	0.94	0.94	0.94	0.94
		FMAord	0.94	0.94	0.94	0.94	0.94	0.94	0.94
		FMAordcont	0.86	0.86	0.86	0.86	0.86	0.85	0.85
		FMAcont	0.93	0.93	0.93	0.93	0.94	0.94	0.94
Standard	600	Model selection	0.94	0.84	0.55	0.30	0.53	0.80	0.85
		Full model	0.94	0.94	0.94	0.94	0.94	0.94	0.94
		FMAord	0.94	0.94	0.94	0.94	0.94	0.94	0.94
		FMAordcont	0.86	0.86	0.86	0.85	0.85	0.85	0.85
		FMAcont	0.93	0.93	0.94	0.94	0.94	0.94	0.93
Extremely asymmetric thresholds									
Local	150	Model selection	0.92	0.90	0.85	0.76	0.71	0.70	0.73
		Full model	0.93	0.93	0.93	0.93	0.93	0.93	0.93
		FMAord	0.93	0.93	0.93	0.93	0.93	0.93	0.93
		FMAordcont	0.86	0.86	0.85	0.85	0.85	0.85	0.86
		FMAcont	0.90	0.90	0.91	0.91	0.91	0.91	0.91
	600	Model selection	0.95	0.93	0.87	0.77	0.62	0.45	0.32
		Full model	0.95	0.95	0.95	0.95	0.95	0.95	0.95
		FMAord	0.94	0.95	0.95	0.95	0.95	0.95	0.95
		FMAordcont	0.86	0.86	0.86	0.86	0.86	0.86	0.86
		FMAcont	0.88	0.88	0.88	0.89	0.89	0.89	0.90
Standard	600	Model selection	0.95	0.87	0.62	0.32	0.33	0.62	0.78
		Full model	0.95	0.95	0.95	0.95	0.95	0.95	0.95
		FMAord	0.94	0.95	0.95	0.95	0.95	0.95	0.94
		FMAordcont	0.86	0.86	0.86	0.86	0.86	0.86	0.86
		FMAcont	0.88	0.88	0.89	0.90	0.90	0.90	0.91

Regarding the goodness-of-fit tests, we only consider the full model, FMAord, and FMAordcont. FMAcont is not considered since the Chi-square test derived in Jin and Ankargren (2019) requires normally distributed data and no robust corrections are derived yet. Table 2 tabulates the empirical rejection rate of the mean-scaled statistic and the mean-and-variance adjusted statistic at the significance level 0.05 when the thresholds are moderately asymmetric. Results for extremely asymmetric thresholds show similar patterns. Hence, they are not reported here. Since the full model is correctly specified, we expect the empirical rejection rate to be approximately 0.05. It is seen that the FMA goodness-of-fit test statistic performs similar to the goodness-of-fit test statistic of the full model, which is in line with Theorem 3 that the goodness-of-fit test statistics are asymptotically equivalent. Comparing the mean-scaled statistic with the mean-and-variance adjusted statistic, the latter tends to have a better size. It is also seen that FMAord and FMAordcont often yield similar empirical sizes, indicating that the effect of $\text {cov}(\varvec{M},\varvec{D}^T)$ is minor when it comes to the goodness-of-fit tests.

**Table 2:** Empirical rejection rate of the goodness-of-fit test statistics at the significance level 0.05, when the thresholds are moderately asymmetric.

Framework	*n*	Method	$\zeta$						
			0.00	0.05	0.10	0.15	0.20	0.25	0.30
Mean-scaled statistic									
Local	150	Full model	0.133	0.126	0.123	0.115	0.115	0.111	0.109
		FMAord	0.141	0.134	0.133	0.128	0.127	0.125	0.123
		FMAordcont	0.141	0.133	0.132	0.124	0.125	0.122	0.120
	350	Full model	0.103	0.101	0.099	0.099	0.095	0.092	0.088
		FMAord	0.106	0.104	0.102	0.105	0.101	0.098	0.094
		FMAordcont	0.106	0.104	0.101	0.103	0.099	0.096	0.092
	600	Full model	0.092	0.092	0.090	0.092	0.091	0.085	0.084
		FMAord	0.093	0.094	0.091	0.094	0.093	0.089	0.088
		FMAordcont	0.093	0.093	0.091	0.093	0.092	0.087	0.086
Standard	350	Full model	0.103	0.099	0.098	0.092	0.088	0.086	0.083
		FMAord	0.106	0.102	0.103	0.097	0.095	0.092	0.090
		FMAordcont	0.106	0.102	0.102	0.095	0.093	0.090	0.088
	600	Full model	0.092	0.090	0.091	0.084	0.081	0.079	0.078
		FMAord	0.093	0.091	0.093	0.088	0.085	0.083	0.082
		FMAordcont	0.093	0.091	0.092	0.086	0.084	0.081	0.081
Mean-and-variance adjusted statistic									
Local	150	Full model	0.068	0.065	0.061	0.060	0.059	0.057	0.057
		FMAord	0.069	0.066	0.064	0.063	0.062	0.061	0.062
		FMAordcont	0.068	0.066	0.062	0.061	0.060	0.060	0.060
	350	Full model	0.058	0.056	0.059	0.057	0.055	0.052	0.052
		FMAord	0.059	0.057	0.060	0.058	0.056	0.054	0.054
		FMAordcont	0.059	0.057	0.059	0.058	0.056	0.053	0.052
	600	Full model	0.054	0.054	0.054	0.054	0.052	0.051	0.050
		FMAord	0.055	0.054	0.055	0.055	0.053	0.053	0.051
		FMAordcont	0.054	0.054	0.054	0.054	0.053	0.052	0.051
Standard	350	Full model	0.058	0.056	0.056	0.053	0.049	0.048	0.048
		FMAord	0.059	0.057	0.058	0.054	0.053	0.051	0.050
		FMAordcont	0.059	0.057	0.057	0.054	0.050	0.050	0.049
	600	Full model	0.054	0.054	0.052	0.050	0.048	0.048	0.049
		FMAord	0.055	0.055	0.053	0.051	0.050	0.051	0.052
		FMAordcont	0.054	0.054	0.053	0.051	0.049	0.049	0.050

## Empirical Example

**Fig. 2:**
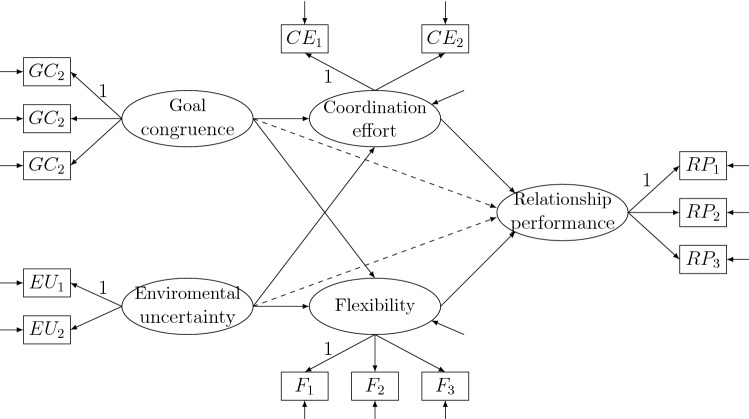
Path diagonal of the seller example. The dashed lines are present in the full model but is omitted in the narrow model.

In this section, an empirical example is analyzed as an illustration. In order to study the supplier–customer relationship, Selnes and Sallis (2003) sampled 780 Scandinavian companies that have more than 50 employees. A total of 665 of them participated in the study. A total of 315 dyads in the sense of supplier and customer are identified. Selnes and Sallis (2003) used a subset of this data set to study how the learning capacity of supplier–customer relationship can be promoted by management. Recently, Sallis (2018) used another subset with 303 dyads to study the effect of relationship flexibility to relationship performance. Two five-factor models under consideration are shown in Fig. 2, which are simplified from Sallis (2018). The narrow model omits the paths from *Goal congruence* to *Relationship performance* and *Environmental uncertainty* to *Relationship performance*, whereas the full model also estimates such two paths. The sample size remained after deleting missing values is $n=266$. All indicators are measured on the 7-point Likert scale. They are aggregated into a 3-point Likert scale that is close to the 3-category extreme asymmetry setting in Rhemtulla et al. (2012). The focus parameter is defined to be the vector of all free parameters needed for the model-implied covariance matrix.

Table 3 tabulates the estimated effects of *Goal congruence* and *Environmental uncertainty* on *Relationship performance*, *Coordination effort*, and *Flexibility*. Model selection chooses the narrow model, since it yields satisfactory fit indices. Nevertheless, all FMA implementations assign non-ignorable weights to the full model, which can be seen from the estimates of the path *Goal congruence* to *Relationship performance*: 0.33 (=0.027/0.083) for FMAord, 0.42 (=0.034/0.083) for FMAordcont, and 0.70 (=0.067/0.083) for FMAcont. FMAord and FMAordcont produce similar point estimates, which can be largely different from those produced by FMAcont. This is in line with our observations from Fig. 1 in the simulation study that FMAcont can be much more biased than FMAord and FMAordcont. Despite similar point estimates, it is also seen that the FMAordcont intervals are generally nested in the FMAord interval, which may lead to a lower coverage probability that we observed in the simulation study. Further, the fit indices and the confidence intervals of the full model are similar to those of FMAord, which is in line with our theoretical results.

**Table 3:** Estimated effects of the latent exogenous latent variables on the endogenous latent variables of the supplier–customer relationship example.

Method	Path		Estimate
	From	To	
Model selection	Goal congruence	Rel. performance	-
		Coordination effort	0.471 (0.250, 0.691)
		Flexibility	0.349 (0.150, 0.548)
	Env. uncertainty	Rel. performance	-
		Coordination effort	0.518 (0.259, 0.777)
		Flexibility	0.467 (0.229, 0.706)
Full model	Goal congruence	Rel. performance	0.083 ($-$0.225, 0.391)
		Coordination effort	0.470 (0.240, 0.700)
		Flexibility	0.352 (0.147, 0.556)
	Env. uncertainty	Rel. performance	0.165 ($-$0.276, 0.605)
		Coordination effort	0.528 (0.253, 0.803)
		Flexibility	0.463 (0.221, 0.705)
FMAord	Goal congruence	Rel. performance	0.027 ($-$0.225,0.391)
		Coordination effort	0.471 (0.237,0.696)
		Flexibility	0.350 (0.145,0.554)
	Env. uncertainty	Rel. performance	0.054 ($-$0.275,0.605)
		Coordination effort	0.521 (0.261,0.810)
		Flexibility	0.466 (0.227,0.710)
FMAordcont	Goal congruence	Rel. performance	0.034 ($-$0.167,0.333)
		Coordination effort	0.471 (0.288,0.647)
		Flexibility	0.350 (0.206,0.494)
	Env. uncertainty	Rel. performance	0.068 ($-$0.209,0.538)
		Coordination effort	0.522 (0.295,0.774)
		Flexibility	0.465 (0.274,0.662)
FMAcont	Goal congruence	Rel. performance	0.067 ($-$0.079,0.268)
		Coordination effort	0.456 (0.260,0.649)
		Flexibility	0.270 (0.122,0.414)
	Env. uncertainty	Rel. performance	0.131 ($-$0.056,0.428)
		Coordination effort	0.471 (0.247,0.710)
		Flexibility	0.387 (0.207,0.571)

Env. uncertainty = Environmental uncertainty. Rel. performance = Relationship performance. The figures in the parentheses are the $95\%$ confidence intervals. For the narrow model, CFI=0.997, TLI=0.997, RMSEA=0.030. For the full model, CFI=0.997, TLI=0.996, RMSEA=0.032. For FMAord, CFI=0.997, TLI=0.996, RMSEA=0.032. For FMAordcont, CFI=0.997, TLI=0.996, RMSEA=0.032. The reported fit indices are robust indices of Brosseau-Liard et al. (2012) and Brosseau-Liard and Savalei (2014).

## Conclusion and Discussion

In this study, FMA is generalized to the SEM with ordinal indicators. We showed that one assumption in Jin and Ankargren (2019), namely $\text {cov}(\varvec{M},\varvec{D}^T) = \varvec{0}$, is violated in SEM with ordinal data. Hence, the results in Jin and Ankargren (2019) for SEM with continuous data need to be revised for SEM with ordinal data. To this end, we derived the correct criterion function for weight estimation, and the valid confidence interval for SEM with ordinal data. To evaluate the global fit, a mean-scaled test statistic and a mean-variance adjusted test statistic are proposed. In the simulation study, we showed that the ordinal data cannot always be treated as continuous, since FMAcont can yield much more biased estimators. We also showed that FMAordcont generally yields similar point estimators to FMAord. However, the FMAordcont interval can be undercovered. Hence, FMAord is still preferred.

Similar to FMA for SEM with continuous indicators, FMA does not uniformly dominate model selection nor the full model in our simulation. The same phenomenon has also been observed in various other models (e.g., Wan et al., 2014; Wang and Zou, 2012; Yang, 2003). This is a general issue for FMA, which is closely related with the combination puzzle (Claeskens et al., 2016) in the forecasting literature. The asymptotic MSE that we aim to minimize is derived under the assumption that the weights are fixed. However, the weights are generally random, if they are estimated from data. Since the uncertainty in the random weights is not accounted for when computing the asymptotic MSE, there is no guarantee that the FMA estimator will be dominating (Claeskens et al., 2016). Nevertheless, as Jin and Ankargren (2019) suggested in the context of SEM with continuous indicators, FMA is a robust compromise between model selection and the full model. Estimators followed by model selection can be unstable (Breiman, 1996) and neither the bias nor the MSE are necessarily bounded (Leeb & Pötscher, 2005). The full model estimator is often unstable (Hjort & Claeskens, 2003a) due to the presence of small parameters. FMA, on the other hand, tends to produce a robust MSE also for SEM with ordinal indicators. Future research is needed to provide guidelines on this matter.

The confidence interval considered in this paper is the Hjort and Claeskens (2003a) type, which is asymptotically equivalent to the full model confidence interval. Various other model-averaging confidence interval have also emerged in the literature, such as (Fletcher and Dillingham (2011), Fletcher and Turek (2011), and Turek and Fletcher (2012)). Their properties have been investigated by Kabaila et al. (2016), Kabaila et al. (2017), and Kabaila (2018). Nevertheless, it is difficult to outperform the full model confidence interval (Kabaila et al., 2016). Wang and Zou (2012) suggested the use of the full model interval since it is computationally easy and the FMA interval does not offer a major improvement. Following these suggestions, both the full model interval and the FMA interval will offer valid inference in practice. Even if the point estimator is taken from one candidate model, Jin and Ankargren (2019) still suggested to use the full model interval or the FMA interval to take the selection uncertainty into consideration. The same suggestion applies to the SEM with ordinal data.

One limitation of the proposed goodness-of-fit statistic is that the focus parameter $\varvec{\mu }$ ought to be the vector of all free parameters in $\varvec{\Sigma }$, an assumption needed for Theorem 3. If $\varvec{\mu } \ne \varvec{\beta }$ such as $\varvec{\mu }=(\varvec{I} - \varvec{B} )^{-1} \varvec{\Gamma }$, we can still obtain valid FMA estimate $\bar{\varvec{\mu }}$ and construct valid confidence intervals for $\varvec{\mu }$. However, the proposed test statistic is not guaranteed to be asymptotically equivalent to the full model test statistic. Further studies will be devoted to the model evaluation for a general parameter vector of interest.

## Supplementary Information

Below is the link to the electronic supplementary material.

Supplementary file 1 (pdf 651 KB)

## Footnotes

1 For the purpose of presentation, the notations in Jin and Ankargren (2019) are modified. $\varvec{J}_{full}$, $\varvec{M}$, and $\varvec{N}$ here corresponds to $\varvec{J}_{full}/4$, $\varvec{M}/2$, and $\varvec{N}/2$ in Jin and Ankargren (2019).
