# Supplementary material for: Frequentist Model Averaging in Structure Equation Model With Ordinal Data
Source: Psychometrika. 2022 Jan 29;87(3):1130–45. doi: 10.1007/s11336-021-09837-3 (PMC9433363; doi:10.1007/s11336-021-09837-3)
Supplement: Supplementary file 1 — (pdf 651 KB) [file 11336_2021_9837_MOESM1_ESM.pdf]

Frequentist Model Averaging in Structure Equation Model With Ordinal Data: Supplementary Material

Frequentist Model Averaging in Structure Equation Model With Ordinal Data: Supplementary Material

1 Introduction

This supplementary material contains mathematical proofs of the main results in the paper. The reader is directed to the main text for the explanations of the notations.

Suppose that there exists a $q \times 1$ vector of underlying continuous random variables $\boldsymbol{x}^*$ that follows a multivariate normal distribution with variances 1. Let $\boldsymbol{x}$ be the vector of discrete counterparts of $\boldsymbol{x}^*$, x_g the gth entry of $\boldsymbol{x}$, and m_g the number of possible outcomes that x_g can take. Then, $x_g = a$ if $\tau_{g,a-1} < x_g^* \leq \tau_{g,a}$, where x_g^* is the gth underlying continuous random variable, $\tau_{g,0} \equiv -\infty$, and $\tau_{g,m_g} \equiv \infty$. The vector of unknown thresholds for x_g is denoted by $\boldsymbol{\tau}_g$. The probability of observing $\boldsymbol{x} = \begin{pmatrix} a_1 & a_2 & \cdots & a_q \end{pmatrix}$ is

$$\pi_{a_1 a_2 \cdots a_q}(\boldsymbol{\tau}, \boldsymbol{\rho}) = \int_{\tau_{1,a_1-1}}^{\tau_{1,a_1}} \int_{\tau_{2,a_2-1}}^{\tau_{2,a_2}} \cdots \int_{\tau_{q,a_q-1}}^{\tau_{q,a_q}} \phi_q(t_1, ..., t_q; \boldsymbol{\rho})\, dt_q \cdots dt_1,$$

where $\boldsymbol{\tau} = \left(\boldsymbol{\tau}_1^T, \boldsymbol{\tau}_2^T \cdots \boldsymbol{\tau}_q^T\right)^T$ is the vector of all thresholds, $\boldsymbol{\rho}$ is the vector of all unique polychoric correlation coefficients, and $\phi_q(;\boldsymbol{\rho})$ is the density function of a q-dimensional normal random variable with means 0, variances 1, and correlation coefficients $\boldsymbol{\rho}$. Let $\boldsymbol{\tau}_{gh}^T = \left(\boldsymbol{\tau}_g^T, \boldsymbol{\tau}_h^T\right)$. The probability of observing $x_g = a$ and $x_h = b$ is

$$\pi_{ab}^{(gh)}(\boldsymbol{\tau}_{gh}, \rho_{gh}) = \int_{\tau_{g,a-1}}^{\tau_{g,a}} \int_{\tau_{h,b-1}}^{\tau_{h,b}} \phi_2(t_1, t_2; \rho_{gh})\, dt_2 dt_1, \tag{1}$$

where ρ_{gh} is the polychoric correlation coefficient between x_g and x_h; and $\phi_2(;\rho)$ is the density function of a bivariate normal random variable with means 0, variances 1, and correlation ρ. Likewise, we can let $\pi_{a_g a_h a_s a_t}^{(ghst)}(\boldsymbol{\tau}_{ghst}, \boldsymbol{\rho}_{ghst})$ be the probability of observing $\begin{pmatrix} x_g & x_h & x_s & x_t \end{pmatrix} = \begin{pmatrix} a_g & a_h & a_s & a_t \end{pmatrix}$ and $\pi_{a_g a_h a_s}^{(ghs)}(\boldsymbol{\tau}_{ghs}, \boldsymbol{\rho}_{ghs})$ be the probability of

observing $\begin{pmatrix} x_g & x_h & x_s \end{pmatrix} = \begin{pmatrix} a_g & a_h & a_s \end{pmatrix}$, where

$$\begin{aligned}
\boldsymbol{\tau}_{ghst} &= \begin{pmatrix} \boldsymbol{\tau}_g^T & \boldsymbol{\tau}_h^T & \boldsymbol{\tau}_s^T & \boldsymbol{\tau}_t^T \end{pmatrix}^T, \\
\boldsymbol{\rho}_{ghst} &= \begin{pmatrix} \rho_{gh} & \rho_{gs} & \rho_{gt} & \rho_{hs} & \rho_{ht} & \rho_{st} \end{pmatrix}^T, \\
\boldsymbol{\tau}_{ghs} &= \begin{pmatrix} \boldsymbol{\tau}_g^T & \boldsymbol{\tau}_h^T & \boldsymbol{\tau}_s^T \end{pmatrix}^T, \\
\boldsymbol{\rho}_{ghs} &= \begin{pmatrix} \rho_{gh} & \rho_{gs} & \rho_{hs} \end{pmatrix}^T.
\end{aligned}$$

Throughout the paper, we estimate the polychoric correlation coefficient using the two-step procedure of Olsson (1979). First, the thresholds are estimated from the univariate standard normal distribution. Second, the correlation coefficient is estimated conditional on the estimated thresholds. The multinomial log-likelihood function in the second step is

$$\ell_{gh}\left(\boldsymbol{\tau}_{gh}, \rho_{gh}\right) = \sum_{i=1}^{n} \ell_{gh,i}\left(\boldsymbol{\tau}_{gh}, \rho_{gh}\right),$$

where n is the sample size, $\boldsymbol{\tau}_{gh}$ is fixed to the first-step estimates $\hat{\boldsymbol{\tau}}_{gh}$, and

$$\begin{aligned}
\ell_{gh,i}\left(\boldsymbol{\tau}_{gh}, \rho_{gh}\right) &= \sum_{a=1}^{m_g}\sum_{b=1}^{m_h} I_{gh,i}\left(a,b\right) \log \pi_{ab}^{(gh)}\left(\boldsymbol{\tau}_{gh}, \rho_{gh}\right), \\
I_{gh,i}\left(a,b\right) &= \begin{cases} 1, & \text{if we observe } x_{g,i} = a \text{ and } x_{h,i} = b, \\ 0, & \text{otherwise.} \end{cases}
\end{aligned}$$

Here, $\{\ell_{gh,i}\left(\boldsymbol{\tau}_{gh}, \rho_{gh}\right)\}$ are mutually independent.

2 Polychoric Correlation Estimation

2.1 Standard Asymptotic Framework

Under the standard asymptotic framework, we assume that the true values of the polychoric correlations do not depend on the sample size n. Most SEM related studies lie within the standard asymptotic framework. Suppose that the true value of $\boldsymbol{\rho}$ is $\boldsymbol{\rho}_0$. Under some conditions, Jöreskog (1994) showed that

$$\sqrt{n}\left(\hat{\boldsymbol{\rho}} - \boldsymbol{\rho}_0\right) \overset{d}{\to} N\left(\mathbf{0}, \boldsymbol{\Upsilon}\right), \tag{2}$$

where $\boldsymbol{\Upsilon}$ is the asymptotic covariance matrix. The expression of $\boldsymbol{\Upsilon}$ can be obtained from Jöreskog (1994).

2.2 Local Asymptotic Framework

Under the local asymptotic framework, we assume that the true values of some parameters depend on the sample size n. Our paper assumes that the true value of $\boldsymbol{\gamma}$ is $\boldsymbol{\gamma}_0 + \boldsymbol{\delta}/\sqrt{n}$. Hence, the true model-implied $\boldsymbol{\sigma}$ is of the form

$$\boldsymbol{\sigma}_{true} = \boldsymbol{\sigma}\left(\boldsymbol{\beta}_{true}\right) = \boldsymbol{\sigma}\left(\boldsymbol{\theta}_0, \boldsymbol{\gamma}_0 + \boldsymbol{\delta}/\sqrt{n}\right).$$

Suppose that $\boldsymbol{\sigma}_{true}$ can be expanded into

$$\boldsymbol{\sigma}_{true} = \boldsymbol{\sigma}_0 + \boldsymbol{\delta}^*/\sqrt{n} + O\left(n^{-1}\right), \tag{3}$$

where $\boldsymbol{\sigma}_0 = \boldsymbol{\sigma}\left(\boldsymbol{\theta}_0, \boldsymbol{\gamma}_0\right)$ and

$$\boldsymbol{\delta}^* = \frac{\partial \boldsymbol{\sigma}\left(\boldsymbol{\theta}_0, \boldsymbol{\gamma}_0\right)}{\partial \boldsymbol{\gamma}^T}\boldsymbol{\delta}.$$

Then, for any pair (g, h), the polychoric correlation coefficient between x_g and x_h, denoted by ρ_{gh}, is also locally drifted. Its true value satisfies

$$\rho_{gh,true} = \rho_{gh,0} + \delta^*_{gh}/\sqrt{n} + O\left(n^{-1}\right)$$

for some local parameter δ^*_{gh}. However, the thresholds $\boldsymbol{\tau}_g$ and $\boldsymbol{\tau}_h$ are not locally drifted. Consequently, the true probability of observing $x_g = a$ and $x_h = b$ becomes $\pi_{ab}^{(gh)}\left(\boldsymbol{\tau}_{gh,0}, \rho_{gh,true}\right)$, where the subscript 0 is used to denote the true value of the thresholds.

2.3 Regularity Conditions

Let $\bar{O}^{(\rho)}$ and $\bar{O}_g^{(\tau)}$ be closed balls that contain $\boldsymbol{\rho}_0$ and $\boldsymbol{\tau}_{g,0}$ as interior points, respectively. Suppose that the polychoric correlation matrix with the correlation coefficients $\boldsymbol{\rho}_0$ is a positive definite matrix; and that all $\pi_{a_1 a_2 \cdots a_q}\left(\boldsymbol{\tau}_0, \boldsymbol{\rho}_0\right) > 0$. Because the polychoric correlation matrix is a continuous function of $\boldsymbol{\rho}$ and $\pi_{a_1 a_2 \cdots a_q}\left(\boldsymbol{\tau}, \boldsymbol{\rho}\right)$ is a

continuous function of $(\boldsymbol{\tau}, \boldsymbol{\rho})$, we can choose $\bar{O}^{(\rho)}$ and $\bar{O}_g^{(\tau)}$ such that the polychoric correlation matrix remains positive definite and $\pi_{a_1 a_2 \cdots a_q}(\boldsymbol{\tau}, \boldsymbol{\rho})$ is bounded below from zero for all $(\boldsymbol{\tau}, \boldsymbol{\rho}) \in \bar{O}^{(\tau)} \times \bar{O}^{(\rho)}$, where $\bar{O}^{(\tau)} = \bar{O}_1^{(\tau)} \times \bar{O}_2^{(\tau)} \times \cdots \times \bar{O}_q^{(\tau)}$ and $\times$ is the Cartesian product. Recall that a compact set is closed and bounded in the Euclidean space. Then by the Tychonoff theorem (e.g., Richmond, 2020, Theorem 6.2.5), $\bar{O}^{(\tau)}$ and $\bar{O}^{(\tau)} \times \bar{O}^{(\rho)}$ are also closed and bounded. By the extreme value theorem (e.g., Rudin, 1976, Theorem 4.16), any continuous function will attain its minimum and maximum on $\bar{O}^{(\tau)} \times \bar{O}^{(\rho)}$. Thus, we can find open balls $O^{(\tau)} \subset \bar{O}^{(\tau)}$ and $O^{(\rho)} \subset \bar{O}^{(\rho)}$ such that $\boldsymbol{\tau}_0$ is an interior point of $O^{(\tau)}$, $\boldsymbol{\rho}_0$ is an interior point of $O^{(\rho)}$, and the continuous functions that are bounded on $\bar{O}^{(\tau)} \times \bar{O}^{(\rho)}$ are still bounded on $O^{(\tau)} \times O^{(\rho)}$.

Before we proceed to the main results and their proofs, we need to establish some properties regarding the partial derivatives of $\log \pi_{a_1 \cdots a_q}(\boldsymbol{\tau}, \boldsymbol{\rho})$ for $(\boldsymbol{\tau}, \boldsymbol{\rho}) \in O^{(\tau)} \times O^{(\rho)}$.

Result 1. *For any finite dimension $q \geq 2$, $\log \pi_{a_1 \cdots a_q}(\boldsymbol{\tau}, \boldsymbol{\rho})$, the first-order partial derivatives of $\log \pi_{a_1 \cdots a_q}(\boldsymbol{\tau}, \boldsymbol{\rho})$, the second-order partial derivatives of $\log \pi_{a_1 \cdots a_q}(\boldsymbol{\tau}, \boldsymbol{\rho})$, and the second-order partial derivatives of $\partial \log \pi_{a_1 \cdots a_q}(\boldsymbol{\tau}, \boldsymbol{\rho}) / \partial \boldsymbol{\rho}$ are all bounded on $O^{(\tau)} \times O^{(\rho)}$.*

Proof. The boundness of $\log \pi_{a_1 \cdots a_q}(\boldsymbol{\tau}, \boldsymbol{\rho})$ holds since $\pi_{a_1 \cdots a_q}(\boldsymbol{\tau}, \boldsymbol{\rho}) > 0$ is bounded on the open set $O^{(\tau)} \times O^{(\rho)}$ by construction. Regarding the first-order and higher-order partial derivatives of $\log \pi_{a_1 \cdots a_q}(\boldsymbol{\tau}, \boldsymbol{\rho})$, $\pi_{a_1 a_2 \cdots a_q}(\boldsymbol{\tau}, \boldsymbol{\rho})$ is bounded below from zero for all $(\boldsymbol{\tau}, \boldsymbol{\rho}) \in O^{(\tau)} \times O^{(\rho)}$. Then we only need to show that the derivatives of $\pi_{a_1 \cdots a_q}(\boldsymbol{\tau}, \boldsymbol{\rho})$ are bounded. Since $\pi_{a_1 \cdots a_q}(\boldsymbol{\tau}, \boldsymbol{\rho})$ involves a multiple integral, the partial derivatives of $\pi_{a_1 \cdots a_q}(\boldsymbol{\tau}, \boldsymbol{\rho})$ is also a multiple integral. For some constants d_k, some vectors $\boldsymbol{a}_k$ and some

matrices $\boldsymbol{A}_k$, we have

$$\begin{aligned} & \left| \int_{\tau_{1,a_1-1}}^{\tau_{1,a_1}} \cdots \int_{\tau_{q,a_q-1}}^{\tau_{q,a_q}} \phi_q(\boldsymbol{t};\boldsymbol{\rho}) \prod_k \left(d_k + \boldsymbol{a}_k^T \boldsymbol{t} + \boldsymbol{t}^T \boldsymbol{A}_k \boldsymbol{t}\right) dt_q \cdots dt_1 \right| \\ \leq & \int_{\tau_{1,a_1-1}}^{\tau_{1,a_1}} \cdots \int_{\tau_{q,a_q-1}}^{\tau_{q,a_q}} \phi_q(\boldsymbol{t};\boldsymbol{\rho}) \prod_k \left| d_k + \boldsymbol{a}_k^T \boldsymbol{t} + \boldsymbol{t}^T \boldsymbol{A}_k \boldsymbol{t} \right| dt_q \cdots dt_1 \\ \leq & \ E \left| \prod_k \left(d_k + \boldsymbol{a}_k^T \boldsymbol{t} + \boldsymbol{t}^T \boldsymbol{A}_k \boldsymbol{t}\right) \right|, \end{aligned} \tag{4}$$

where $\boldsymbol{t} = (t_1, \cdots, t_q)^T$. That is, the above integral is bounded by a linear combination of absolute moments of a multivariate normal distribution. Kamat (1953) have proposed an approach to compute those absolute moments. Then, (4) is bounded on $O^{(\tau)} \times O^{(\rho)}$, if d_k, $\boldsymbol{a}_k$ and $\boldsymbol{A}_k$ are all continuous functions of $(\boldsymbol{\tau}, \boldsymbol{\rho})$. Thus, it suffices to show that the integrand needed to compute the partial derivatives are $\phi_q(\boldsymbol{t};\boldsymbol{\rho})$ multiplied by polynomials of $\boldsymbol{t}$.

Let ρ_g be the gth entry of $\boldsymbol{\rho}$ and $\boldsymbol{\Sigma}$ be a correlation matrix with off-diagonal elements $\boldsymbol{\rho}$. That is, a random variable with density function $\phi_q(\boldsymbol{t};\boldsymbol{\rho})$ has the correlation matrix $\boldsymbol{\Sigma}$. Let σ_{ij} be the (i,j)th entry of $\boldsymbol{\Sigma}$,

$$\boldsymbol{\Sigma}^{(g)} = \boldsymbol{\Sigma}^{-1} \frac{\partial \boldsymbol{\Sigma}}{\partial \rho_g} \boldsymbol{\Sigma}^{-1},$$

and $\sigma_{ij}^{(g)}$ be the (i,j)th entry of $\boldsymbol{\Sigma}^{(g)}$. For any g,

$$\frac{\partial \pi_{a_1 \cdots a_q}(\boldsymbol{\tau}, \boldsymbol{\rho})}{\partial \rho_g} = \int_{\tau_{1,a_1-1}}^{\tau_{1,a_1}} \cdots \int_{\tau_{q,a_q-1}}^{\tau_{q,a_q}} \phi_q(\boldsymbol{t};\boldsymbol{\rho}) \frac{\partial \log \phi_q(\boldsymbol{t};\boldsymbol{\rho})}{\partial \rho_g} dt_q \cdots dt_1,$$

where

$$\frac{\partial \log \phi_q(\boldsymbol{t};\boldsymbol{\rho})}{\partial \rho_g} = -\frac{1}{2} \mathrm{tr} \left\{ \boldsymbol{\Sigma} \boldsymbol{\Sigma}^{(g)} \right\} + \frac{1}{2} \boldsymbol{t}^T \boldsymbol{\Sigma}^{(g)} \boldsymbol{t}.$$

Thus, $\partial \pi_{a_1 \cdots a_q}(\boldsymbol{\tau}, \boldsymbol{\rho}) / \partial \rho_g$ is bounded on $O^{(\tau)} \times O^{(\rho)}$. Regarding the first-order partial

derivative with respect to $\boldsymbol{\tau}$, note that

$$\begin{aligned}\left|\frac{\partial \pi_{a_1\cdots a_q}(\boldsymbol{\tau},\boldsymbol{\rho})}{\partial \tau_{1,a}}\right| &= \int_{\tau_{2,a_2-1}}^{\tau_{2,a_2}}\cdots\int_{\tau_{q,a_q-1}}^{\tau_{q,a_q}} \phi_q\left(\tau_{1,a},t_2,\cdots,t_q;\boldsymbol{\rho}\right)dt_q\cdots dt_2\\ &\le \phi_1\left(\tau_{1,a}\right)\le \frac{1}{\sqrt{2\pi}},\ \text{if } \tau_{1,a}=\tau_{1,a_1} \text{ or } \tau_{1,a_1-1},\\ \left|\frac{\partial \pi_{a_1\cdots a_q}(\boldsymbol{\tau},\boldsymbol{\rho})}{\partial \tau_{1,a}}\right| &= 0,\ \text{if } \tau_{1,a}\neq\tau_{1,a_1} \text{ and } \tau_{1,a}\neq\tau_{1,a_1-1},\end{aligned}$$

where $\phi_1()$ is the density of a univariate standard normal random variable. Therefore, the first-order partial derivatives of $\pi_{a_1\cdots a_q}(\boldsymbol{\tau},\boldsymbol{\rho})$ with respect to $\boldsymbol{\tau}_1$ are bounded. By symmetry in the sense that the order of the integral can be interchanged, the first-order partial derivatives of $\pi_{a_1\cdots a_q}(\boldsymbol{\tau},\boldsymbol{\rho})$ with respect to $\boldsymbol{\tau}$ are bounded. Consequently, the first-order partial derivatives of $\log \pi_{a_1\cdots a_q}(\boldsymbol{\tau},\boldsymbol{\rho})$ are bounded on $O^{(\tau)}\times O^{(\rho)}$.

Now consider the first-order partial derivatives of $\partial \pi_{a_1\cdots a_q}(\boldsymbol{\tau},\boldsymbol{\rho})/\partial\boldsymbol{\rho}$. Note that

$$\frac{\partial^2 \pi_{a_1\cdots a_q}(\boldsymbol{\tau},\boldsymbol{\rho})}{\partial\rho_g\partial\rho_h} = \int_{\tau_{1,a_1-1}}^{\tau_{1,a_1}}\cdots\int_{\tau_{q,a_q-1}}^{\tau_{q,a_q}} \frac{\partial^2\phi_q(\boldsymbol{t};\boldsymbol{\rho})}{\partial\rho_g\partial\rho_h}dt_q\cdots dt_1,$$

where

$$\begin{aligned}\frac{\partial^2\phi_q(\boldsymbol{t};\boldsymbol{\rho})}{\partial\rho_g\partial\rho_h} &= \phi_q(\boldsymbol{t};\boldsymbol{\rho})\left(\frac{1}{4}\mathrm{tr}\left\{\boldsymbol{\Sigma}\boldsymbol{\Sigma}^{(g)}\right\}\mathrm{tr}\left\{\boldsymbol{\Sigma}\boldsymbol{\Sigma}^{(h)}\right\}-\frac{1}{2}\mathrm{tr}\left\{\frac{\partial^2\boldsymbol{\Sigma}}{\partial\rho_g\partial\rho_h}\boldsymbol{\Sigma}^{-1}-\boldsymbol{\Sigma}\boldsymbol{\Sigma}^{(g)}\boldsymbol{\Sigma}\boldsymbol{\Sigma}^{(h)}\right\}\right)\\ &+\frac{1}{4}\phi_q(\boldsymbol{t};\boldsymbol{\rho})\boldsymbol{t}^T\left(2\frac{\partial\boldsymbol{\Sigma}^{(g)}}{\partial\rho_h}-\mathrm{tr}\left\{\boldsymbol{\Sigma}\boldsymbol{\Sigma}^{(g)}\right\}\boldsymbol{\Sigma}^{(h)}-\mathrm{tr}\left\{\boldsymbol{\Sigma}\boldsymbol{\Sigma}^{(h)}\right\}\boldsymbol{\Sigma}^{(g)}\right)\boldsymbol{t}\\ &+\frac{1}{4}\phi_q(\boldsymbol{t};\boldsymbol{\rho})\left(\boldsymbol{t}^T\boldsymbol{\Sigma}^{(h)}\boldsymbol{t}\right)\left(\boldsymbol{t}^T\boldsymbol{\Sigma}^{(g)}\boldsymbol{t}\right).\end{aligned}$$

Hence, $\partial^2\pi_{a_1\cdots a_q}(\boldsymbol{\tau},\boldsymbol{\rho})/\partial\rho_g\partial\rho_h$ is bounded on $O^{(\tau)}\times O^{(\rho)}$. Regarding the first-order partial derivatives of $\partial\log\pi_{a_1\cdots a_q}(\boldsymbol{\tau},\boldsymbol{\rho})/\partial\boldsymbol{\rho}$ with respect to $\boldsymbol{\tau}$, we still use the derivatives with respect to $\boldsymbol{\tau}_1$ as an example. It is easy to see that $\partial^2\pi_{a_1\cdots a_q}(\boldsymbol{\tau},\boldsymbol{\rho})/\partial\tau_{1,a}\partial\rho_h=0$ if $a\neq a_1-1$ and $a\neq a_1$. If $a=a_1-1$ or a_1, then

$$\left|\frac{\partial^2\pi_{a_1\cdots a_q}(\boldsymbol{\tau},\boldsymbol{\rho})}{\partial\tau_{1,a}\partial\rho_h}\right| = \left|\int_{\tau_{2,a_2-1}}^{\tau_{2,a_2}}\cdots\int_{\tau_{q,a_q-1}}^{\tau_{q,a_q}}\left[\frac{1}{2}\phi_q\left(\tilde{\boldsymbol{t}}_a;\boldsymbol{\rho}\right)\left(\tilde{\boldsymbol{t}}_a^T\boldsymbol{\Sigma}^{(g)}\tilde{\boldsymbol{t}}_a\right)-\frac{1}{2}\phi_q\left(\tilde{\boldsymbol{t}}_a;\boldsymbol{\rho}\right)\mathrm{tr}\left\{\boldsymbol{\Sigma}\boldsymbol{\Sigma}^{(g)}\right\}\right]dt_q\cdots dt_2\right|,$$

where $\tilde{\boldsymbol{t}}_a = \begin{pmatrix} \tau_{1,a} & t_2 & \cdots & t_q \end{pmatrix}^T$. Hence, $\partial^2 \pi_{a_1 \cdots a_q}(\boldsymbol{\tau}, \boldsymbol{\rho}) / \partial \tau_{1,a} \partial \rho_h$ is bounded on $O^{(\tau)} \times O^{(\rho)}$ if $a = a_{1-1}$ or a_1. Regarding the first-order partial derivatives of $\partial \pi_{a_1 \cdots a_q}(\boldsymbol{\tau}, \boldsymbol{\rho}) / \partial \tau_{1,a}$ with respect to $\boldsymbol{\tau}$, it is easy to see that $\partial^2 \pi_{a_1 \cdots a_q}(\boldsymbol{\tau}, \boldsymbol{\rho}) / \partial \tau_{1,a_1} \partial \tau_{1,a} = 0$ if $a \neq a_1$. If $a = a_1$,

$$\frac{\partial^2 \pi_{a_1 \cdots a_q}(\boldsymbol{\tau}, \boldsymbol{\rho})}{\partial \tau_{1,a_1}^2} = \int_{\tau_{2,a_2-1}}^{\tau_{2,a_2}} \cdots \int_{\tau_{q,a_q-1}}^{\tau_{q,a_q}} \phi_q(\tau_{1,a_1}, t_2, \cdots, t_q; \boldsymbol{\rho}) \left(-\boldsymbol{e}_1^T \boldsymbol{\Sigma}^{-1} \tilde{\boldsymbol{t}}_{a_1}\right) dt_q \cdots dt_2,$$

where $\boldsymbol{e}_i$ is a vector with ith entry being 1 and other entries being 0. Hence, $\partial^2 \pi_{a_1 \cdots a_q}(\boldsymbol{\tau}, \boldsymbol{\rho}) / \partial \tau_{1,a_1}^2$ is bounded if $a = a_1$. Further, $\partial^2 \pi_{a_1 \cdots a_q}(\boldsymbol{\tau}, \boldsymbol{\rho}) / \partial \tau_{1,a_1} \partial \tau_{2,a} = 0$, if $a \neq a_2 - 1$ and $a \neq a_2$. If $a = a_2 - 1$ or a_2, then

$$\left| \frac{\partial^2 \pi_{a_1 \cdots a_q}(\boldsymbol{\tau}, \boldsymbol{\rho})}{\partial \tau_{1,a_1} \partial \tau_{2,a}} \right| = \left| \int_{\tau_{3,a_3-1}}^{\tau_{3,a_3}} \cdots \int_{\tau_{q,a_q-1}}^{\tau_{q,a_q}} \phi_q(\tau_{1,a_1}, \tau_{2,a}, t_3, \cdots, t_q; \boldsymbol{\rho}) \, dt_q \cdots dt_3 \right| \leq \phi_2(\tau_{1,a_1}, \tau_{2,a}; \rho_{12}).$$

Therefore, by symmetry, every entry in $\partial^2 \pi_{a_1 \cdots a_q}(\boldsymbol{\tau}, \boldsymbol{\rho}) / \partial \boldsymbol{\tau} \partial \boldsymbol{\tau}^T$ are bounded on $O^{(\tau)} \times O^{(\rho)}$. This completes the proof for the boundness of the second-order partial derivatives of $\log \pi_{a_1 \cdots a_q}(\boldsymbol{\tau}, \boldsymbol{\rho})$.

At last consider the second-order partial derivatives of $\partial \pi_{a_1 \cdots a_q}(\boldsymbol{\tau}, \boldsymbol{\rho}) / \partial \boldsymbol{\rho}$. From $\partial^2 \pi_{a_1 \cdots a_q}(\boldsymbol{\tau}, \boldsymbol{\rho}) / \partial \rho_g \partial \rho_h$ derived above, the integrand needed in $\partial^3 \pi_{a_1 \cdots a_q}(\boldsymbol{\tau}, \boldsymbol{\rho}) / \partial \rho_g \partial \rho_h \partial \rho_i$ is $\phi_q(\boldsymbol{t}; \boldsymbol{\rho})$ multiplied by a polynomial of $\boldsymbol{t}$ up to the sixth order. From $\partial^2 \pi_{a_1 a_2 \cdots a_q}(\boldsymbol{\tau}, \boldsymbol{\rho}) / \partial \tau_{1,a} \partial \rho_h$ derived above, the integrand needed in $\partial^3 \pi_{a_1 \cdots a_q}(\boldsymbol{\tau}, \boldsymbol{\rho}) / \partial \tau_{1,a} \partial \rho_g \partial \rho_h$ is $\phi_q(\boldsymbol{t}; \boldsymbol{\rho})$ multiplied by a polynomial of $\boldsymbol{t}$ up to the fourth order. From $\partial^2 \pi_{a_1 a_2 \cdots a_q}(\boldsymbol{\tau}, \boldsymbol{\rho}) / \partial \boldsymbol{\tau} \partial \boldsymbol{\tau}^T$derived above, the integrands needed in $\partial^3 \pi_{a_1 \cdots a_q}(\boldsymbol{\tau}, \boldsymbol{\rho}) / \partial \boldsymbol{\tau} \partial \boldsymbol{\tau}^T \partial \rho_g$ is also a multivariate normal density multiplied by a polynomial of $\boldsymbol{t}$ up to the fourth order. Therefore, the second-order partial derivatives of $\partial \log \pi_{a_1 \cdots a_q}(\boldsymbol{\tau}, \boldsymbol{\rho}) / \partial \boldsymbol{\rho}$ are bounded on $O^{(\tau)} \times O^{(\rho)}$.

□

In order to show the main results, we need the following regularity conditions.

A1 For any pair (g, h), $\rho_{gh,true} = \rho_{gh,0} + \delta_{gh}^* / \sqrt{n} + O(n^{-1})$ for some local parameter δ_{gh}^*.

A2 For any $(\boldsymbol{\tau}_{gh}, \rho_{gh}) \neq (\boldsymbol{\tau}_{gh,0}, \rho_{gh,0})$, $P\left(\ell_{gh,i}\left(\boldsymbol{\tau}_{gh}, \rho_{gh}\right) = \ell_{gh,i}\left(\boldsymbol{\tau}_{gh,0}, \rho_{gh,0}\right)\right) < 1$.

A3 For any pair (g, h), $\partial \ell_{gh}\left(\boldsymbol{\tau}_{gh}, \rho_{gh}\right)/\partial \rho_{gh} = 0$ always has a unique solution for any $\boldsymbol{\tau}_{gh} \in O_g^{(\tau)} \times O_h^{(\tau)}$.

A4 For any pair (g, h), the information

$$E_0\left(\frac{\partial^2 \ell_{gh,i}\left(\boldsymbol{\tau}_{gh,0}, \rho_{gh,0}\right)}{\partial \rho^2}\right) = \sum_{a=1}^{m_g}\sum_{b=1}^{m_h} \pi_{ab}\left(\boldsymbol{\tau}_{gh,0}, \rho_{gh,0}\right) \frac{\partial^2 \log \pi_{ab}\left(\boldsymbol{\tau}_{gh,0}, \rho_{gh,0}\right)}{\partial \rho^2} \neq 0.$$

Assumptions A2 and A3 ensure the identifibility. Assumption A4 ensures that the reciprocal of the Fisher information is well-defined. Assumption A1 means that the true values of the polychoric correlation coefficients are locally drifted in a $n^{-1/2}$ neighborhood. This is a mild assumption when the true values of the structural equation model in the main text are locally drifted. The model-implied covariance matrix of the structural equation model in the main text is

$$\begin{pmatrix} \boldsymbol{\Lambda}_y \left(\boldsymbol{I}-\boldsymbol{B}\right)^{-1}\left[\boldsymbol{\Gamma}\mathrm{var}\left(\boldsymbol{\xi}\right)\boldsymbol{\Gamma}^T + \mathrm{var}\left(\boldsymbol{\varepsilon}\right)\right]\left[\left(\boldsymbol{I}-\boldsymbol{B}\right)^{-1}\right]^T \boldsymbol{\Lambda}_y^T & \boldsymbol{\Lambda}_y\left(\boldsymbol{I}-\boldsymbol{B}\right)^{-1}\boldsymbol{\Gamma}\mathrm{var}\left(\boldsymbol{\xi}\right)\boldsymbol{\Lambda}_x^T \\ \boldsymbol{\Lambda}_x \mathrm{var}\left(\boldsymbol{\xi}\right)\boldsymbol{\Gamma}^T\left[\left(\boldsymbol{I}-\boldsymbol{B}\right)^{-1}\right]^T \boldsymbol{\Lambda}_y^T & \boldsymbol{\Lambda}_x \mathrm{var}\left(\boldsymbol{\xi}\right)\boldsymbol{\Lambda}_x^T \end{pmatrix} + \mathrm{var}\begin{pmatrix}\boldsymbol{\delta}_x \\ \boldsymbol{\delta}_y\end{pmatrix}, \quad (5)$$

provided that $\boldsymbol{I}-\boldsymbol{B}$ is invertible. Let $\boldsymbol{\sigma}$ be the vector of all unique elements in (5) and $\boldsymbol{\beta}$ be the vector of free parameters in $\boldsymbol{\Lambda}_x$, $\boldsymbol{\Lambda}_y$, $\boldsymbol{B}$, $\boldsymbol{\Gamma}$, var($\boldsymbol{\xi}$), var($\boldsymbol{\delta}_x$), var($\boldsymbol{\delta}_y$), cov($\boldsymbol{\delta}_x$, $\boldsymbol{\delta}_y^T$), and var($\boldsymbol{\varepsilon}$). In order to link (5) to Assumption A1, we need the following mild assumptions on the covariance matrix (5).

A5 The covariance matrix (5) evaluated at $\boldsymbol{\beta}_0$ is positive definite. $\boldsymbol{\sigma}\left(\boldsymbol{\beta}\right)$ is a twice continuously differentiable function of $\boldsymbol{\beta}$ on a neighborhood of $\boldsymbol{\beta}_0$. The partial derivatives $\partial\boldsymbol{\sigma}\left(\boldsymbol{\beta}_0\right)/\partial\boldsymbol{\beta}$ and $\partial^2\boldsymbol{\sigma}\left(\boldsymbol{\beta}_0\right)/\partial\boldsymbol{\beta}\partial\boldsymbol{\beta}^T$ are finite.

By positive definiteness and the continuity in Assumption A5, we can find open balls $O^{(\theta)}$ and $O^{(\gamma)}$ such that $\boldsymbol{\theta}_0$ is an interior point of $O^{(\theta)}$, $\boldsymbol{\gamma}_0$ is an interior point of $O^{(\gamma)}$; (5) is positive definite for all $(\boldsymbol{\theta}, \boldsymbol{\gamma}) \in O^{(\theta)} \times O^{(\gamma)}$; and $\boldsymbol{\sigma}\left(\boldsymbol{\theta}, \boldsymbol{\gamma}\right)$, $\partial\boldsymbol{\sigma}\left(\boldsymbol{\beta}\right)/\partial\boldsymbol{\beta}$ and $\partial^2\boldsymbol{\sigma}\left(\boldsymbol{\beta}\right)/\partial\boldsymbol{\beta}\partial\boldsymbol{\beta}^T$ are bounded on $O^{(\theta)} \times O^{(\gamma)}$. If the structural equation model in the main text is the true

data generation process, then $\boldsymbol{\rho} = \boldsymbol{\sigma}(\boldsymbol{\theta}, \boldsymbol{\gamma})$. By the continuity, we can let $O^{(\rho)}$ be an open ball that is nested in the image of $O^{(\theta)} \times O^{(\gamma)}$ under $\boldsymbol{\sigma}(\boldsymbol{\theta}, \boldsymbol{\gamma})$, and then Result 1 holds. Further, Assumption A5 implies that $\partial \boldsymbol{\sigma}(\boldsymbol{\theta}_0, \boldsymbol{\gamma}) / \partial \boldsymbol{\gamma}$ and $\partial^2 \boldsymbol{\sigma}(\boldsymbol{\theta}_0, \boldsymbol{\gamma}) / \partial \boldsymbol{\gamma} \partial \boldsymbol{\gamma}^T$ are continuous functions of $\boldsymbol{\gamma} \in O^{(\gamma)}$. For a sufficiently large n, $\boldsymbol{\gamma}_0 + \boldsymbol{\delta}/\sqrt{n}$ is also an interior point of $O^{(\gamma)}$. Then, the Taylor's theorem (Rudin, 1976, pp 110 and pp 243) yields

$$\boldsymbol{\sigma}\left(\boldsymbol{\theta}_0, \boldsymbol{\gamma}_0 + \boldsymbol{\delta}/\sqrt{n}\right) = \boldsymbol{\sigma}(\boldsymbol{\theta}_0, \boldsymbol{\gamma}_0) + \frac{\partial \boldsymbol{\sigma}(\boldsymbol{\theta}_0, \boldsymbol{\gamma}_0)}{\partial \boldsymbol{r}^T} \boldsymbol{\delta}/\sqrt{n} + \frac{1}{2} \boldsymbol{\delta}^T \frac{\partial^2 \boldsymbol{\sigma}(\boldsymbol{\theta}_0, \tilde{\boldsymbol{\gamma}})}{\partial \boldsymbol{\gamma} \partial \boldsymbol{\gamma}^T} \boldsymbol{\delta}/n, \qquad (6)$$

where $\tilde{\boldsymbol{\gamma}}$ lies between $\boldsymbol{\gamma}_0 + \boldsymbol{\delta}/\sqrt{n}$ and $\boldsymbol{\gamma}_0$. Since $\partial \boldsymbol{\sigma}(\boldsymbol{\theta}_0, \boldsymbol{\gamma}) / \partial \boldsymbol{\gamma}$ and $\partial^2 \boldsymbol{\sigma}(\boldsymbol{\theta}_0, \boldsymbol{\gamma}) / \partial \boldsymbol{\gamma} \partial \boldsymbol{\gamma}^T$ are bounded on $O^{(\gamma)}$, the third term in the right-hand side of (6) is $O(n^{-1})$. Thus, Assumption A1 holds under Assumption A5.

Using Assumption A1 and Result 1, we can expand $\pi^{(ghst)}_{a_g a_h a_s a_t}(\boldsymbol{\tau}_{ghst}, \boldsymbol{\rho}_{ghst})$ that will be used later. For notational simplicity, we will drop the indices from $\pi^{(ghst)}_{a_g a_h a_s a_t}(\boldsymbol{\tau}_{ghst}, \boldsymbol{\rho}_{ghst})$ and express it as $\pi(\boldsymbol{\tau}, \boldsymbol{\rho})$ in this subsection, whenever no confusion arises. The partial derivatives shown in Result 1 imply that $\log \pi(\boldsymbol{\tau}, \boldsymbol{\rho})$ has continuous second-order partial derivatives on $O^{(\tau)} \times O^{(\rho)}$. Because of Assumption A1, $\boldsymbol{\rho}_{true}$ is an interior point of $O^{(\rho)}$ for a sufficiently large n. Hence, the Taylor's theorem (e.g., Rudin, 1976, pp 110 and pp 243) yields

$$\begin{aligned}
\log \pi(\boldsymbol{\tau}_0, \boldsymbol{\rho}_{true}) &= \log \pi(\boldsymbol{\tau}_0, \boldsymbol{\rho}_0) + \frac{\partial \log \pi(\boldsymbol{\tau}_0, \boldsymbol{\rho}_0)}{\partial \boldsymbol{\rho}^T} (\boldsymbol{\rho}_{true} - \boldsymbol{\rho}_0) \\
&\quad + \frac{1}{2} (\boldsymbol{\rho}_{true} - \boldsymbol{\rho}_0)^T \frac{\partial^2 \log \pi(\boldsymbol{\tau}_0, \tilde{\boldsymbol{\rho}})}{\partial \boldsymbol{\rho} \partial \boldsymbol{\rho}^T} (\boldsymbol{\rho}_{true} - \boldsymbol{\rho}_0) \\
&= \log \pi(\boldsymbol{\tau}_0, \boldsymbol{\rho}_0) + \frac{\partial \log \pi(\boldsymbol{\tau}_0, \boldsymbol{\rho}_0)}{\partial \boldsymbol{\rho}^T} \left(\boldsymbol{\delta}^*/\sqrt{n} + O\left(n^{-1}\right)\right) \\
&\quad + \frac{1}{2} \left(\boldsymbol{\delta}^*/\sqrt{n} + O\left(n^{-1}\right)\right)^T \frac{\partial^2 \log \pi(\boldsymbol{\tau}_0, \tilde{\boldsymbol{\rho}})}{\partial \boldsymbol{\rho} \partial \boldsymbol{\rho}^T} \left(\boldsymbol{\delta}^*/\sqrt{n} + O\left(n^{-1}\right)\right) \\
&= \log \pi(\boldsymbol{\tau}_0, \boldsymbol{\rho}_0) + \frac{\partial \log \pi(\boldsymbol{\tau}_0, \boldsymbol{\rho}_0)}{\partial \boldsymbol{\rho}^T} \boldsymbol{\delta}^*/\sqrt{n} + O\left(n^{-1}\right), \qquad (7)
\end{aligned}$$

where $\boldsymbol{\rho}_0$ is the vector that collects all $\rho_{gh,0}$, and $\tilde{\boldsymbol{\rho}}$ lies between $\boldsymbol{\rho}_0$, and $\boldsymbol{\rho}_{true}$ and $\boldsymbol{\delta}^*$ is the vector that collects all δ^*_{gh}. The remainder in (7) is $O(n^{-1})$ since the partial derivatives are bounded on $O^{(\tau)} \times O^{(\rho)}$ by Result 1 such that $\frac{\partial \log \pi(\boldsymbol{\tau}_0, \boldsymbol{\rho}_0)}{\partial \boldsymbol{\rho}^T} O(n^{-1}) = O(n^{-1})$ and

$\frac{\partial^2 \log \pi(\boldsymbol{\tau}_0, \tilde{\boldsymbol{\rho}})}{\partial \boldsymbol{\rho} \partial \boldsymbol{\rho}^T} \boldsymbol{\delta}^* / \sqrt{n} = O\left(n^{-1/2}\right)$. By taking $\exp()$ on both sides of (7), the expansion of $\log \pi\left(\boldsymbol{\tau}_0, \boldsymbol{\rho}_{true}\right)$ also gives the representation

$$\pi\left(\boldsymbol{\tau}_0, \boldsymbol{\rho}_{true}\right) = \pi\left(\boldsymbol{\tau}_0, \boldsymbol{\rho}_0\right) \exp\left\{\frac{\partial \log \pi\left(\boldsymbol{\tau}_0, \boldsymbol{\rho}_0\right)}{\partial \boldsymbol{\rho}^T} \boldsymbol{\delta}^* / \sqrt{n} + O\left(n^{-1}\right)\right\}.$$

Using the Taylor series $\exp(x) = 1 + x + \exp(\tilde{x}) x^2/2$ for some $0 < \tilde{x} < x$, we further obtain

$$\pi\left(\boldsymbol{\tau}_0, \boldsymbol{\rho}_{true}\right) = \pi\left(\boldsymbol{\tau}_0, \boldsymbol{\rho}_0\right)\left\{1 + \frac{\partial \log \pi\left(\boldsymbol{\tau}_0, \boldsymbol{\rho}_0\right)}{\partial \boldsymbol{\rho}^T} \boldsymbol{\delta}^* / \sqrt{n} + R\left(\boldsymbol{\delta}^*\right)\right\}, \quad (8)$$

where the non-random remainder

$$R\left(\boldsymbol{\delta}^*\right) = O\left(n^{-1}\right) + \frac{\exp(\tilde{x})}{2}\left[\frac{\partial \log \pi\left(\boldsymbol{\tau}_0, \boldsymbol{\rho}_0\right)}{\partial \boldsymbol{\rho}^T} \boldsymbol{\delta}^* / \sqrt{n} + O\left(n^{-1}\right)\right]^2$$

and $\tilde{x}$ lies between 0 and $\frac{\partial \log \pi(\boldsymbol{\tau}_0, \boldsymbol{\rho}_0)}{\partial \boldsymbol{\rho}^T} \boldsymbol{\delta}^* / \sqrt{n} + O\left(n^{-1}\right)$. Since the partial derivatives are bounded by Result 1, $\frac{\partial \log \pi(\boldsymbol{\tau}_0, \boldsymbol{\rho}_0)}{\partial \boldsymbol{\rho}^T} \boldsymbol{\delta}^* / \sqrt{n} = O\left(n^{-1/2}\right)$ and $\tilde{x} = O\left(n^{-1/2}\right)$. Hence, $R\left(\boldsymbol{\delta}^*\right) = O\left(n^{-1}\right)$.

When the dimension is two, similar steps yield

$$\begin{aligned} \pi_{ab}^{(gh)}\left(\boldsymbol{\tau}_{gh,0}, \rho_{gh,true}\right) &= \pi_{ab}^{(gh)}\left(\boldsymbol{\tau}_{gh,0}, \rho_{gh,0}\right) \exp\left\{\frac{\partial \log \pi_{ab}^{(gh)}\left(\boldsymbol{\tau}_{gh,0}, \rho_{gh,0}\right)}{\partial \rho} \delta_{gh}^* / \sqrt{n} + O\left(n^{-1}\right)\right\} \\ &= \pi_{ab}^{(gh)}\left(\boldsymbol{\tau}_{gh,0}, \rho_{gh,0}\right)\left[1 + \frac{\partial \log \pi_{ab}^{(gh)}\left(\boldsymbol{\tau}_{gh,0}, \rho_{gh,0}\right)}{\partial \rho} \delta_{gh}^* / \sqrt{n} + R_{ab}\left(\delta_{gh}^*\right)\right], \quad (9) \end{aligned}$$

for some non-random remainder $R_{ab}\left(\delta_{gh}^*\right) = O\left(n^{-1}\right)$.

2.4 Consistency and Asymptotic Normality

Throughout the paper, we use $E_{true}()$ to denote the expectation under the true distribution and $E_0()$ to denote the expectation under the distribution with ρ_0 as the parameter value. For example,

$$\begin{aligned} E_0\left[\ell_{gh,i}\left(\boldsymbol{\tau}_{gh,0}, \rho_{gh}\right)\right] &= \sum_{a=1}^{m_g} \sum_{b=1}^{m_h} \pi_{ab}\left(\boldsymbol{\tau}_{gh,0}, \rho_{gh,0}\right) \log \pi_{ab}\left(\boldsymbol{\tau}_{gh,0}, \rho_{gh}\right), \\ E_{true}\left[\ell_{gh,i}\left(\boldsymbol{\tau}_{gh,0}, \rho_{gh}\right)\right] &= \sum_{a=1}^{m_g} \sum_{b=1}^{m_h} \pi_{ab}\left(\boldsymbol{\tau}_{gh,0}, \rho_{gh,true}\right) \log \pi_{ab}\left(\boldsymbol{\tau}_{gh,0}, \rho_{gh}\right). \end{aligned}$$

The variances $\text{var}_{true}()$ and $\text{var}_0()$ are defined similarly.

When the two-step procedure is used to estimate the polychoric correlation coefficient, the thresholds $\boldsymbol{\tau}_g$ are estimated from the univariate standard normal distributions. Hence, $\hat{\boldsymbol{\tau}}_g$ is a still consistent estimator of $\boldsymbol{\tau}_g$ and $\sqrt{n}\left(\hat{\boldsymbol{\tau}}_g - \boldsymbol{\tau}_g\right)$ is asymptotically normal, for any g. In the second step, $\ell_{gh}\left(\hat{\boldsymbol{\tau}}_{gh}, \rho_{gh}\right)$ is maximized with respect to ρ_{gh}, a pseudo-maximum likelihood setting. Hence, we will extend Gong and Samaniego (1981) from the standard asymptotic framework to the local asymptotic framework.

Before we present the main results and their proofs, two well-known theorems are presented here, which will be repeatedly used.

1. Law of Large Numbers (LLN; e.g., Jiang, 2010, Theorem 6.4). Let $X_{n,i}$, $i = 1, ..., n$, $n = 1, 2, ...$, be a triangular array of random variables such that for each n, the $X_{n,i}$'s are independent. Then, $\sum_{i=1}^{n} X_{n,i} \xrightarrow{p} 0$ as $n \to \infty$ if and only if

$$\begin{aligned} \sum_{i=1}^{n} P\left(|X_{n,i}| > \epsilon\right) &\to 0, \\ \sum_{i=1}^{n} E\left\{X_{n,i} I\left(|X_{n,i}| \leq \kappa\right)\right\} &\to 0, \\ \sum_{i=1}^{n} \operatorname{var}\left\{X_{n,i} I\left(|X_{n,i}| \leq \kappa\right)\right\} &\to 0, \end{aligned}$$

for every $\epsilon > 0$ and some $\kappa > 0$.

2. Lindeberg-Feller Central Limit Theorem (e.g., van der Vaart, 1998, Proposition 2.27). For each n, let $Y_{n,1}$, ..., Y_{n,k_n}be independent random vectors with finite variances such that

$$\lim_{n\to\infty} \sum_{i=1}^{k_n} E\left[\|Y_{n,i}\|^2 I\left\{\|Y_{n,i}\| \geq \epsilon\right\}\right] = 0, \qquad \text{for every } \epsilon > 0$$

$$\sum_{i=1}^{k_n} \operatorname{cov}\left(Y_{n,i}\right) \to \Sigma.$$

Then the sequence $\sum_{i=1}^{k_n} \left[Y_{n,i} - E\left(Y_{n,i}\right)\right]$ converges in distribution to a normal $N\left(0, \Sigma\right)$ distribution.

Theorem 1. *Consider any pair* (g, h)*. For* $\varepsilon > 0$*, let* $A_n(\varepsilon)$ *be the event that, for any pairs* (g, h)*, there exists a root* $\hat{\rho}_{gh}$ *of the equation* $\partial \ell_{gh}\left(\hat{\boldsymbol{\tau}}_{gh}, \rho\right)/\partial\rho = 0$ *for which*

$|\hat{\rho}_{gh} - \rho_{gh,0}| < \varepsilon$. *Suppose that Assumptions* A1 *and* A2 *hold. Then for any* $\epsilon > 0$, $P\{A_n(\varepsilon)\} \to 1$ *as* $n \to \infty$, *under the local asymptotic framework. If Assumption* A3 *also holds, then* $\hat{\boldsymbol{\rho}} \xrightarrow{p} \boldsymbol{\rho}_0$, *under the local asymptotic framework.*

Proof of Theorem 1. The proof by and large follows Theorem 2.1 in Gong and Samaniego (1981), but to the local asymptotic framework. Let $A_{gh,n}(\varepsilon)$ be the event that there exists a root $\hat{\rho}_{gh}$ of the equation $\partial \ell_{gh}(\hat{\boldsymbol{\tau}}_{gh}, \rho)/\partial \rho = 0$ for which $|\hat{\rho}_{gh} - \rho_{gh,0}| < \varepsilon$. Suppose that, for any $\varepsilon_1 > 0$ and any $\varepsilon_2 > 0$, there exists a N_{gh} such that

$$P\{A_{gh,n}(\varepsilon_1)\} \;\; > \;\; 1 - 3\varepsilon_2, \tag{10}$$

for all $n > N_{gh}$. Let $N^* = \max_{(g,h)} N_{gh}$. Then,

$$P\{A_n(\varepsilon_1)\} \;\; = \;\; P\{A_{12,n}(\varepsilon_1) \cap A_{13,n}(\varepsilon_1) \cap \cdots \cap A_{q-1,q,n}(\varepsilon_1)\} > 1 - \frac{q(q-1)}{2} 3\varepsilon_2,$$

for all $n > N^*$. This means that $P\{A_n(\varepsilon_1)\} \to 1$ as $n \to \infty$. Further, under the assumption A3 that $\partial \ell_{gh}(\hat{\boldsymbol{\tau}}_{gh}, \rho)/\partial \rho = 0$ always has a unique root for any (g, h), we reach

$$P\left\{ \sqrt{\sum_{g<h} (\hat{\rho}_{gh} - \rho_{gh,0})^2} < \sqrt{\frac{q(q-1)}{2}} \varepsilon_1 \right\} \;\; > \;\; 1 - \frac{q(q-1)}{2} 3\varepsilon_2.$$

In other words, $\hat{\boldsymbol{\rho}} \xrightarrow{p} \boldsymbol{\rho}_0$. Thus, in the rest of the proof, we will show that (10) holds.

For notational simplicity, we drop the index g and h in the proof if no confusion arises. Let $O^{(\tau)}$ and $O^{(\rho)}$ be the open sets as stated in section 2.3. That is, $\boldsymbol{\tau}_0$ and ρ_0 are interior points of $O^{(\tau)}$ and $O^{(\rho)}$; and $\inf_{(\boldsymbol{\tau},\rho) \in O^{(\tau)} \times O^{(\rho)}} \pi_{ab}(\boldsymbol{\tau}, \rho) > 0$, for any a and b. By LLN,

$$\frac{1}{n} \ell(\boldsymbol{\tau}_0, \rho) - E_{true}[\ell_i(\boldsymbol{\tau}_0, \rho)] \;\; \xrightarrow{p} \;\; 0, \tag{11}$$

for any fixed $\rho \in O^{(\rho)}$, provided that

$$\sum_{i=1}^{n} P\left(|\ell_i(\boldsymbol{\tau}_0, \rho) - E_{true}[\ell_i(\boldsymbol{\tau}_0, \rho)]| > n\varepsilon\right) \;\; \to \;\; 0, \tag{12}$$

$$\sum_{i=1}^{n} E_{true}\left\{ \frac{\ell_i(\boldsymbol{\tau}_0, \rho) - E_{true}[\ell_i(\boldsymbol{\tau}_0, \rho)]}{n} I\left(|\ell_i(\boldsymbol{\tau}_0, \rho) - E_{true}[\ell_i(\boldsymbol{\tau}_0, \rho)]| \le n\kappa\right) \right\} \;\; \to \;\; 0, \tag{13}$$

$$\sum_{i=1}^{n} \mathrm{var}_{true}\left\{ \frac{\ell_i(\boldsymbol{\tau}_0, \rho) - E_{true}[\ell_i(\boldsymbol{\tau}_0, \rho)]}{n} I\left(|\ell_i(\boldsymbol{\tau}_0, \rho) - E_{true}[\ell_i(\boldsymbol{\tau}_0, \rho)]| \le n\kappa\right) \right\} \;\; \to \;\; 0, \tag{14}$$

for any $\varepsilon > 0$ and some $\kappa > 0$; and the expectations and variances are finite.

By Result 1, $\log \pi_{ab}(\boldsymbol{\tau}_0, \rho)$ is bounded on $O^{(\rho)}$ for any (a, b). Then, $\ell_i(\boldsymbol{\tau}_0, \rho)$,

$$\begin{aligned} E_0\left[\ell_i(\boldsymbol{\tau}_0, \rho)\right] &= \sum_{a,b} \pi_{ab}(\boldsymbol{\tau}_0, \rho_0) \log \pi_{ab}(\boldsymbol{\tau}_0, \rho), \\ E_0\left[\ell_i^2(\boldsymbol{\tau}_0, \rho)\right] &= \sum_{a,b} \pi_{ab}(\boldsymbol{\tau}_0, \rho_0) \left[\log \pi_{ab}(\boldsymbol{\tau}_0, \rho)\right]^2, \end{aligned}$$

and $\mathrm{var}_0(\ell_i(\boldsymbol{\tau}_0, \rho))$ are all bounded on $O^{(\rho)}$. By Assumption A1, equation (9) holds if $\rho_{true} \in O^{(\rho)}$. Hence, we have

$$\begin{aligned} E_{true}\left[\ell_i(\boldsymbol{\tau}_0, \rho)\right] &= \sum_{a,b} \pi_{ab}(\boldsymbol{\tau}_0, \rho_{true}) \log \pi_{ab}(\boldsymbol{\tau}_0, \rho) \\ &= E_0\left[\ell_i(\boldsymbol{\tau}_0, \rho)\right] + \sum_{a,b} \pi_{ab}(\boldsymbol{\tau}_0, \rho_0) \frac{\partial \log \pi_{ab}(\boldsymbol{\tau}_0, \rho_0)}{\partial \rho} \log \pi_{ab}(\boldsymbol{\tau}_0, \rho)\, \delta^*/\sqrt{n} \\ &\quad + \sum_{a,b} \pi_{ab}(\boldsymbol{\tau}_0, \rho_0) \log \pi_{ab}(\boldsymbol{\tau}_0, \rho)\, R_{ab}(\delta^*), \\ \mathrm{var}_{true}(\ell_i(\boldsymbol{\tau}_0, \rho)) &= E_{true}\left[\ell_i^2(\boldsymbol{\tau}_0, \rho)\right] - E_{true}^2\left[\ell_i(\boldsymbol{\tau}_0, \rho)\right] \\ &= E_0\left[\ell_i^2(\boldsymbol{\tau}_0, \rho)\right] + \sum_{a,b} \pi_{ab}(\boldsymbol{\tau}_0, \rho_0) \frac{\partial \log \pi_{ab}(\boldsymbol{\tau}_0, \rho_0)}{\partial \rho} \log^2 \pi_{ab}(\boldsymbol{\tau}_0, \rho)\, \delta^*/\sqrt{n} \\ &\quad + \sum_{a,b} \pi_{ab}(\boldsymbol{\tau}_0, \rho_0) \log^2 \pi_{ab}(\boldsymbol{\tau}_0, \rho)\, R_{ab}(\delta^*) - E_{true}^2\left[\ell_i(\boldsymbol{\tau}_0, \rho)\right]. \end{aligned}$$

By Result 1, $\sum_{a,b} \pi_{ab}(\boldsymbol{\tau}_0, \rho_0) \log \pi_{ab}(\boldsymbol{\tau}_0, \rho)$, $\sum_{a,b} \pi_{ab}(\boldsymbol{\tau}_0, \rho_0) \log^2 \pi_{ab}(\boldsymbol{\tau}_0, \rho)$,

$$\begin{aligned} &\sum_{a,b} \pi_{ab}(\boldsymbol{\tau}_0, \rho_0) \frac{\partial \log \pi_{ab}(\boldsymbol{\tau}_0, \rho_0)}{\partial \rho} \log \pi_{ab}(\boldsymbol{\tau}_0, \rho), \\ \text{and} \qquad &\sum_{a,b} \pi_{ab}(\boldsymbol{\tau}_0, \rho_0) \frac{\partial \log \pi_{ab}(\boldsymbol{\tau}_0, \rho_0)}{\partial \rho} \log^2 \pi_{ab}(\boldsymbol{\tau}_0, \rho), \end{aligned}$$

are all bounded on $O^{(\rho)}$. Since $R_{ab}(\delta^*) = O(n^{-1})$ in (9), we have

$$\begin{aligned} E_{true}(\ell_i(\boldsymbol{\tau}_0, \rho)) &= E_0\left[\ell_i(\boldsymbol{\tau}_0, \rho)\right] + O\left(n^{-1/2}\right), \\ \mathrm{var}_{true}(\ell_i(\boldsymbol{\tau}_0, \rho)) &= \mathrm{var}_0(\ell_i(\boldsymbol{\tau}_0, \rho)) + O\left(n^{-1/2}\right), \end{aligned}$$

and they are both finite for any fixed $\rho \in O^{(\rho)}$. Since both $\ell_i(\boldsymbol{\tau}_0, \rho)$ and $E_{true}\left[\ell_i(\boldsymbol{\tau}_0, \rho)\right]$ are bounded, $\left|\ell_i(\boldsymbol{\tau}_0, \rho) - E_{true}\left[\ell_i(\boldsymbol{\tau}_0, \rho)\right]\right|$ is bounded on $\rho \in O^{(\rho)}$. Hence, $\left|\ell_i(\boldsymbol{\tau}_0, \rho) - E_{true}\left[\ell_i(\boldsymbol{\tau}_0, \rho)\right]\right| < n\varepsilon$ for a sufficiently large n. Consequently, (12) holds.

Likewise, for a sufficiently large n, $|\ell_i(\boldsymbol{\tau}_0,\rho) - E_{true}[\ell_i(\boldsymbol{\tau}_0,\rho)]| \leq n\kappa$ for fixed κ. Thus, (13) and (14) hold, since

$$\sum_{i=1}^{n} E_{true}\left\{\frac{\ell_i(\boldsymbol{\tau}_0,\rho) - E_{true}[\ell_i(\boldsymbol{\tau}_0,\rho)]}{n}\right\} = 0$$

and

$$\begin{aligned}\sum_{i=1}^{n} \mathrm{var}_{true}\left\{\frac{\ell_i(\boldsymbol{\tau}_0,\rho) - E_{true}[\ell_i(\boldsymbol{\tau}_0,\rho)]}{n}\right\} &= \frac{\mathrm{var}_{true}\{\ell_i(\boldsymbol{\tau}_0,\rho)\}}{n} \\ &= \frac{\mathrm{var}_0(\ell_i(\boldsymbol{\tau}_0,\rho)) + O\left(n^{-1/2}\right)}{n} \to 0.\end{aligned}$$

Therefore, the law of large numbers (11) is applicable and

$$P\left(\left|\frac{1}{n}\ell(\boldsymbol{\tau}_0,\rho) - E_{true}[\ell_i(\boldsymbol{\tau}_0,\rho)]\right| > \epsilon\right) \to 0,$$

for any $\epsilon > 0$, as $n \to \infty$. It further yields

$$\frac{1}{n}\ell(\boldsymbol{\tau}_0,\rho) \xrightarrow{p} E_0[\ell_i(\boldsymbol{\tau}_0,\rho)],$$

since the local drift in the expectation vanishes as $n \to \infty$.

From section 2.3 we know that $\ell(\boldsymbol{\tau},\rho)$ has continuous partial derivatives with respect to $(\boldsymbol{\tau},\rho)$ on the open set $O^{(\tau)} \times O^{(\rho)}$. Hence, for a sufficiently large n, the consistency of $\hat{\boldsymbol{\tau}}$ and the Taylor's theorem yields

$$\frac{1}{n}\ell(\hat{\boldsymbol{\tau}},\rho) = \frac{1}{n}\ell(\boldsymbol{\tau}_0,\rho) + \frac{1}{n}\frac{\partial\ell(\tilde{\boldsymbol{\tau}},\rho)}{\partial\boldsymbol{\tau}^T}(\hat{\boldsymbol{\tau}} - \boldsymbol{\tau}_0) + o_{\mathrm{P}}(1),$$

where $\tilde{\boldsymbol{\tau}}$ lies between $\hat{\boldsymbol{\tau}}$ and $\boldsymbol{\tau}_0$. By Result 1, $|\partial\log\pi_{ab}(\boldsymbol{\tau},\rho)/\partial\boldsymbol{\tau}|$ is bounded on $O^{(\tau)} \times O^{(\rho)}$. Hence, for all $(\boldsymbol{\tau},\rho) \in O^{(\tau)} \times O^{(\rho)}$,

$$\left|\frac{\partial\ell_i(\boldsymbol{\tau},\rho)}{\partial\tau}\right| = \sum_{a,b}\left|I_i(a,b)\frac{\partial\log\pi_{ab}(\boldsymbol{\tau},\rho)}{\partial\tau}\right|$$

is also bounded. Since $\hat{\boldsymbol{\tau}}$ is still a consistent estimator of $\boldsymbol{\tau}_0$, $P\left\{(\hat{\boldsymbol{\tau}} - \boldsymbol{\tau}_0)^T(\hat{\boldsymbol{\tau}} - \boldsymbol{\tau}_0) < \epsilon\right\} \to 1$ as $n \to \infty$ for any $\epsilon > 0$. For a sufficiently small ϵ, the

open ball $\left\{\boldsymbol{\tau};\ (\hat{\boldsymbol{\tau}}-\boldsymbol{\tau}_0)^T(\hat{\boldsymbol{\tau}}-\boldsymbol{\tau}_0)<\epsilon\right\}$ is a subset of $O^{(\tau)}$. Hence, $P\left(\hat{\boldsymbol{\tau}}\in O^{(\tau)}\right)\to 1$. Equivalently,

$$P\left((\hat{\boldsymbol{\tau}},\rho)\in O^{(\tau)}\times O^{(\rho)}\right) \ \to\ 1$$

for any fixed $\rho\in O^{(\rho)}$, as $n\to\infty$. Then $n^{-1}\partial\ell(\tilde{\boldsymbol{\tau}},\rho)/\partial\boldsymbol{\tau}$ is bounded in probability. Hence,

$$P\left(\left|\frac{1}{n}\ell(\hat{\boldsymbol{\tau}},\rho)-\frac{1}{n}\ell(\boldsymbol{\tau}_0,\rho)\right|>\epsilon\right) \ \to\ 0.$$

Above all,

$$\frac{1}{n}\ell(\hat{\boldsymbol{\tau}},\rho)-E_{true}\left[\ell_i(\boldsymbol{\tau}_0,\rho)\right] \ \xrightarrow{p}\ 0, \tag{15}$$

for $\rho\in O^{(\rho)}$, since for any $\epsilon>0$,

$$\begin{aligned} P\left(\left|\frac{1}{n}\ell(\hat{\boldsymbol{\tau}},\rho)-E_{true}\left[\ell_i(\boldsymbol{\tau}_0,\rho)\right]\right|>2\epsilon\right) \ &\le\ P\left(\left|\frac{1}{n}\ell(\hat{\boldsymbol{\tau}},\rho)-\frac{1}{n}\ell(\boldsymbol{\tau}_0,\rho)\right|>\epsilon\right) \\ &\quad +P\left(\left|\frac{1}{n}\ell(\boldsymbol{\tau}_0,\rho)-E_{true}\left[\ell_i(\boldsymbol{\tau}_0,\rho)\right]\right|>\epsilon\right), \end{aligned}$$

which converges to 0 as $n\to\infty$. It further yields

$$\frac{1}{n}\ell(\hat{\boldsymbol{\tau}},\rho) \ \xrightarrow{p}\ E_0\left[\ell_i(\boldsymbol{\tau}_0,\rho)\right],$$

since the local drift in the expectation vanishes as $n\to\infty$.

The rest of the proof mimics the proof of Theorem 2.1 in Gong and Samaniego (1981). By (15),

$$\left[\frac{1}{n}\ell(\hat{\boldsymbol{\tau}},\rho)-\frac{1}{n}\ell(\hat{\boldsymbol{\tau}},\rho_0)\right]-\left\{E_0\left[\ell_i(\boldsymbol{\tau}_0,\rho)\right]-E_0\left[\ell_i(\boldsymbol{\tau}_0,\rho_0)\right]\right\} \ \xrightarrow{p}\ 0.$$

By Jensen's inequality

$$E_0\left[\ell_i(\boldsymbol{\tau}_0,\rho)\right]-E_0\left[\ell_i(\boldsymbol{\tau}_0,\rho_0)\right] \ \le\ \log E_0\frac{\prod_{a=1}^{m_x}\prod_{b=1}^{m_y}\left[\pi_{ab}(\boldsymbol{\tau}_0,\rho)\right]^{I_i(a,b)}}{\prod_{a=1}^{m_x}\prod_{b=1}^{m_y}\left[\pi_{ab}(\boldsymbol{\tau}_0,\rho_0)\right]^{I_i(a,b)}}=0.$$

By Assumption A2, the equality is attained only if $\rho=\rho_0$. Hence, $n^{-1}\left[\ell(\hat{\boldsymbol{\tau}},\rho)-\ell(\hat{\boldsymbol{\tau}},\rho_0)\right]$ converges in probability to a negative number for $\rho\neq\rho_0$. Consequently, for any small

$\varepsilon_1 > 0$ and $\varepsilon_2 > 0$, there exists a N such that

$$1 - \varepsilon_2 \;<\; P\left(\hat{\boldsymbol{\tau}} \in O^{(\tau)}\right), \tag{16}$$

$$1 - \varepsilon_2 \;<\; P\left(\ell\left(\hat{\boldsymbol{\tau}}, \rho_0 + \varepsilon_1\right) < \ell\left(\hat{\boldsymbol{\tau}}, \rho_0\right)\right), \tag{17}$$

$$1 - \varepsilon_2 \;<\; P\left(\ell\left(\hat{\boldsymbol{\tau}}, \rho_0 - \varepsilon_1\right) < \ell\left(\hat{\boldsymbol{\tau}}, \rho_0\right)\right), \tag{18}$$

for all $n > N$. Here (16) holds since $\hat{\boldsymbol{\tau}}$ is a consistent estimator of $\boldsymbol{\tau}_0$; (17) and (18) hold because of the convergence of $n^{-1}\left[\ell\left(\hat{\boldsymbol{\tau}}, \rho\right) - \ell\left(\hat{\boldsymbol{\tau}}, \rho_0\right)\right]$ that we just showed. Suppose that the event

$$\left\{\hat{\boldsymbol{\tau}} \in O^{(\tau)},\ \ell\left(\hat{\boldsymbol{\tau}}, \rho_0 + \varepsilon_1\right) < \ell\left(\hat{\boldsymbol{\tau}}, \rho_0\right),\ \text{and}\ \ell\left(\hat{\boldsymbol{\tau}}, \rho_0 - \varepsilon_1\right) < \ell\left(\hat{\boldsymbol{\tau}}, \rho_0\right)\right\} \tag{19}$$

holds. Note that, for a given $\hat{\boldsymbol{\tau}}$, $\ell\left(\hat{\boldsymbol{\tau}}, \rho\right)$ is a continuous function of ρ on the closed and bounded interval $[\rho_0 - \varepsilon_1, \rho_0 + \varepsilon_1]$. By the extreme value theorem (Rudin, 1976, Theorem 4.16), $\ell\left(\hat{\boldsymbol{\tau}}, \rho\right)$ must attain a maximum. Since the values of $\ell\left(\hat{\boldsymbol{\tau}}, \rho\right)$ at boundaries are lower than the value at an interior point ($\rho = \rho_0$) by event (19), the maximum of $\ell\left(\hat{\boldsymbol{\tau}}, \rho\right)$ on the closed interval $[\rho_0 - \varepsilon_1, \rho_0 + \varepsilon_1]$ must be attained at an interior point. This means that, if event (19) holds, $\ell\left(\hat{\boldsymbol{\tau}}, \rho\right)$ has a local maximum $\hat{\rho}$ on the open interval $(\rho_0 - \varepsilon_1, \rho_0 + \varepsilon_1)$. Since $\ell\left(\hat{\boldsymbol{\tau}}, \rho\right)$ is differentiable with respect to ρ, the local maximum $\hat{\rho}$ satisfies $\partial\ell\left(\hat{\boldsymbol{\tau}}, \rho\right)/\partial\rho = 0$. By inequalities (16), (17), and (18), the event (19) holds with probability greater than $1 - 3\varepsilon_2$, which completes the proof of (10).

□

Theorem 2. *Suppose that Assumptions* A1*,* A2*,* A3*, and* A4 *hold. Then* $\sqrt{n}\left(\hat{\boldsymbol{\rho}} - \boldsymbol{\rho}_{true}\right) \xrightarrow{d} N\left(\mathbf{0}, \boldsymbol{\Upsilon}\right)$*, under the local asymptotic framework, where* $\boldsymbol{\Upsilon}$ *is the same as the asymptotic covariance matrix in* (2)*.*

Proof of Theorem 2. For ease of presentation, we first prove the asymptotic normality of the polychoric correlation coefficient estimator between two variables. Second, we extend the proof to the multivariate case. Since the univariate distribution is not locally drifted, the expansion for the thresholds in Jöreskog (1994) is still applicable. However, ρ is locally

drifted by Assumption A1. Hence, the expansion for ρ in Jöreskog (1994) is not directly applicable.

The marginal probabilities of observing $x_g = a$ and $x_h = b$ are

$$\pi_{g,a}(\boldsymbol{\tau}_g) = \int_{\tau_{g,a-1}}^{\tau_{g,a}} \phi_1(t)\,dt, \qquad \pi_{h,b}(\boldsymbol{\tau}_h) = \int_{\tau_{h,b-1}}^{\tau_{h,b}} \phi_1(t)\,dt,$$

respectively, where $\phi_1()$ is the density function of a standard normal random variable. To estimate $\boldsymbol{\tau}_g$, the univariate log-likelihood scaled by n^{-1} is

$$n^{-1}\sum_{i=1}^{n}\sum_{a=1}^{m_g} I\{x_{g,i} = a\}\log \pi_{g,a}(\boldsymbol{\tau}_g), \tag{20}$$

where $\tau_{g,0} \equiv -\infty$ and $\tau_{g,m_g} \equiv \infty$. Denote the observed relative frequency matrix by $\boldsymbol{P}^{(gh)}$ $(m_g \times m_h)$. Let the operator diag$(\cdot)$ construct a diagonal matrix using the enclosed vector as diagonal elements. Jöreskog (1994) showed that

$$\sqrt{n}\left(\hat{\boldsymbol{\tau}}_g - \boldsymbol{\tau}_{g,0}\right) = \left(\boldsymbol{B}_{g,0}^T \boldsymbol{D}_{g,0}^{-1}\boldsymbol{B}_{g,0}\right)^{-1}\boldsymbol{B}_{g,0}^T\boldsymbol{D}_{g,0}^{-1}\sqrt{n}\boldsymbol{P}^{(gh)}\mathbf{1}_g + o_{\mathrm{P}}(1), \tag{21}$$

where $\boldsymbol{B}_{g,0} = \boldsymbol{B}_g(\boldsymbol{\tau}_{g,0})$ with

$$\boldsymbol{B}_g(\boldsymbol{\tau}_g) = \begin{pmatrix} \phi_1(\tau_{g,1}) & 0 & \cdots & 0 \\ -\phi_1(\tau_{g,1}) & \phi_1(\tau_{g,2}) & \cdots & 0 \\ 0 & -\phi_1(\tau_{g,2}) & \cdots & 0 \\ \vdots & \vdots & \ddots & \vdots \\ 0 & 0 & \cdots & \phi_1\left(\tau_{g,m_g-1}\right) \\ 0 & 0 & \cdots & -\phi_1\left(\tau_{g,m_g-1}\right) \end{pmatrix},$$

$\boldsymbol{D}_{g,0} = \boldsymbol{D}_g(\boldsymbol{\pi}_{g,0})$ with $\boldsymbol{D}_g(\boldsymbol{\pi}_g) = \mathrm{diag}(\boldsymbol{\pi}_g)$ and $\boldsymbol{\pi}_g = \begin{pmatrix}\pi_{g,1} & \cdots & \pi_{g,m_g}\end{pmatrix}^T$, and $\mathbf{1}_g$ is a $m_h \times 1$ column vector of ones. It is worth mentioning that $\sqrt{n}\boldsymbol{P}^{(gh)}\mathbf{1}_g$ in (21) diverges as $n \to \infty$, but $\boldsymbol{B}_{g,0}^T\boldsymbol{D}_{g,0}^{-1}\sqrt{n}\boldsymbol{P}^{(gh)}\mathbf{1}_g$ does not. Let $\boldsymbol{\pi}_0^{(gh)}$ be the $m_g \times m_h$ matrix with entries $\pi_{ab}^{(gh)}(\boldsymbol{\tau}_{gh,0}, \rho_{gh,0})$. It is easy to see that $\boldsymbol{D}_{g,0}^{-1}\boldsymbol{\pi}_0^{(gh)}\mathbf{1}_g = \mathbf{1}_g$ and $\boldsymbol{B}_{g,0}^T\boldsymbol{D}_{g,0}^{-1}\boldsymbol{\pi}_0^{(gh)}\mathbf{1}_g = \boldsymbol{B}_{g,0}^T\mathbf{1}_g = \mathbf{0}$. Hence, we get $\boldsymbol{B}_{g,0}^T\boldsymbol{D}_{g,0}^{-1}\sqrt{n}\boldsymbol{P}^{(gh)}\mathbf{1}_g = \boldsymbol{B}_{g,0}^T\boldsymbol{D}_{g,0}^{-1}\sqrt{n}\left(\boldsymbol{P}^{(gh)} - \boldsymbol{\pi}_0^{(gh)}\right)\mathbf{1}_g$. Then, an equivalent

expression of (21) is

$$\sqrt{n}\left(\hat{\boldsymbol{\tau}}_g-\boldsymbol{\tau}_{g,0}\right)=\left(\boldsymbol{B}_{g,0}^T\boldsymbol{D}_{g,0}^{-1}\boldsymbol{B}_{g,0}\right)^{-1}\boldsymbol{B}_{g,0}^T\boldsymbol{D}_{g,0}^{-1}\sqrt{n}\left(\boldsymbol{P}^{(gh)}-\boldsymbol{\pi}_0^{(gh)}\right)\mathbf{1}_g+o_{\mathrm{P}}\left(1\right). \tag{22}$$

In fact, $\boldsymbol{B}_{g,0}^T\boldsymbol{D}_{g,0}^{-1}\sqrt{n}\boldsymbol{P}^{(gh)}\mathbf{1}_g$ originates from the first-order derivative of (20) given by a vector with entries

$$\left(\frac{n^{-1}\sum_{i=1}^n I\left\{x_{g,i}=a\right\}}{\pi_{g,a}\left(\boldsymbol{\tau}_g\right)}-\frac{n^{-1}\sum_{i=1}^n I\left\{x_{g,i}=a+1\right\}}{\pi_{g,a+1}\left(\boldsymbol{\tau}_g\right)}\right)\phi_1\left(\tau_{g,a}\right).$$

Likewise, $\hat{\boldsymbol{\tau}}_h$ satisfies

$$\sqrt{n}\left(\hat{\boldsymbol{\tau}}_h-\boldsymbol{\tau}_{h,0}\right)=\left(\boldsymbol{B}_{h,0}^T\boldsymbol{D}_{h,0}^{-1}\boldsymbol{B}_{h,0}\right)^{-1}\boldsymbol{B}_{h,0}^T\boldsymbol{D}_{h,0}^{-1}\sqrt{n}\left(\boldsymbol{P}^{(gh)}-\boldsymbol{\pi}_0^{(gh)}\right)^T\mathbf{1}_h+o_{\mathrm{P}}\left(1\right). \tag{23}$$

For the asymptotic normality of the polychoric correlation coefficient estimator between two variables, we often suppress the index g and h for notational simplicity if it does not cause confusion. From section 2.3, it can be easily seen that $\ell\left(\boldsymbol{\tau},\rho\right)$ has continuous third-order partial derivatives with respect to $(\boldsymbol{\tau},\rho)$ on the open set $O^{(\tau)}\times O^{(\rho)}$. Hence, the Taylor's theorem yields

$$\begin{aligned}
0 &= \frac{1}{\sqrt{n}}\frac{\partial\ell\left(\hat{\boldsymbol{\tau}},\hat{\rho}\right)}{\partial\rho}\\
&= \left[\frac{1}{n}\frac{\partial^2\ell\left(\boldsymbol{\tau}_0,\rho_0\right)}{\partial\rho^2}+\frac{1}{2n}\frac{\partial^3\ell\left(\tilde{\boldsymbol{\tau}},\tilde{\rho}\right)}{\partial\rho^3}\left(\hat{\rho}-\rho_0\right)+\frac{1}{n}\frac{\partial^3\ell\left(\tilde{\boldsymbol{\tau}},\tilde{\rho}\right)}{\partial\rho^2\partial\boldsymbol{\tau}^T}\left(\hat{\boldsymbol{\tau}}-\boldsymbol{\tau}_0\right)\right]\sqrt{n}\left(\hat{\rho}-\rho_0\right)\\
&\quad+\frac{1}{\sqrt{n}}\frac{\partial\ell\left(\boldsymbol{\tau}_0,\rho_0\right)}{\partial\rho}+\frac{1}{n}\frac{\partial^2\ell\left(\boldsymbol{\tau}_0,\rho_0\right)}{\partial\rho\partial\boldsymbol{\tau}^T}\sqrt{n}\left(\hat{\boldsymbol{\tau}}-\boldsymbol{\tau}_0\right)+\frac{1}{2\sqrt{n}}\left(\hat{\boldsymbol{\tau}}-\boldsymbol{\tau}_0\right)^T\frac{\partial^2\ell\left(\tilde{\boldsymbol{\tau}},\tilde{\rho}\right)}{\partial\rho\partial\boldsymbol{\tau}\partial\boldsymbol{\tau}^T}\left(\hat{\boldsymbol{\tau}}-\boldsymbol{\tau}_0\right),
\end{aligned}$$

where $\tilde{\rho}$ lies between $\hat{\rho}$ and ρ_0, and $\tilde{\boldsymbol{\tau}}$ lies between $\hat{\boldsymbol{\tau}}$ and $\boldsymbol{\tau}_0$. Consequently,

$$\sqrt{n}\left(\hat{\rho}-\rho_0\right) = -\frac{\frac{1}{\sqrt{n}}\frac{\partial\ell(\boldsymbol{\tau}_0,\rho_0)}{\partial\rho}+\frac{1}{n}\frac{\partial^2\ell(\boldsymbol{\tau}_0,\rho_0)}{\partial\rho\partial\boldsymbol{\tau}^T}\sqrt{n}\left(\hat{\boldsymbol{\tau}}-\boldsymbol{\tau}_0\right)+\frac{1}{2\sqrt{n}}\left(\hat{\boldsymbol{\tau}}-\boldsymbol{\tau}_0\right)^T\frac{\partial^2\ell(\tilde{\boldsymbol{\tau}},\tilde{\rho})}{\partial\rho\partial\boldsymbol{\tau}\partial\boldsymbol{\tau}^T}\left(\hat{\boldsymbol{\tau}}-\boldsymbol{\tau}_0\right)}{\frac{1}{n}\frac{\partial^2\ell(\boldsymbol{\tau}_0,\rho_0)}{\partial\rho^2}+\frac{1}{2n}\frac{\partial^3\ell(\tilde{\boldsymbol{\tau}},\tilde{\rho})}{\partial\rho^3}\left(\hat{\rho}-\rho_0\right)+\frac{1}{n}\frac{\partial^3\ell(\tilde{\boldsymbol{\tau}},\tilde{\rho})}{\partial\rho^2\partial\boldsymbol{\tau}^T}\left(\hat{\boldsymbol{\tau}}-\boldsymbol{\tau}_0\right)}, \tag{24}$$

provided that the denominator is nonzero. We will show later that the denominator converges in probability to a nonzero constant. Hence, with probability approaching 1, the denominator is nonzero.

We first consider the numerator of (24). From equations (22) and (23), we have

$$\begin{aligned}
&\frac{1}{\sqrt{n}}\frac{\partial \ell\left(\boldsymbol{\tau}_0,\rho_0\right)}{\partial \rho}+\left[\frac{1}{n}\frac{\partial^2 \ell\left(\boldsymbol{\tau}_0,\rho_0\right)}{\partial \rho \partial \boldsymbol{\tau}^T}\right]\sqrt{n}\left(\hat{\boldsymbol{\tau}}-\boldsymbol{\tau}_0\right)\\
= &\frac{1}{\sqrt{n}}\frac{\partial \ell\left(\boldsymbol{\tau}_0,\rho_0\right)}{\partial \rho}+\left[\frac{1}{n}\frac{\partial^2 \ell\left(\boldsymbol{\tau}_0,\rho_0\right)}{\partial \rho \partial \boldsymbol{\tau}^T}\right]\begin{pmatrix}\left(\boldsymbol{B}_{g,0}^T\boldsymbol{D}_{g,0}^{-1}\boldsymbol{B}_{g,0}\right)^{-1}\boldsymbol{B}_{g,0}^T\boldsymbol{D}_{g,0}^{-1}\sqrt{n}\left(\boldsymbol{P}-\boldsymbol{\pi}_0\right)\mathbf{1}_g+o_{\mathrm{P}}(1)\\ \left(\boldsymbol{B}_{h,0}^T\boldsymbol{D}_{h,0}^{-1}\boldsymbol{B}_{h,0}\right)^{-1}\boldsymbol{B}_{h,0}^T\boldsymbol{D}_{h,0}^{-1}\sqrt{n}\left(\boldsymbol{P}-\boldsymbol{\pi}_0\right)^T\mathbf{1}_h+o_{\mathrm{P}}(1)\end{pmatrix}\\
= &\sqrt{n}\sum_{a=1}^{m_g}\sum_{b=1}^{m_h}P_{ab}\frac{\partial \log \pi_{ab}\left(\boldsymbol{\tau}_0,\rho_0\right)}{\partial \rho}+\left[\frac{1}{n}\frac{\partial^2 \ell\left(\boldsymbol{\tau}_0,\rho_0\right)}{\partial \rho \partial \boldsymbol{\tau}_g^T}\right]\left(\boldsymbol{B}_{g,0}^T\boldsymbol{D}_{g,0}^{-1}\boldsymbol{B}_{g,0}\right)^{-1}\boldsymbol{B}_{g,0}^T\boldsymbol{D}_{g,0}^{-1}\sqrt{n}\left(\boldsymbol{P}-\boldsymbol{\pi}_0\right)\mathbf{1}_g\\
&+\left[\frac{1}{n}\frac{\partial^2 \ell\left(\boldsymbol{\tau}_0,\rho_0\right)}{\partial \rho \partial \boldsymbol{\tau}_h^T}\right]\left(\boldsymbol{B}_{h,0}^T\boldsymbol{D}_{h,0}^{-1}\boldsymbol{B}_{h,0}\right)^{-1}\boldsymbol{B}_{h,0}^T\boldsymbol{D}_{h,0}^{-1}\sqrt{n}\left(\boldsymbol{P}-\boldsymbol{\pi}_0\right)^T\mathbf{1}_h+\left[\frac{1}{n}\frac{\partial^2 \ell\left(\boldsymbol{\tau}_0,\rho_0\right)}{\partial \rho \partial \boldsymbol{\tau}^T}\right]o_{\mathrm{P}}(1)\\
= &\sqrt{n}\mathrm{tr}\left\{\boldsymbol{A}^T\boldsymbol{P}\right\}+\mathrm{tr}\left\{\mathbf{1}_g\left[\frac{1}{n}\frac{\partial^2 \ell\left(\boldsymbol{\tau}_0,\rho_0\right)}{\partial \rho \partial \boldsymbol{\tau}_g^T}\right]\left(\boldsymbol{B}_{g,0}^T\boldsymbol{D}_{g,0}^{-1}\boldsymbol{B}_{g,0}\right)^{-1}\boldsymbol{B}_{g,0}^T\boldsymbol{D}_{g,0}^{-1}\sqrt{n}\left(\boldsymbol{P}-\boldsymbol{\pi}_0\right)\right\}\\
&+\mathrm{tr}\left\{\boldsymbol{D}_{h,0}^{-1}\boldsymbol{B}_{h,0}\left(\boldsymbol{B}_{h,0}^T\boldsymbol{D}_{h,0}^{-1}\boldsymbol{B}_{h,0}\right)^{-1}\left[\frac{1}{n}\frac{\partial^2 \ell\left(\boldsymbol{\tau}_0,\rho_0\right)}{\partial \rho \partial \boldsymbol{\tau}_h}\right]\mathbf{1}_h^T\sqrt{n}\left(\boldsymbol{P}-\boldsymbol{\pi}_0\right)\right\}\\
&+\left[\frac{1}{n}\frac{\partial^2 \ell\left(\boldsymbol{\tau}_0,\rho_0\right)}{\partial \rho \partial \boldsymbol{\tau}^T}\right]o_{\mathrm{P}}(1), \qquad (25)
\end{aligned}$$

where P_{ab} is the (a,b)th entry of $\boldsymbol{P}$ and the (a,b)th entry of $\boldsymbol{A}$ is $\partial \log \pi_{ab}\left(\boldsymbol{\tau}_0,\rho_0\right)/\partial\rho$. Note that

$$\mathrm{tr}\left\{\boldsymbol{A}^T\boldsymbol{\pi}_0\right\} = \sum_{a=1}^{m_g}\sum_{b=1}^{m_h}\frac{\partial \log \pi_{ab}\left(\boldsymbol{\tau}_0,\rho_0\right)}{\partial \rho}\pi_{ab,0}=\sum_{a=1}^{m_g}\sum_{b=1}^{m_h}\frac{\partial \pi_{ab}\left(\boldsymbol{\tau}_0,\rho_0\right)}{\partial \rho}=0.$$

Then, we can express $\sqrt{n}\mathrm{tr}\left\{\boldsymbol{A}^T\boldsymbol{P}\right\}$ in (25) as $\sqrt{n}\mathrm{tr}\left\{\boldsymbol{A}^T\left(\boldsymbol{P}-\boldsymbol{\pi}_0\right)\right\}$. Further, by Result 1, $\partial^2 \log \pi_{ab}\left(\boldsymbol{\tau}_0,\rho_0\right)/\partial\rho\partial\boldsymbol{\tau}$ is bounded on $O^{(\tau)}\times O^{(\rho)}$. Hence

$$\frac{1}{n}\frac{\partial^2 \ell\left(\rho_0,\boldsymbol{\tau}_0\right)}{\partial \rho \partial \boldsymbol{\tau}^T} = \sum_{a=1}^{m_g}\sum_{b=1}^{m_h}P_{ab}\frac{\partial^2 \log \pi_{ab}\left(\boldsymbol{\tau}_0,\rho_0\right)}{\partial \rho \partial \boldsymbol{\tau}^T}$$

is also bounded. Consequently

$$\left[\frac{1}{n}\frac{\partial^2 \ell\left(\rho_0,\boldsymbol{\tau}_0\right)}{\partial \rho \partial \boldsymbol{\tau}^T}\right]o_{\mathrm{P}}(1)=o_{\mathrm{P}}(1).$$

As a result, (25) becomes

$$\frac{1}{\sqrt{n}}\frac{\partial \ell\left(\rho_0,\boldsymbol{\tau}_0\right)}{\partial \rho}+\left[\frac{1}{n}\frac{\partial^2 \ell\left(\rho_0,\boldsymbol{\tau}_0\right)}{\partial \rho \partial \boldsymbol{\tau}^T}\right]\sqrt{n}\left(\hat{\boldsymbol{\tau}}-\boldsymbol{\tau}_0\right) = \sqrt{n}\mathrm{tr}\left\{\boldsymbol{\Lambda}\left(\boldsymbol{P}-\boldsymbol{\pi}_0\right)\right\}+o_{\mathrm{P}}(1), \qquad (26)$$

with $\mathrm{tr}\left\{\boldsymbol{\Lambda}\boldsymbol{\pi}_0\right\}=\mathbf{0}$, where

$$\begin{aligned}
\boldsymbol{\Lambda} = &\boldsymbol{A}^T+\mathbf{1}_g\left[\frac{1}{n}\frac{\partial^2 \ell\left(\rho_0,\boldsymbol{\tau}_0\right)}{\partial \rho \partial \boldsymbol{\tau}_g^T}\right]\left(\boldsymbol{B}_{g,0}^T\boldsymbol{D}_{g,0}^{-1}\boldsymbol{B}_{g,0}\right)^{-1}\boldsymbol{B}_{g,0}^T\boldsymbol{D}_{g,0}^{-1}\\
&+\boldsymbol{D}_{h,0}^{-1}\boldsymbol{B}_{h,0}\left(\boldsymbol{B}_{h,0}^T\boldsymbol{D}_{h,0}^{-1}\boldsymbol{B}_{h,0}\right)^{-1}\left[\frac{1}{n}\frac{\partial^2 \ell\left(\rho_0,\boldsymbol{\tau}_0\right)}{\partial \rho \partial \boldsymbol{\tau}_h}\right]\mathbf{1}_h^T.
\end{aligned}$$

By LLN,

$$\frac{1}{n}\frac{\partial^2 \ell\left(\rho_0, \boldsymbol{\tau}_0\right)}{\partial \rho^2} - E_{true}\left(\frac{\partial^2 \ell_i\left(\boldsymbol{\tau}_0, \rho_0\right)}{\partial \rho^2}\right) \overset{p}{\to} 0, \tag{27}$$

if and only if

$$\sum_{i=1}^{n} P\left(\left|\frac{\partial^2 \ell_i\left(\rho_0, \boldsymbol{\tau}_0\right)}{\partial \rho^2} - E_{true}\left(\frac{\partial^2 \ell_i\left(\boldsymbol{\tau}_0, \rho_0\right)}{\partial \rho^2}\right)\right| > n\epsilon\right) \to 0,$$

$$\sum_{i=1}^{n} E_{true}\left\{\frac{\frac{\partial^2 \ell_i(\rho_0, \boldsymbol{\tau}_0)}{\partial \rho^2} - E_{true}\left(\frac{\partial^2 \ell_i(\boldsymbol{\tau}_0, \rho_0)}{\partial \rho^2}\right)}{n} I\left(\left|\frac{\partial^2 \ell_i\left(\rho_0, \boldsymbol{\tau}_0\right)}{\partial \rho^2} - E_{true}\left(\frac{\partial^2 \ell_i\left(\boldsymbol{\tau}_0, \rho_0\right)}{\partial \rho^2}\right)\right| \leq n\kappa\right)\right\} \to 0,$$

$$\sum_{i=1}^{n} \mathrm{var}_{true}\left\{\frac{\frac{\partial^2 \ell_i(\rho_0, \boldsymbol{\tau}_0)}{\partial \rho^2} - E_{true}\left(\frac{\partial^2 \ell_i(\boldsymbol{\tau}_0, \rho_0)}{\partial \rho^2}\right)}{n} I\left(\left|\frac{\partial^2 \ell_i\left(\rho_0, \boldsymbol{\tau}_0\right)}{\partial \rho^2} - E_{true}\left(\frac{\partial^2 \ell_i\left(\boldsymbol{\tau}_0, \rho_0\right)}{\partial \rho^2}\right)\right| \leq n\kappa\right)\right\} \to 0,$$

for every $\epsilon > 0$ and some $\kappa > 0$; and the expectations and variances are finite. From equation (9), we have

$$\begin{aligned}
E_{true}\left(\frac{\partial^2 \ell_i\left(\boldsymbol{\tau}_0, \rho_0\right)}{\partial \rho^2}\right) &= \sum_{a,b} \pi_{ab}\left(\boldsymbol{\tau}_0, \rho_{true}\right) \frac{\partial^2 \log \pi_{ab}\left(\boldsymbol{\tau}_0, \rho_0\right)}{\partial \rho^2} \\
&= E_0\left(\frac{\partial^2 \ell_i\left(\boldsymbol{\tau}_0, \rho_0\right)}{\partial \rho^2}\right) \\
&\quad + \sum_{a,b} \pi_{ab}\left(\boldsymbol{\tau}_0, \rho_0\right) \frac{\partial \log \pi_{ab}\left(\boldsymbol{\tau}_0, \rho_0\right)}{\partial \rho} \frac{\partial^2 \log \pi_{ab}\left(\boldsymbol{\tau}_0, \rho_0\right)}{\partial \rho^2} \delta^*/\sqrt{n} \\
&\quad + \sum_{a,b} \pi_{ab}\left(\boldsymbol{\tau}_0, \rho_0\right) \frac{\partial^2 \log \pi_{ab}\left(\boldsymbol{\tau}_0, \rho_0\right)}{\partial \rho^2} R_{ab}\left(\delta^*\right), \qquad (28) \\
\mathrm{var}_{true}\left(\frac{\partial^2 \ell_i\left(\boldsymbol{\tau}_0, \rho_0\right)}{\partial \rho^2}\right) &= E_{true}\left[\left(\frac{\partial^2 \ell_i\left(\boldsymbol{\tau}_0, \rho_0\right)}{\partial \rho^2}\right)^2\right] - \left[E_{true}\left(\frac{\partial^2 \ell_i\left(\boldsymbol{\tau}_0, \rho_0\right)}{\partial \rho^2}\right)\right]^2 \\
&= E_0\left[\left(\frac{\partial^2 \ell_i\left(\boldsymbol{\tau}_0, \rho_0\right)}{\partial \rho^2}\right)^2\right] - \left[E_{true}\left(\frac{\partial^2 \ell_i\left(\boldsymbol{\tau}_0, \rho_0\right)}{\partial \rho^2}\right)\right]^2 \\
&\quad + \sum_{a,b} \pi_{ab}\left(\boldsymbol{\tau}_0, \rho_0\right) \frac{\partial \log \pi_{ab}\left(\boldsymbol{\tau}_0, \rho_0\right)}{\partial \rho} \left(\frac{\partial^2 \log \pi_{ab}\left(\boldsymbol{\tau}_0, \rho_0\right)}{\partial \rho^2}\right)^2 \delta^*/\sqrt{n} \\
&\quad + \sum_{a,b} \pi_{ab}\left(\boldsymbol{\tau}_0, \rho_0\right) \left(\frac{\partial^2 \log \pi_{ab}\left(\boldsymbol{\tau}_0, \rho_0\right)}{\partial \rho^2}\right)^2 R_{ab}\left(\delta^*\right). \qquad (29)
\end{aligned}$$

By Result 1, the first- and second-order partial derivatives of $\log \pi_{ab}\left(\boldsymbol{\tau}, \rho\right)$ are bounded on

$O^{(\tau)} \times O^{(\rho)}$. Hence,

$$E_0 \left(\frac{\partial^2 \ell_i (\boldsymbol{\tau}_0, \rho_0)}{\partial \rho^2} \right) = \sum_{a,b} \pi_{ab} (\boldsymbol{\tau}_0, \rho_0) \frac{\partial^2 \log \pi_{ab} (\boldsymbol{\tau}_0, \rho_0)}{\partial \rho^2},$$

$$E_0 \left[\left(\frac{\partial^2 \ell_i (\boldsymbol{\tau}_0, \rho_0)}{\partial \rho^2} \right)^2 \right] = \sum_{a,b} \pi_{ab} (\boldsymbol{\tau}_0, \rho_0) \left[\frac{\partial^2 \log \pi_{ab} (\boldsymbol{\tau}_0, \rho_0)}{\partial \rho^2} \right]^2,$$

$$\sum_{a,b} \pi_{ab} (\boldsymbol{\tau}_0, \rho_0) \frac{\partial \log \pi_{ab} (\boldsymbol{\tau}_0, \rho_0)}{\partial \rho} \frac{\partial^2 \log \pi_{ab} (\boldsymbol{\tau}_0, \rho_0)}{\partial \rho^2},$$

$$\sum_{a,b} \pi_{ab} (\boldsymbol{\tau}_0, \rho_0) \frac{\partial \log \pi_{ab} (\boldsymbol{\tau}_0, \rho_0)}{\partial \rho} \left(\frac{\partial^2 \log \pi_{ab} (\boldsymbol{\tau}_0, \rho_0)}{\partial \rho^2} \right)^2,$$

are bounded, so as the variance $\mathrm{var}_0 \left(\frac{\partial^2 \ell_i (\boldsymbol{\tau}_0, \rho_0)}{\partial \rho^2} \right)$. Further, since $R_{ab} (\delta^*) = O (n^{-1})$ in (9),

$$\begin{aligned} \sum_{a,b} \pi_{ab} (\boldsymbol{\tau}_0, \rho_0) \frac{\partial^2 \log \pi_{ab} (\boldsymbol{\tau}_0, \rho_0)}{\partial \rho^2} R_{ab} (\delta^*) &= O \left(n^{-1} \right), \\ \sum_{a,b} \pi_{ab} (\boldsymbol{\tau}_0, \rho_0) \left(\frac{\partial^2 \log \pi_{ab} (\boldsymbol{\tau}_0, \rho_0)}{\partial \rho^2} \right)^2 R_{ab} (\delta^*) &= O \left(n^{-1} \right). \end{aligned}$$

Therefore,

$$\begin{aligned} E_{true} \left(\frac{\partial^2 \ell_i (\boldsymbol{\tau}_0, \rho_0)}{\partial \rho^2} \right) &= E_0 \left(\frac{\partial^2 \ell_i (\boldsymbol{\tau}_0, \rho_0)}{\partial \rho^2} \right) + O \left(n^{-1/2} \right), \\ \mathrm{var}_{true} \left(\frac{\partial^2 \ell_i (\boldsymbol{\tau}_0, \rho_0)}{\partial \rho^2} \right) &= \mathrm{var}_0 \left(\frac{\partial^2 \ell_i (\boldsymbol{\tau}_0, \rho_0)}{\partial \rho^2} \right) + O \left(n^{-1/2} \right), \end{aligned}$$

and they are both finite. Since both $\partial^2 \ell_i (\rho_0, \boldsymbol{\tau}_0) / \partial \rho^2$ and $E_{true} [\partial^2 \ell_i (\boldsymbol{\tau}_0, \rho_0) / \partial \rho^2]$ are finite and bounded,

$$\left| \frac{\partial^2 \ell_i (\rho_0, \boldsymbol{\tau}_0)}{\partial \rho^2} - E_{true} \left(\frac{\partial^2 \ell_i (\boldsymbol{\tau}_0, \rho_0)}{\partial \rho^2} \right) \right| < n\epsilon$$

for a sufficiently large n. Consequently, (27) holds since

$$\begin{aligned} \sum_{i=1}^{n} E_{true} \left\{ \frac{\frac{\partial^2 \ell_i (\rho_0, \boldsymbol{\tau}_0)}{\partial \rho^2} - E_{true} \left(\frac{\partial^2 \ell_i (\boldsymbol{\tau}_0, \rho_0)}{\partial \rho^2} \right)}{n} \right\} &= 0, \\ \sum_{i=1}^{n} \mathrm{var}_{true} \left\{ \frac{\frac{\partial^2 \ell_i (\rho_0, \boldsymbol{\tau}_0)}{\partial \rho^2} - E_{true} \left(\frac{\partial^2 \ell_i (\boldsymbol{\tau}_0, \rho_0)}{\partial \rho^2} \right)}{n} \right\} &= \frac{1}{n} \mathrm{var}_{true} \left(\frac{\partial^2 \ell_i (\rho_0, \boldsymbol{\tau}_0)}{\partial \rho^2} \right) \to 0. \end{aligned}$$

Likewise, similar arguments yields

$$\frac{1}{n} \frac{\partial^2 \ell (\rho_0, \boldsymbol{\tau}_0)}{\partial \rho \partial \boldsymbol{\tau}} - E_{true} \left(\frac{\partial^2 \ell_i (\boldsymbol{\tau}_0, \rho_0)}{\partial \rho \partial \boldsymbol{\tau}} \right) \xrightarrow{p} \mathbf{0}. \tag{30}$$

Consequently, $\boldsymbol{\Lambda}$ converges in probability to

$$\begin{aligned}\boldsymbol{\Gamma} &= \boldsymbol{A}^T + \mathbf{1}_g E_0\left(\frac{\partial^2 \ell_i\left(\boldsymbol{\tau}_0,\rho_0\right)}{\partial\rho\partial\boldsymbol{\tau}_g^T}\right)\left(\boldsymbol{B}_{g,0}^T\boldsymbol{D}_{g,0}^{-1}\boldsymbol{B}_{g,0}\right)^{-1}\boldsymbol{B}_{g,0}^T\boldsymbol{D}_{g,0}^{-1} \\ &\quad + \boldsymbol{D}_{h,0}^{-1}\boldsymbol{B}_{h,0}\left(\boldsymbol{B}_{h,0}^T\boldsymbol{D}_{h,0}^{-1}\boldsymbol{B}_{h,0}\right)^{-1} E_0\left(\frac{\partial^2 \ell_i\left(\boldsymbol{\tau}_0,\rho_0\right)}{\partial\rho\partial\boldsymbol{\tau}_h}\right)\mathbf{1}_h^T, \end{aligned} \tag{31}$$

which coincides with equation (16) in Jöreskog (1994), as if the local parameter is zero. Further, by the Slutsky's theorem (Theorem 2.13, Jiang, 2010),

$$\begin{aligned}\sqrt{n}\mathrm{tr}\left\{\boldsymbol{\Lambda}\left(\boldsymbol{P}-\boldsymbol{\pi}_0\right)\right\} &= \sqrt{n}\mathrm{tr}\left\{\boldsymbol{\Gamma}\left(\boldsymbol{P}-\boldsymbol{\pi}_0\right)\right\} + \sqrt{n}\mathrm{tr}\left\{\left(\boldsymbol{\Lambda}-\boldsymbol{\Gamma}\right)\left(\boldsymbol{P}-\boldsymbol{\pi}_0\right)\right\} \\ &= \sqrt{n}\mathrm{tr}\left\{\boldsymbol{\Gamma}\left(\boldsymbol{P}-\boldsymbol{\pi}_0\right)\right\} + o_{\mathrm{P}}(1),\end{aligned}$$

if $\sqrt{n}\left(\boldsymbol{P}-\boldsymbol{\pi}_0\right)$ converges in distribution to some random variable. Consequently, equation (26) becomes

$$\frac{1}{\sqrt{n}}\frac{\partial \ell\left(\rho_0,\boldsymbol{\tau}_0\right)}{\partial\rho} + \left[\frac{1}{n}\frac{\partial^2 \ell\left(\rho_0,\boldsymbol{\tau}_0\right)}{\partial\rho\partial\boldsymbol{\tau}^T}\right]\sqrt{n}\left(\hat{\boldsymbol{\tau}}-\boldsymbol{\tau}_0\right) = \sqrt{n}\mathrm{tr}\left\{\boldsymbol{\Gamma}\left(\boldsymbol{P}-\boldsymbol{\pi}_0\right)\right\} + o_{\mathrm{P}}(1). \tag{32}$$

We now consider the last term in the numerator and the last term in the denominator of (24). Let the operator vec () vectorize the enclosed matrix by stacking columns on top of each other. In order to derive the asymptotic distribution of $\sqrt{n}\mathrm{vec}\left(\boldsymbol{P}-\boldsymbol{\pi}_0\right)$, we assume that a random vector $\boldsymbol{z}_n$ follows a multinomial distribution with one trial and probabilities $\mathrm{vec}\left(\boldsymbol{\pi}_{true}\right)$ with $\boldsymbol{\pi}_{true} = \boldsymbol{\pi}\left(\boldsymbol{\tau}_0,\rho_{true}\right)$, and that the observed relative frequency is $\mathrm{vec}\left(\boldsymbol{P}\right)$. Here the subscript n is used to emphasize that the distribution of $\boldsymbol{z}_n$ depends on n. We also define $\boldsymbol{z}_0$ as a multinomial distribution with probabilities $\mathrm{vec}\left(\boldsymbol{\pi}_0\right)$ with $\boldsymbol{\pi}_0 = \boldsymbol{\pi}\left(\boldsymbol{\tau}_0,\rho_0\right)$. Because of (9), we have

$$E_{true}\left(\boldsymbol{z}_n\right) = \mathrm{vec}\left(\boldsymbol{\pi}_{true}\right) = E_0\left(\boldsymbol{z}_0\right) + \mathrm{diag}\left(\mathrm{vec}\left(\boldsymbol{\pi}_0\right)\right)\frac{\partial\log\left\{\mathrm{vec}\left(\boldsymbol{\pi}_0\right)\right\}}{\partial\rho}\delta^*/\sqrt{n} + O\left(n^{-1}\right). \tag{33}$$

Since $\partial\log\left\{\mathrm{vec}\left(\boldsymbol{\pi}_0\right)\right\}/\partial\rho$ is bounded by Result 1, $E_{true}\left(\boldsymbol{z}_n\right) = E_0\left(\boldsymbol{z}_0\right) + O\left(n^{-1/2}\right)$. Note that

$$\begin{aligned}\mathrm{var}_{true}\left(\boldsymbol{z}_n\right) &= E_{true}\left(\boldsymbol{z}_n\boldsymbol{z}_n^T\right) - E_{true}\left(\boldsymbol{z}_n\right)E_{true}\left(\boldsymbol{z}_n^T\right) \\ &= \mathrm{diag}\left\{\mathrm{vec}\left(\boldsymbol{\pi}_{true}\right)\right\} - \mathrm{vec}\left(\boldsymbol{\pi}_{true}\right)\mathrm{vec}^T\left(\boldsymbol{\pi}_{true}\right).\end{aligned}$$

By (33), we get

$$\begin{aligned}
\mathrm{var}_{true}\left(\boldsymbol{z}_n\right) &= \mathrm{diag}\left\{\mathrm{vec}\left(\boldsymbol{\pi}_{true}\right)\right\} - \mathrm{vec}\left(\boldsymbol{\pi}_{true}\right)\mathrm{vec}^T\left(\boldsymbol{\pi}_{true}\right) \\
&= \mathrm{diag}\left\{E_0\left(\boldsymbol{z}_0\right) + O\left(n^{-1/2}\right)\right\} - \left[E_0\left(\boldsymbol{z}_0\right) + O\left(n^{-1/2}\right)\right]\left[E_0\left(\boldsymbol{z}_0\right) + O\left(n^{-1/2}\right)\right]^T \\
&= \mathrm{var}_0\left(\boldsymbol{z}_0\right) + O\left(n^{-1/2}\right). \qquad (34)
\end{aligned}$$

If we observe $x_g = a$ and $x_h = b$,

$$\left\|\frac{\boldsymbol{z}_n - E_{true}\left(\boldsymbol{z}_n\right)}{\sqrt{n}}\right\|^2 = \frac{\left\|\mathrm{vec}\left(\boldsymbol{\pi}_{true}\right)\right\|^2 - \pi_{ab}^2\left(\boldsymbol{\tau}_0, \rho_{true}\right) + \left(1 - \pi_{ab}\left(\boldsymbol{\tau}_0, \rho_{true}\right)\right)^2}{n} \le \frac{2}{n}.$$

Hence, the Lindeberg condition holds, since, for every $\epsilon > 0$,

$$\left\|\frac{\boldsymbol{z}_{n,i} - E_{true}\left(\boldsymbol{z}_{n,i}\right)}{\sqrt{n}}\right\|^2 < \epsilon^2$$

and

$$\sum_{i=1}^n E\left[\left\|\frac{\boldsymbol{z}_{n,i} - E_{true}\left(\boldsymbol{z}_{n,i}\right)}{\sqrt{n}}\right\|^2 I\left\{\left\|\frac{\boldsymbol{z}_{n,i} - E_{true}\left(\boldsymbol{z}_{n,i}\right)}{\sqrt{n}}\right\|^2 \ge \epsilon^2\right\}\right] = 0,$$

for a sufficiently large n. The diagonal and off-diagonal entries in the covariance matrix of $\boldsymbol{z}_{n,i}$ are $\pi_a\left(\boldsymbol{\tau}_0, \rho_{true}\right)\left[1 - \pi_a\left(\boldsymbol{\tau}_0, \rho_{true}\right)\right]$ and $-\pi_a\left(\boldsymbol{\tau}_0, \rho_{true}\right)\pi_b\left(\boldsymbol{\tau}_0, \rho_{true}\right)$, respectively. Note that

$$\sum_{i=1}^n \mathrm{var}_{true}\left(\frac{\boldsymbol{z}_{n,i} - E_{true}\left(\boldsymbol{z}_{n,i}\right)}{\sqrt{n}}\right) = \mathrm{var}_{true}\left(\boldsymbol{z}_{n,i}\right) \to \mathrm{var}_0\left(\boldsymbol{z}_0\right)$$

by (34). Therefore, the central limit theorem implies

$$\frac{1}{\sqrt{n}}\sum_{i=1}^n \left[\boldsymbol{z}_{n,i} - E_{true}\left(\boldsymbol{z}_{n,i}\right)\right] \xrightarrow{d} N\left(\mathbf{0}, \mathrm{var}_0\left(\boldsymbol{z}_0\right)\right).$$

Since $n^{-1}\sum_{i=1}^n \boldsymbol{z}_{n,i} = \mathrm{vec}\left(\boldsymbol{P}\right)$, we have

$$\sqrt{n}\left(\mathrm{vec}\left(\boldsymbol{P}\right) - \mathrm{vec}\left(\boldsymbol{\pi}_0\right) - \mathrm{diag}\left(\mathrm{vec}\left(\boldsymbol{\pi}_0\right)\right)\frac{\partial \log\left\{\mathrm{vec}\left(\boldsymbol{\pi}_0\right)\right\}}{\partial\rho}\delta^*/\sqrt{n}\right) \xrightarrow{d} N\left(\mathbf{0}, \mathrm{var}_0\left(\boldsymbol{z}_0\right)\right). \quad (35)$$

Equivalently,

$$\sqrt{n}\left(\mathrm{vec}\left(\boldsymbol{P}\right) - \mathrm{vec}\left(\boldsymbol{\pi}_0\right)\right) \xrightarrow{d} N\left(\mathrm{diag}\left(\mathrm{vec}\left(\boldsymbol{\pi}_0\right)\right)\frac{\partial \log\left\{\mathrm{vec}\left(\boldsymbol{\pi}_0\right)\right\}}{\partial\rho}\delta^*, \mathrm{var}_0\left(\boldsymbol{z}_0\right)\right). \quad (36)$$

Hence, the Slutsky's theorem (Theorem 2.13, Jiang, 2010) leads to

$$\begin{aligned}\sqrt{n}\mathrm{tr}\left\{\boldsymbol{\Gamma}\left(\boldsymbol{P}-\boldsymbol{\pi}_0\right)\right\} &= \mathrm{vec}^T\left(\boldsymbol{\Gamma}\right)\sqrt{n}\mathrm{vec}\left(\boldsymbol{P}-\boldsymbol{\pi}_0\right) \\ &\xrightarrow{d} N\left(\mathrm{vec}^T\left(\boldsymbol{\Gamma}\right)\mathrm{diag}\left(\mathrm{vec}\left(\boldsymbol{\pi}_0\right)\right)\frac{\partial \log\left\{\mathrm{vec}\left(\boldsymbol{\pi}_0\right)\right\}}{\partial \rho}\delta^*, \mathrm{vec}^T\left(\boldsymbol{\Gamma}\right)\mathrm{var}_0\left(\boldsymbol{z}_0\right)\mathrm{vec}\left(\boldsymbol{\Gamma}\right)\right).\end{aligned} \tag{37}$$

Note that

$$\begin{aligned}\frac{\partial^3 \ell_i\left(\tilde{\boldsymbol{\tau}},\tilde{\rho}\right)}{\partial\rho\partial\boldsymbol{\tau}\partial\boldsymbol{\tau}^T} = & \sum_{a=1}^{m_x}\sum_{b=1}^{m_y} I_i\left(a,b\right)\left[\frac{1}{\pi_{ab}\left(\tilde{\boldsymbol{\tau}},\tilde{\rho}\right)}\frac{\partial^3\pi_{ab}\left(\tilde{\boldsymbol{\tau}},\tilde{\rho}\right)}{\partial\rho\partial\boldsymbol{\tau}\partial\boldsymbol{\tau}^T}\right. \\ & -\frac{1}{\pi_{ab}^2\left(\tilde{\boldsymbol{\tau}},\tilde{\rho}\right)}\frac{\partial^2\pi_{ab}\left(\tilde{\boldsymbol{\tau}},\tilde{\rho}\right)}{\partial\rho\partial\boldsymbol{\tau}}\frac{\partial\log\pi_{ab}\left(\tilde{\boldsymbol{\tau}},\tilde{\rho}\right)}{\partial\boldsymbol{\tau}^T} \\ & \left.-\frac{\partial^2\log\pi_{ab}\left(\tilde{\boldsymbol{\tau}},\tilde{\rho}\right)}{\partial\boldsymbol{\tau}\partial\boldsymbol{\tau}^T}\frac{\partial\log\pi_{ab}\left(\tilde{\boldsymbol{\tau}},\tilde{\rho}\right)}{\partial\rho}-\frac{\partial\log\pi_{ab}\left(\tilde{\boldsymbol{\tau}},\tilde{\rho}\right)}{\partial\boldsymbol{\tau}}\frac{\partial^2\log\pi_{ab}\left(\tilde{\boldsymbol{\tau}},\tilde{\rho}\right)}{\partial\rho\partial\boldsymbol{\tau}^T}\right],\end{aligned}$$

where all partial derivatives are finite and bounded on $O^{(\tau)}\times O^{(\rho)}$ by Result 1. Then, $n^{-1}\partial^3\ell\left(\tilde{\boldsymbol{\tau}},\tilde{\rho}\right)/\partial\rho\partial\boldsymbol{\tau}\partial\boldsymbol{\tau}^T$ is bounded in probability. Likewise, $n^{-1}\partial^3\ell\left(\tilde{\boldsymbol{\tau}},\tilde{\rho}\right)/\partial\rho^3$ and $n^{-1}\partial^3\ell\left(\tilde{\boldsymbol{\tau}},\tilde{\rho}\right)/\partial\rho^2\partial\boldsymbol{\tau}$ are also bounded in probability. Consequently,

$$\sqrt{n}\left(\hat{\boldsymbol{\tau}}-\boldsymbol{\tau}_0\right)^T\left[\frac{1}{n}\frac{\partial^3\ell\left(\tilde{\boldsymbol{\tau}},\tilde{\rho}\right)}{\partial\rho\partial\boldsymbol{\tau}\partial\boldsymbol{\tau}^T}\right]\left(\hat{\boldsymbol{\tau}}-\boldsymbol{\tau}_0\right) \xrightarrow{p} 0, \tag{38}$$

$$\frac{1}{n}\frac{\partial^2\ell\left(\boldsymbol{\tau}_0,\rho_0\right)}{\partial\rho^2}+\frac{1}{2n}\frac{\partial^3\ell\left(\tilde{\boldsymbol{\tau}},\tilde{\rho}\right)}{\partial\rho^3}\left(\hat{\rho}-\rho_0\right)+\frac{1}{n}\frac{\partial^3\ell\left(\tilde{\boldsymbol{\tau}},\tilde{\rho}\right)}{\partial\rho^2\partial\boldsymbol{\tau}^T}\left(\hat{\boldsymbol{\tau}}-\boldsymbol{\tau}_0\right) \xrightarrow{p} E_0\left(\frac{\partial^2\ell_i\left(\boldsymbol{\tau}_0,\rho_0\right)}{\partial\rho^2}\right) \tag{39}$$

since $\hat{\rho}-\rho_0=o_{\mathrm{P}}\left(1\right)$ by Theorem 1, $\sqrt{n}\left(\hat{\boldsymbol{\tau}}-\boldsymbol{\tau}_0\right)=O_{\mathrm{P}}\left(1\right)$ from (22) and (23), and (27) holds. By equation (39) and $E_0\left(\frac{\partial^2\ell_i(\boldsymbol{\tau}_0,\rho_0)}{\partial\rho^2}\right)\neq 0$ under Assumption A4, the denominator of (24) is nonzero with probability approaching 1. By equations (32) and (38), the numerator of (24) becomes

$$\sqrt{n}\mathrm{tr}\left\{\boldsymbol{\Gamma}\left(\boldsymbol{P}-\boldsymbol{\pi}_0\right)\right\}+o_{\mathrm{P}}\left(1\right),$$

which converges in distribution to the normal distribution (37) by the Slutsky's theorem. The denominator of (24) converges in probability to a constant by (39). Hence, by the corollary of the Slutsky's theorem in Ferguson (1996, P40), we obtain

$$\sqrt{n}\left(\hat{\rho}-\rho_0\right) = \frac{1}{-E_0\left(\frac{\partial^2\ell_i(\boldsymbol{\tau}_0,\rho_0)}{\partial\rho^2}\right)}\sqrt{n}\mathrm{tr}\left\{\boldsymbol{\Gamma}\left(\boldsymbol{P}-\boldsymbol{\pi}_0\right)\right\}+o_{\mathrm{P}}\left(1\right).$$

Therefore,

$$\sqrt{n}\left(\hat{\rho}-\rho_0\right) \quad \xrightarrow{d} \quad N\left(\frac{\mathrm{vec}^T\left(\boldsymbol{\Gamma}\right)\mathrm{diag}\left(\mathrm{vec}\left(\boldsymbol{\pi}_0\right)\right)}{-E_0\left(\frac{\partial^2\ell_i(\boldsymbol{\tau}_0,\rho_0)}{\partial\rho^2}\right)}\frac{\partial\log\left\{\mathrm{vec}\left(\boldsymbol{\pi}_0\right)\right\}}{\partial\rho}\delta^*, \frac{\mathrm{vec}^T\left(\boldsymbol{\Gamma}\right)\mathrm{var}_0\left(\boldsymbol{z}_0\right)\mathrm{vec}\left(\boldsymbol{\Gamma}\right)}{\left[E_0\left(\frac{\partial^2\ell_i(\boldsymbol{\tau}_0,\rho_0)}{\partial\rho^2}\right)\right]^2}\right),$$

the asymptotic covariance matrix of which is in line with the result in Jöreskog (1994) for the standard asymptotic framework. Note that

$$\begin{aligned}
&\mathrm{vec}^T\left[\boldsymbol{1}_g E_0\left(\frac{\partial^2\ell_i\left(\boldsymbol{\tau}_0,\rho_0\right)}{\partial\rho\partial\boldsymbol{\tau}_g^T}\right)\left(\boldsymbol{B}_{g,0}^T\boldsymbol{D}_{g,0}^{-1}\boldsymbol{B}_{g,0}\right)^{-1}\boldsymbol{B}_{g,0}^T\boldsymbol{D}_{g,0}^{-1}\right]\mathrm{diag}\left(\mathrm{vec}\left(\boldsymbol{\pi}_0\right)\right)\frac{\partial\log\left\{\mathrm{vec}\left(\boldsymbol{\pi}_0\right)\right\}}{\partial\rho}\\
=\ &\mathrm{vec}^T\left[\boldsymbol{1}_g E_0\left(\frac{\partial^2\ell_i\left(\boldsymbol{\tau}_0,\rho_0\right)}{\partial\rho\partial\boldsymbol{\tau}_g^T}\right)\left(\boldsymbol{B}_{g,0}^T\boldsymbol{D}_{g,0}^{-1}\boldsymbol{B}_{g,0}\right)^{-1}\boldsymbol{B}_{g,0}^T\boldsymbol{D}_{g,0}^{-1}\right]\frac{\partial\mathrm{vec}\left(\boldsymbol{\pi}_0\right)}{\partial\rho}\\
=\ &\mathrm{tr}\left\{\boldsymbol{D}_{g,0}^{-1}\boldsymbol{B}_{g,0}\left(\boldsymbol{B}_{g,0}^T\boldsymbol{D}_{g,0}^{-1}\boldsymbol{B}_{g,0}\right)^{-1}E_0\left(\frac{\partial^2\ell_i\left(\boldsymbol{\tau}_0,\rho_0\right)}{\partial\rho\partial\boldsymbol{\tau}_g}\right)\boldsymbol{1}_g^T\frac{\partial\boldsymbol{\pi}_0}{\partial\rho}\right\}, \qquad (40)
\end{aligned}$$

and

$$\begin{aligned}
&\mathrm{vec}^T\left[\boldsymbol{D}_{h,0}^{-1}\boldsymbol{B}_{h,0}\left(\boldsymbol{B}_{h,0}^T\boldsymbol{D}_{h,0}^{-1}\boldsymbol{B}_{h,0}\right)^{-1}E_0\left(\frac{\partial^2\ell_i\left(\boldsymbol{\tau}_0,\rho_0\right)}{\partial\rho\partial\boldsymbol{\tau}_h}\right)\boldsymbol{1}_h^T\right]\mathrm{diag}\left(\mathrm{vec}\left(\boldsymbol{\pi}_0\right)\right)\frac{\partial\log\left\{\mathrm{vec}\left(\boldsymbol{\pi}_0\right)\right\}}{\partial\rho}\\
=\ &\mathrm{vec}^T\left[\boldsymbol{D}_{h,0}^{-1}\boldsymbol{B}_{h,0}\left(\boldsymbol{B}_{h,0}^T\boldsymbol{D}_{h,0}^{-1}\boldsymbol{B}_{h,0}\right)^{-1}E_0\left(\frac{\partial^2\ell_i\left(\boldsymbol{\tau}_0,\rho_0\right)}{\partial\rho\partial\boldsymbol{\tau}_h}\right)\boldsymbol{1}_h^T\right]\frac{\partial\mathrm{vec}\left(\boldsymbol{\pi}_0\right)}{\partial\rho}\\
=\ &\mathrm{tr}\left\{\boldsymbol{1}_h E_0\left(\frac{\partial^2\ell_i\left(\boldsymbol{\tau}_0,\rho_0\right)}{\partial\rho\partial\boldsymbol{\tau}_h^T}\right)\left(\boldsymbol{B}_{h,0}^T\boldsymbol{D}_{h,0}^{-1}\boldsymbol{B}_{h,0}\right)^{-1}\boldsymbol{B}_{h,0}^T\boldsymbol{D}_{h,0}^{-1}\frac{\partial\boldsymbol{\pi}_0}{\partial\rho}\right\}\\
=\ &\mathrm{tr}\left\{E_0\left(\frac{\partial^2\ell_i\left(\boldsymbol{\tau}_0,\rho_0\right)}{\partial\rho\partial\boldsymbol{\tau}_h^T}\right)\left(\boldsymbol{B}_{h,0}^T\boldsymbol{D}_{h,0}^{-1}\boldsymbol{B}_{h,0}\right)^{-1}\boldsymbol{B}_{h,0}^T\boldsymbol{D}_{h,0}^{-1}\frac{\partial\boldsymbol{\pi}_0}{\partial\rho}\boldsymbol{1}_h\right\}. \qquad (41)
\end{aligned}$$

Since

$$\boldsymbol{1}_g^T\frac{\partial\boldsymbol{\pi}\left(\boldsymbol{\tau}_0,\rho_0\right)}{\partial\rho}=\boldsymbol{0} \quad \text{and} \quad \frac{\partial\boldsymbol{\pi}\left(\boldsymbol{\tau}_0,\rho_0\right)}{\partial\rho}\boldsymbol{1}_h=\boldsymbol{0},$$

we can see that (40) and (41) are both 0. Further,

$$\begin{aligned}
\mathrm{vec}^T\left(\boldsymbol{A}^T\right)\mathrm{diag}\left(\mathrm{vec}\left(\boldsymbol{\pi}_0\right)\right)\frac{\partial\log\left\{\mathrm{vec}\left(\boldsymbol{\pi}_0\right)\right\}}{\partial\rho} &= \mathrm{vec}^T\left(\boldsymbol{A}^T\right)\frac{\partial\mathrm{vec}\left(\boldsymbol{\pi}_0\right)}{\partial\rho}\\
&= \mathrm{tr}\left\{\frac{\partial\log\left(\boldsymbol{\pi}_0^T\right)}{\partial\rho}\frac{\partial\boldsymbol{\pi}_0}{\partial\rho}\right\}\\
&= -E_0\left(\frac{\partial^2\ell_i\left(\boldsymbol{\tau}_0,\rho_0\right)}{\partial\rho^2}\right),
\end{aligned}$$

since

$$\frac{\partial^2\ell_i\left(\boldsymbol{\tau}_0,\rho_0\right)}{\partial\rho^2} = \sum_{a,b}\left[\frac{I_i\left(a,b\right)}{\pi_{ab}\left(\boldsymbol{\tau}_0,\rho_0\right)}\frac{\partial^2\pi_{ab}\left(\boldsymbol{\tau}_0,\rho_0\right)}{\partial\rho^2}-\frac{I_i\left(a,b\right)}{\pi_{ab}^2\left(\boldsymbol{\tau}_0,\rho_0\right)}\left(\frac{\partial\pi_{ab}\left(\boldsymbol{\tau}_0,\rho_0\right)}{\partial\rho}\right)^2\right]$$

and

$$E_0\left(\frac{\partial^2 \ell_i(\boldsymbol{\tau}_0,\rho_0)}{\partial\rho^2}\right) = -\sum_{a,b}\left[\frac{1}{\pi_{ab}(\boldsymbol{\tau}_0,\rho_0)}\left(\frac{\partial\pi_{ab}(\boldsymbol{\tau}_0,\rho_0)}{\partial\rho}\right)^2\right] = -\sum_{a,b}\left[\frac{\partial\log\pi_{ab}(\boldsymbol{\tau}_0,\rho_0)}{\partial\rho}\frac{\partial\pi_{ab}(\boldsymbol{\tau}_0,\rho_0)}{\partial\rho}\right].$$

Therefore, using the expression of $\boldsymbol{\Gamma}$ given by (31), we get

$$\begin{aligned}
&\mathrm{vec}^T(\boldsymbol{\Gamma})\,\mathrm{diag}(\mathrm{vec}(\boldsymbol{\pi}_0))\frac{\partial\log\{\mathrm{vec}(\boldsymbol{\pi}_0)\}}{\partial\rho}\\
=\;&\mathrm{vec}^T\left(\boldsymbol{A}^T\right)\mathrm{diag}(\mathrm{vec}(\boldsymbol{\pi}_0))\frac{\partial\log\{\mathrm{vec}(\boldsymbol{\pi}_0)\}}{\partial\rho}\\
&+\mathrm{vec}^T\left(\mathbf{1}_g E_0\left(\frac{\partial^2\ell_i(\boldsymbol{\tau}_0,\rho_0)}{\partial\rho\partial\boldsymbol{\tau}_g^T}\right)\left(\boldsymbol{B}_{g,0}^T\boldsymbol{D}_{g,0}^{-1}\boldsymbol{B}_{g,0}\right)^{-1}\boldsymbol{B}_{g,0}^T\boldsymbol{D}_{g,0}^{-1}\right)\mathrm{diag}(\mathrm{vec}(\boldsymbol{\pi}_0))\frac{\partial\log\{\mathrm{vec}(\boldsymbol{\pi}_0)\}}{\partial\rho}\\
&+\mathrm{vec}^T\left(\boldsymbol{D}_{h,0}^{-1}\boldsymbol{B}_{h,0}\left(\boldsymbol{B}_{h,0}^T\boldsymbol{D}_{h,0}^{-1}\boldsymbol{B}_{h,0}\right)^{-1}E_0\left(\frac{\partial^2\ell_i(\boldsymbol{\tau}_0,\rho_0)}{\partial\rho\partial\boldsymbol{\tau}_h}\right)\mathbf{1}_h^T\right)\mathrm{diag}(\mathrm{vec}(\boldsymbol{\pi}_0))\frac{\partial\log\{\mathrm{vec}(\boldsymbol{\pi}_0)\}}{\partial\rho}\\
=\;&-E_0\left(\frac{\partial^2\ell_i(\boldsymbol{\tau}_0,\rho_0)}{\partial\rho^2}\right),
\end{aligned}$$

which means that the mean of the asymptotic distribution of $\sqrt{n}(\hat{\rho}-\rho_0)$ is equivalent to δ^*. In other words,

$$\sqrt{n}(\hat{\rho}-\rho_{true}) \xrightarrow{d} N\left(0,\frac{\mathrm{vec}^T(\boldsymbol{\Gamma})\,\mathrm{var}_0(\boldsymbol{z}_0)\,\mathrm{vec}(\boldsymbol{\Gamma})}{\left[E_0\left(\frac{\partial^2\ell_i(\boldsymbol{\tau}_0,\rho_0)}{\partial\rho^2}\right)\right]^2}\right).$$

The proof for the polychoric correlation coefficient estimator between two variables is completed.

Regarding the asymptotic distribution of more than one polychoric correlation estimators, the proof by and large follows from the one estimator case. Our proof above implies that

$$\sqrt{n}(\hat{\rho}_{gh}-\rho_{gh,0}) = \frac{1}{-E_0\left(\frac{\partial^2\ell_i\left(\tau_{gh,0},\rho_{gh,0}\right)}{\partial\rho^2}\right)}\sqrt{n}\mathrm{tr}\left\{\boldsymbol{\Gamma}^{(gh)}\left(\boldsymbol{P}^{(gh)}-\boldsymbol{\pi}_0^{(gh)}\right)\right\}+o_{\mathrm{P}}(1) \quad (42)$$

holds for any pair of gth indicator and hth indicator, provided that all regularity conditions

hold. Here indices are added to indicate the pair of indicators. Thus,

$$\begin{aligned}\begin{bmatrix}\sqrt{n}\left(\hat{\rho}_{12}-\rho_{12,0}\right)\\ \vdots \\ \sqrt{n}\left(\hat{\rho}_{q-1,q}-\rho_{q-1,q,0}\right)\end{bmatrix} &= \begin{bmatrix}\frac{1}{-E_0\left(\frac{\partial^2\ell_i\left(\tau_{12,0},\rho_{12,0}\right)}{\partial\rho^2}\right)}\sqrt{n}\mathrm{tr}\left\{\boldsymbol{\Gamma}^{(12)}\left(\boldsymbol{P}^{(12)}-\boldsymbol{\pi}_0^{(12)}\right)\right\}\\ \vdots \\ \frac{1}{-E_0\left(\frac{\partial^2\ell_i\left(\tau_{q-1,q,0},\rho_{q-1,q,0}\right)}{\partial\rho^2}\right)}\sqrt{n}\mathrm{tr}\left\{\boldsymbol{\Gamma}^{(q-1,q)}\left(\boldsymbol{P}^{(q-1,q)}-\boldsymbol{\pi}_0^{(q-1,q)}\right)\right\}\end{bmatrix}+o_{\mathrm{P}}(1)\\ &= -\sqrt{n}\boldsymbol{H}^{-1}\boldsymbol{E}\begin{bmatrix}\mathrm{vec}\left(\boldsymbol{P}^{(12)}-\boldsymbol{\pi}_0^{(12)}\right)\\ \mathrm{vec}\left(\boldsymbol{P}^{(13)}-\boldsymbol{\pi}_0^{(13)}\right)\\ \vdots \\ \mathrm{vec}\left(\boldsymbol{P}^{(q-1,q)}-\boldsymbol{\pi}_0^{(q-1,q)}\right)\end{bmatrix}+o_{\mathrm{P}}(1), \qquad (43)\end{aligned}$$

where

$$\begin{aligned}\boldsymbol{H} &= \mathrm{diag}\left(E_0\left(\frac{\partial^2\ell_i(\tau_{12,0},\rho_{12,0})}{\partial\rho^2}\right) \cdots E_0\left(\frac{\partial^2\ell_i(\tau_{q-1,q,0},\rho_{q-1,q,0})}{\partial\rho^2}\right)\right),\\ \boldsymbol{E} &= \begin{bmatrix}\mathrm{vec}^T\left(\boldsymbol{\Gamma}^{(12)}\right) & \mathbf{0} & \cdots & \mathbf{0}\\ \mathbf{0} & \mathrm{vec}^T\left(\boldsymbol{\Gamma}^{(13)}\right) & \cdots & 0\\ \vdots & \vdots & \ddots & \vdots\\ \mathbf{0} & \mathbf{0} & \cdots & \mathrm{vec}^T\left(\boldsymbol{\Gamma}^{(q-1,q)}\right)\end{bmatrix}.\end{aligned}$$

Hence, the asymptotic distribution of the vector of all polychoric correlation estimators $\sqrt{n}\left(\hat{\boldsymbol{\rho}}-\boldsymbol{\rho}_{true}\right)$ depends on the joint distribution of

$$\boldsymbol{p} = \left(\mathrm{vec}^T\left(\boldsymbol{P}^{(12)}-\boldsymbol{\pi}_0^{(12)}\right)\ \mathrm{vec}^T\left(\boldsymbol{P}^{(13)}-\boldsymbol{\pi}_0^{(13)}\right)\ \cdots\ \mathrm{vec}^T\left(\boldsymbol{P}^{(q-1,q)}-\boldsymbol{\pi}_0^{(q-1,q)}\right)\right)^T,$$

where $\boldsymbol{p}$ is a $\sum_{i=1}^{q}\sum_{j=i+1}^{q} m_i m_j \times 1$ vector. In the univariate case, we use $\boldsymbol{\pi}_{true}$ to denote $\boldsymbol{\pi}_{true}^{(gh)}$ and $\boldsymbol{\pi}_0$ to denote $\boldsymbol{\pi}_0^{(gh)}$. In the multivariate case, we redefine

$$\boldsymbol{\pi}_{true} = \left(\mathrm{vec}^T\left(\boldsymbol{\pi}_{true}^{(12)}\right)\ \mathrm{vec}^T\left(\boldsymbol{\pi}_{true}^{(13)}\right)\ \cdots\ \mathrm{vec}^T\left(\boldsymbol{\pi}_{true}^{(q-1,q)}\right)\right)^T, \qquad (44)$$

$$\boldsymbol{\pi}_0 = \left(\mathrm{vec}^T\left(\boldsymbol{\pi}_0^{(12)}\right)\ \mathrm{vec}^T\left(\boldsymbol{\pi}_0^{(13)}\right)\ \cdots\ \mathrm{vec}^T\left(\boldsymbol{\pi}_0^{(q-1,q)}\right)\right)^T, \qquad (45)$$

as a generalization of $\mathrm{vec}\left(\boldsymbol{\pi}_{true}^{(gh)}\right)$ and $\mathrm{vec}\left(\boldsymbol{\pi}_0^{(gh)}\right)$. Using the newly defined $\boldsymbol{\pi}_0$, we will first generalize (33) and (34) to multivariate cases. Let $\boldsymbol{y}_n^{(gh)}$ be the $m_g m_h \times 1$ vector with

entries $I\left\{x_g = a \text{ and } x_h = b\right\}$ for all possible combinations of a, and b. Let $\boldsymbol{y}_n$ be the $\sum_{i=1}^{q}\sum_{j=i+1}^{q} m_i m_j \times 1$ vector that stacks all $\boldsymbol{y}_n^{(gh)}$ on top of another for all possible pairs (g, h). Without loss of generality, we assume that the entries in $\boldsymbol{y}_n$ are sorted such that $E_{true}\left(\boldsymbol{y}_n\right) = \boldsymbol{\pi}_{true}$. We also define $\boldsymbol{y}_0^{(gh)}$ and $\boldsymbol{y}_0$ as analogues to $\boldsymbol{y}_n^{(gh)}$ and $\boldsymbol{y}_n$, respectively, but $E_0\left(\boldsymbol{y}_0^{(gh)}\right) = \text{vec}\left(\boldsymbol{\pi}_0^{(gh)}\right)$ and $E_0\left(\boldsymbol{y}_0\right) = \boldsymbol{\pi}_0$. $\boldsymbol{y}_n$ and $\boldsymbol{y}_0$ are simply the generalizations of $\boldsymbol{z}_n$ and $\boldsymbol{z}_0$ from the univariate case to the multivariate case. By stacking (33) for all pairs (g, h) from the univariate case, we get

$$E_{true}\left(\boldsymbol{y}_n\right) = E_0\left(\boldsymbol{y}_0\right) + \begin{pmatrix} \pi_{11,0}^{(12)} \frac{\partial \log \pi_{11}^{(12)}\left(\boldsymbol{\tau}_{12,0}, \rho_{12,0}\right)}{\partial \rho_{1,2}} \delta_{12}^{*} \\ \vdots \\ \pi_{m_1 m_2,0}^{(12)} \frac{\partial \log \pi_{m_1 m_2}^{(12)}\left(\boldsymbol{\tau}_{12,0}, \rho_{12,0}\right)}{\partial \rho_{1,2}} \delta_{12}^{*} \\ \vdots \\ \pi_{m_{q-1} m_q,0}^{(q-1,q)} \frac{\partial \log \pi_{m_{q-1} m_q}^{(q-1,q)}\left(\boldsymbol{\tau}_{q-1,q,0}, \rho_{q-1,q,0}\right)}{\partial \rho_{q-1,q}} \delta_{q-1,q}^{*} \end{pmatrix} / \sqrt{n} + O\left(n^{-1}\right), \quad (46)$$

where δ_{gh}^{*} is the local parameter in $\boldsymbol{\delta}^{*}$ corresponding to ρ_{gh}. As we showed in Result 1, the partial derivatives are bounded. Hence, (46) implies that $E_{true}\left(\boldsymbol{y}_n\right) = E_0\left(\boldsymbol{y}_0\right) + O\left(n^{-1/2}\right)$. The entries in $\text{var}_{true}\left(\boldsymbol{y}_n\right)$ are either $\text{var}_{true}\left(\boldsymbol{y}_n^{(gh)}\right)$ or $\text{cov}_{true}\left(\boldsymbol{y}_n^{(gh)}, \boldsymbol{y}_n^{(st)}\right)$ for $g < h$ and $s < t$. Equation (34) is directly applicable to $\text{var}_{true}\left(\boldsymbol{y}_n^{(gh)}\right)$, yielding $\text{var}_{true}\left(\boldsymbol{y}_n^{(gh)}\right) = \text{var}_0\left(\boldsymbol{y}_0^{(gh)}\right) + O\left(n^{-1/2}\right)$. For $\text{cov}_{true}\left(\boldsymbol{y}_n^{(gh)}, \boldsymbol{y}_n^{(st)}\right)$, note that the entries in $E_{true}\left[\boldsymbol{y}_n^{(gh)}\left(\boldsymbol{y}_n^{(st)}\right)^T\right]$ are of the form $E_{true}\left[I\left\{x_g = a \text{ and } x_h = b\right\} I\left\{x_s = c \text{ and } x_t = d\right\}\right]$, which can be 0, or $\pi_{a_1 a_2, true}^{(g_1 g_2)}$, or $\pi_{a_1 a_2 a_3, true}^{(g_1 g_2 g_3)}$, or $\pi_{a_1 a_2 a_3 a_4, true}^{(g_1 g_2 g_3 g_4)}$. By (8), we get

$$\begin{aligned} & E_{true}\left[I\left\{x_g = a \text{ and } x_h = b\right\} I\left\{x_s = c \text{ and } x_t = d\right\}\right] \\ = \; & E_0\left[I\left\{x_g = a \text{ and } x_h = b\right\} I\left\{x_s = c \text{ and } x_t = d\right\}\right] + O\left(n^{-1/2}\right), \end{aligned}$$

and $E_{true}\left[\boldsymbol{y}_n^{(gh)}\left(\boldsymbol{y}_n^{(st)}\right)^T\right] = E_0\left[\boldsymbol{y}_n^{(gh)}\left(\boldsymbol{y}_n^{(st)}\right)^T\right] + O\left(n^{-1/2}\right)$. Hence,

$$\begin{aligned} \text{cov}_{true}\left(\boldsymbol{y}_n^{(gh)}, \boldsymbol{y}_n^{(st)}\right) &= E_{true}\left[\boldsymbol{y}_n^{(gh)}\left(\boldsymbol{y}_n^{(st)}\right)^T\right] - E_{true}\left(\boldsymbol{y}_n^{(gh)}\right)\left[E_{true}\left(\boldsymbol{y}_n^{(st)}\right)\right]^T \\ &= E_0\left[\boldsymbol{y}_n^{(gh)}\left(\boldsymbol{y}_n^{(st)}\right)^T\right] - E_0\left(\boldsymbol{y}_0^{(gh)}\right)\left[E_0\left(\boldsymbol{y}_0^{(st)}\right)\right]^T + O\left(n^{-1/2}\right). \end{aligned}$$

Therefore,

$$\text{var}_{true}\left(\boldsymbol{y}_n\right) = \text{var}_0\left(\boldsymbol{y}_0\right) + O\left(n^{-1/2}\right), \quad (47)$$

which is a generalization of (34) to the multivariate case.

To apply the Lindeberg-Feller theorem, we consider $\boldsymbol{y}_n - E_{true}(\boldsymbol{y}_n)$, which has mean $\mathbf{0}$. Since $\left\| \boldsymbol{y}_n^{(gh)} - E_{true}\left(\boldsymbol{y}_n^{(gh)}\right) \right\|^2 \leq 2$ for any pair (g,h), then

$$\left\| \frac{\boldsymbol{y}_{n,i} - E_{true}(\boldsymbol{y}_{n,i})}{\sqrt{n}} \right\|^2 \leq \frac{q(q-1)}{n}.$$

Hence, the Lindeberg condition holds since, for every $\epsilon > 0$, $\|\boldsymbol{y}_{n,i} - E_{true}(\boldsymbol{y}_{n,i})\|^2 < n\epsilon^2$ and

$$\sum_{i=1}^{n} E\left[\left\| \frac{\boldsymbol{y}_{n,i} - E_{true}(\boldsymbol{y}_{n,i})}{\sqrt{n}} \right\|^2 I\left\{ \left\| \frac{\boldsymbol{y}_{n,i} - E_{true}(\boldsymbol{y}_{n,i})}{\sqrt{n}} \right\| \geq \epsilon \right\} \right] = 0$$

for a sufficiently large n. Note that

$$\sum_{i=1}^{n} \mathrm{var}_{true}\left(\frac{\boldsymbol{y}_{n,i} - E_{true}(\boldsymbol{y}_{n,i})}{\sqrt{n}} \right) = \mathrm{var}_{true}(\boldsymbol{y}_{n,i}) \to \mathrm{var}_0(\boldsymbol{y}_0)$$

by (47). Therefore, the central limit theorem implies

$$\frac{1}{\sqrt{n}} \sum_{i=1}^{n} [\boldsymbol{y}_{n,i} - E_{true}(\boldsymbol{y}_{n,i})] \xrightarrow{d} N(\mathbf{0}, \mathrm{var}_0(\boldsymbol{y}_0)).$$

By (46), we further get

$$\sqrt{n}\,[\boldsymbol{p} - E_0(\boldsymbol{y}_0)] \xrightarrow{d} N\left(\begin{pmatrix} \pi_{11,0}^{(12)} \frac{\partial \log \pi_{11}^{(12)}(\boldsymbol{\tau}_{12,0}, \rho_{12,0})}{\partial \rho_{1,2}} \delta_{12}^{*} \\ \vdots \\ \pi_{m_1 m_2,0}^{(12)} \frac{\partial \log \pi_{m_1 m_2}^{(12)}(\boldsymbol{\tau}_{12,0}, \rho_{12,0})}{\partial \rho_{1,2}} \delta_{12}^{*} \\ \vdots \\ \pi_{m_{q-1} m_q,0}^{(q-1,q)} \frac{\partial \log \pi_{m_{q-1} m_q}^{(q-1,q)}(\boldsymbol{\tau}_{q-1,q,0}, \rho_{q-1,q,0})}{\partial \rho_{q-1,q}} \delta_{q-1,q}^{*} \end{pmatrix}, \mathrm{var}_0(\boldsymbol{y}_0) \right),$$

where the asymptotic covariance matrix $\mathrm{var}_0(\boldsymbol{y}_0)$ is evaluated at $(\boldsymbol{\tau}_0, \boldsymbol{\rho}_0)$, rather than $(\boldsymbol{\tau}_0, \boldsymbol{\rho}_{true})$. Hence, (43) implies that

$$\sqrt{n}\,(\hat{\boldsymbol{\rho}} - \boldsymbol{\rho}_0) \xrightarrow{d} N\left(\boldsymbol{H}_0^{-1} \boldsymbol{E}_0 \begin{pmatrix} \pi_{11,0}^{(12)} \frac{\partial \log \pi_{11}^{(12)}(\boldsymbol{\tau}_{12,0}, \rho_{12,0})}{\partial \rho_{1,2}} \delta_{12}^{*} \\ \vdots \\ \pi_{m_1 m_2,0}^{(12)} \frac{\partial \log \pi_{m_1 m_2}^{(12)}(\boldsymbol{\tau}_{12,0}, \rho_{12,0})}{\partial \rho_{1,2}} \delta_{12}^{*} \\ \vdots \\ \pi_{m_{q-1} m_q,0}^{(q-1,q)} \frac{\partial \log \pi_{m_{q-1} m_q}^{(q-1,q)}(\boldsymbol{\tau}_{q-1,q,0}, \rho_{q-1,q,0})}{\partial \rho_{q-1,q}} \delta_{q-1,q}^{*} \end{pmatrix}, \boldsymbol{H}_0^{-1} \boldsymbol{E}_0 \mathrm{var}_0(\boldsymbol{y}_0) \boldsymbol{E}_0^T \boldsymbol{H}_0^{-1} \right).$$

As we have showed for the univariate case,

$$\frac{\mathrm{vec}^T\left(\boldsymbol{\Gamma}^{(gh)}\right)\mathrm{diag}\left(\mathrm{vec}\left(\boldsymbol{\pi}_0^{(gh)}\right)\right)}{-E_0\left(\frac{\partial^2\ell_i\left(\boldsymbol{\tau}_0^{(gh)},\rho_{gh,0}\right)}{\partial\rho^2}\right)}\frac{\partial\log\left\{\mathrm{vec}\left[\boldsymbol{\pi}^{(gh)}\left(\boldsymbol{\tau}_0^{(gh)},\rho_{gh,0}\right)\right]\right\}}{\partial\rho}\delta_{gh}^* = \delta_{gh}^*,$$

for all g and h. Thus,

$$\sqrt{n}\left(\hat{\boldsymbol{\rho}}-\boldsymbol{\rho}_0\right) \overset{d}{\to} N\left(\boldsymbol{\delta}^*, \boldsymbol{H}_0^{-1}\boldsymbol{E}_0\mathrm{var}_0\left(\boldsymbol{y}_0\right)\boldsymbol{H}_0^{-1}\boldsymbol{E}_0^T\right)$$

and

$$\sqrt{n}\left(\hat{\boldsymbol{\rho}}-\boldsymbol{\rho}_{true}\right) \overset{d}{\to} N\left(\mathbf{0}, \boldsymbol{H}_0^{-1}\boldsymbol{E}_0\mathrm{var}_0\left(\boldsymbol{y}_0\right)\boldsymbol{H}_0^{-1}\boldsymbol{E}_0^T\right)$$

This means that the local asymptotic framework affects asymptotic mean but the asymptotic covariance matrix remains the same as the one under the standard asymptotic framework. The proof for the multivariate case is completed.

□

2.5 Simulation Study

A simulation is conducted to investigate the property of $\hat{\boldsymbol{\rho}}$ under the local asymptotic framework. Theorem 1 means that $\hat{\boldsymbol{\rho}}$ is a consistent estimator of $\boldsymbol{\rho}_0$ also under the local asymptotic framework. Theorem 2 means that the estimated asymptotic covariance matrix from standard SEM package remains valid under the local asymptotic framework. However, $\sqrt{n}\left(\hat{\boldsymbol{\rho}}-\boldsymbol{\rho}_{true}\right) \overset{d}{\to} N\left(\mathbf{0},\boldsymbol{\Upsilon}\right)$ does not necessarily imply $\lim_{n\to\infty} E\left[\sqrt{n}\left(\hat{\boldsymbol{\rho}}-\boldsymbol{\rho}_{true}\right)\right]=\mathbf{0}$. Consider the function $\boldsymbol{h}\left(\boldsymbol{x}\right)=\begin{pmatrix}h_1\left(x_1\right) & \cdots & h_p\left(x_p\right)\end{pmatrix}$, where $\boldsymbol{x}$ is a $p\times 1$ vector,

$$h_i\left(x_i\right) = \begin{cases} x_i, & |x_i|\le M,\\ M, & |x_i|>M,\end{cases}$$

and M is a fixed constant. It is easy to see that $\boldsymbol{h}\left(\boldsymbol{x}\right)$ is a continuous and bounded function. Then, for a sufficiently large M, $\lim_{n\to\infty} E\left[\boldsymbol{h}\left\{\sqrt{n}\left(\hat{\boldsymbol{\rho}}-\boldsymbol{\rho}_{true}\right)\right\}\right]$ is approximately $\mathbf{0}$ and $\lim_{n\to\infty} E\left[\boldsymbol{h}\left\{\sqrt{n}\left(\hat{\boldsymbol{\rho}}-\boldsymbol{\rho}_0\right)\right\}\right]$ is approximately $\boldsymbol{h}\left(\boldsymbol{\delta}^*\right)$ by Jiang (2010, pp 45).

Continuous data are generated from the multivariate normal distribution with mean $\mathbf{0}$ and covariance matrix

$$\begin{pmatrix} 1 & 0.4+\delta^* n^{-1/2} & 0.3+\delta^* n^{-1/2} \\ 0.4+\delta^* n^{-1/2} & 1 & 0.35+\delta^* n^{-1/2} \\ 0.3+\delta^* n^{-1/2} & 0.35+\delta^* n^{-1/2} & 1 \end{pmatrix}.$$

Hence, there are three correlation coefficients and six estimates of asymptotic covariances/variances. We consider five different δ^* values, namely, $\delta^* = 0$, 0.1, 0.2, 0.3, and 0.4. When $\delta^* = 0$, we are simply working with the standard asymptotic framework. The population thresholds are chosen such that the probabilities of belonging to each category are 0.24, 0.41, 0.22, 0.1, and 0.03, which is the moderate asymmetry setting in Rhemtulla et al. (2012). The sample size considered here is $n = 100$, 200, 400, 1000, 5000, and $10,000$. The number of replication is $10,000$. We use the lavaan (Rosseel, 2012) package to estimate the polychoric correlation coefficient and its asymptotic covariance/variance.

Figure 1 illustrates the estimated density of $\hat{\rho} - \rho_0$ for each pair of indicators. If Theorem 1 holds, $\hat{\rho} - \rho_0$ will be sufficiently close to 0 as n increases. It is seen that the estimated density becomes more concentrated around 0 as n increases. This implies that $P(|\hat{\rho} - \rho_0| > \epsilon)$ may be sufficiently small for a small ϵ.

As mentioned above, if Theorem 2 holds, $\lim_{n\to\infty} E[h\{\sqrt{n}(\hat{\rho} - \rho_{true})\}] \approx 0$ and $\lim_{n\to\infty} E[h\{\sqrt{n}(\hat{\rho} - \rho_{true})\}] \approx \delta^*$ for all correlation coefficients if M is sufficiently large. In our simulation, the realized $\sqrt{n}(\hat{\rho} - \rho_{true})$ is always bounded between -10 and 10. We can let $M \geq 10$ without changing the sample mean of $\sqrt{n}(\hat{\rho} - \rho_{true})$. Figure 2 illustrates the the bias of the polychoric correlation estimators, where the bias is defined by

$$\frac{1}{R}\sum_{r=1}^{R} h\left\{\sqrt{n}(\hat{\rho}_r - \rho_{true})\right\} \quad \text{or} \quad \frac{1}{R}\sum_{r=1}^{R} h\left\{\sqrt{n}(\hat{\rho}_r - \rho_0)\right\}$$

$\hat{\rho}_r$ is the estimator at iteration r, and R is the number of replications. As expected, it is seen that the $R^{-1}\sum_{r=1}^{R} h\{\sqrt{n}(\hat{\rho}_r - \rho_{true})\}$ remains low, whereas $R^{-1}\sum_{r=1}^{R} h\{\sqrt{n}(\hat{\rho}_r - \rho_0)\}$ is approximately δ^*.

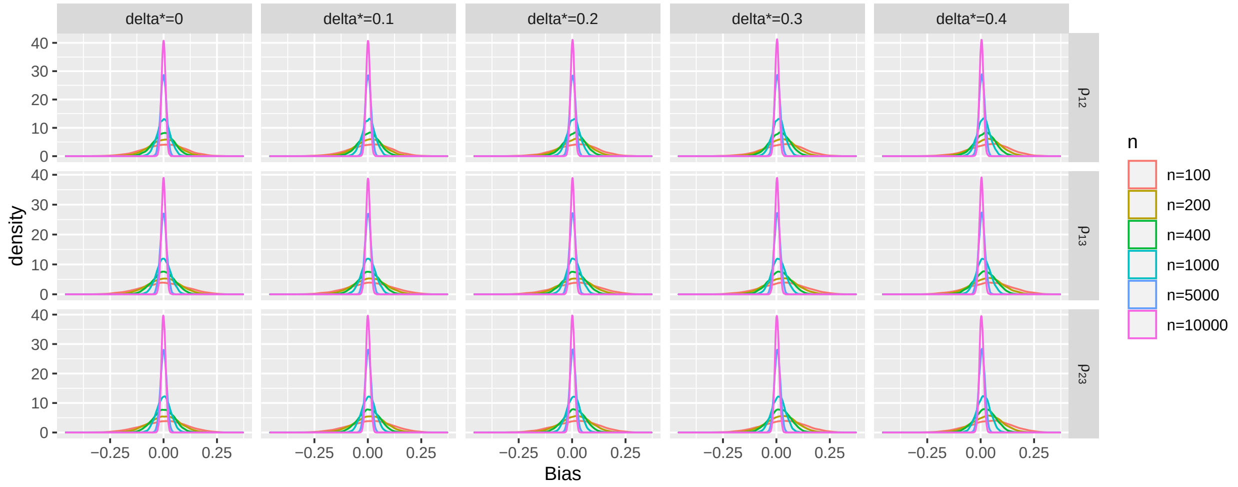

Figure 1. Estimated density of $\hat{\rho} - \rho_0$. Columns correspond to different δ^* values and rows correspond to different correlation coefficients.

Regarding the asymptotic covariance estimator, we do not know the true value. Nevertheless, if Theorem 2 holds, the estimated asymptotic covariance matrix from lavaan should be close to the sample covariance of the estimated correlation coefficients. Figure 3 illustrates the relative bias of asymptotic covariance/variance estimators of polychoric correlation estimators, where the relative bias is defined by

$$100 \cdot \frac{1}{R}\sum_{r=1}^{R}\frac{\hat{\Upsilon}_r - \tilde{\Upsilon}_0}{\tilde{\Upsilon}_0},$$

where $\hat{\Upsilon}_r$ is the estimate of Υ_0 at iteration r, and $\tilde{\Upsilon}_0$ is the sample covariance of the estimated correlation coefficients. It is seen from Figure 3 that the relative bias of the asymptotic covariance/variance estimators is generally small, as the sample size increases.

3 Frequentist Model Averaging

In the context of SEM with normally distributed continuous data, Jin and Ankargren (2019) derived the asymptotic distribution of $\sqrt{n}\begin{pmatrix}\hat{\boldsymbol{\theta}}_s - \boldsymbol{\theta}_0 \\ \hat{\boldsymbol{\gamma}}_s - \boldsymbol{\gamma}_{0,s}\end{pmatrix}$ in their equation (6) and the asymptotic distribution of $\sqrt{n}\,(\bar{\boldsymbol{\mu}} - \boldsymbol{\mu}_{true})$ in their equation (9). For the purpose of presentation, their results are placed in the following lemma.

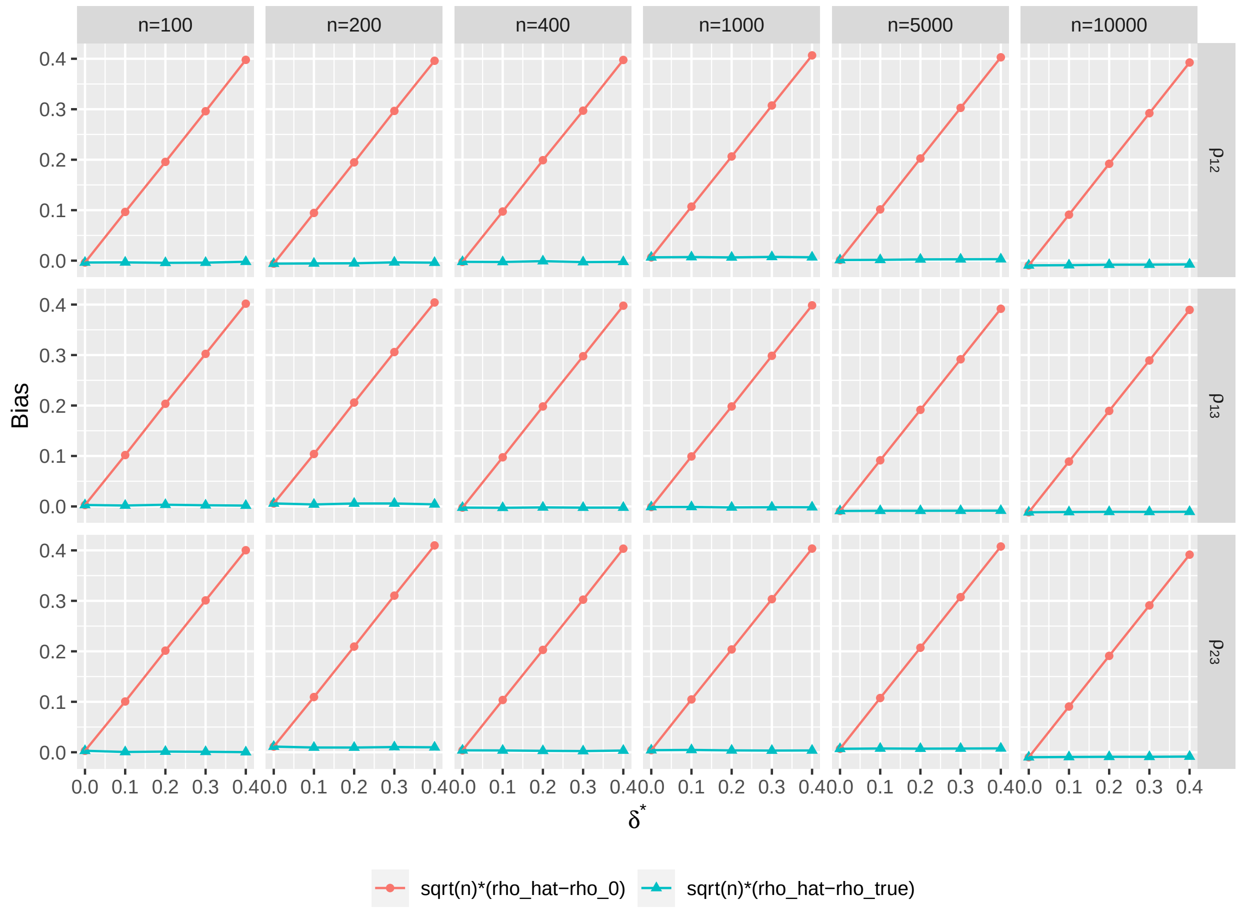

Figure 2. Bias of polychoric correlation estimators. Different colors correspond to the bias of estimating ρ_0 or ρ_{true}.

Lemma 1. *Suppose that $\boldsymbol{J}_s$ is invertible and all third-order partial derivatives of the fit function $F(\boldsymbol{\theta}_0, \boldsymbol{\gamma}_{0,s}, \boldsymbol{\gamma}_{0,s^c})$ in a neighborhood of $(\boldsymbol{\theta}_0, \boldsymbol{\gamma}_0)$ are dominated by functions with finite means. Then,*

$$\sqrt{n}\begin{pmatrix}\hat{\boldsymbol{\theta}}_s - \boldsymbol{\theta}_0 \\ \hat{\boldsymbol{\gamma}}_s - \boldsymbol{\gamma}_{0,s}\end{pmatrix} = \boldsymbol{J}_s^{-1}\left[\begin{pmatrix}\boldsymbol{J}_{\theta\gamma} \\ \boldsymbol{\pi}_s \boldsymbol{J}_{\gamma\gamma}\end{pmatrix}\boldsymbol{\delta} + \begin{pmatrix}\boldsymbol{M} \\ \boldsymbol{\pi}_s \boldsymbol{N}\end{pmatrix}\right] + O_P\left(n^{-1/2}\right). \tag{48}$$

and

$$\begin{aligned}\sqrt{n}\left(\bar{\boldsymbol{\mu}} - \boldsymbol{\mu}_{true}\right) &= \frac{\partial \boldsymbol{\mu}_0}{\partial \boldsymbol{\theta}^T}\sum_c c_s \sqrt{n}\left(\hat{\boldsymbol{\theta}}_s - \boldsymbol{\theta}_0\right) + \sum_c c_s \frac{\partial \boldsymbol{\mu}_0}{\partial \boldsymbol{\gamma}_s^T}\sqrt{n}\left(\hat{\boldsymbol{\gamma}}_s - \boldsymbol{\gamma}_{0,s}\right) - \frac{\partial \boldsymbol{\mu}_0}{\partial \boldsymbol{\gamma}^T}\boldsymbol{\delta} + O_P\left(n^{-1/2}\right) \\ &= \frac{\partial \boldsymbol{\mu}_0}{\partial \boldsymbol{\theta}^T}\boldsymbol{J}_{\theta\theta}^{-1}\boldsymbol{M} + \boldsymbol{W}\left\{\boldsymbol{\delta} - \left(\sum_s c_s \boldsymbol{K}^{(s)}\right)\boldsymbol{K}^{-1}\boldsymbol{D}\right\} + O_P\left(n^{-1/2}\right), \end{aligned} \tag{49}$$

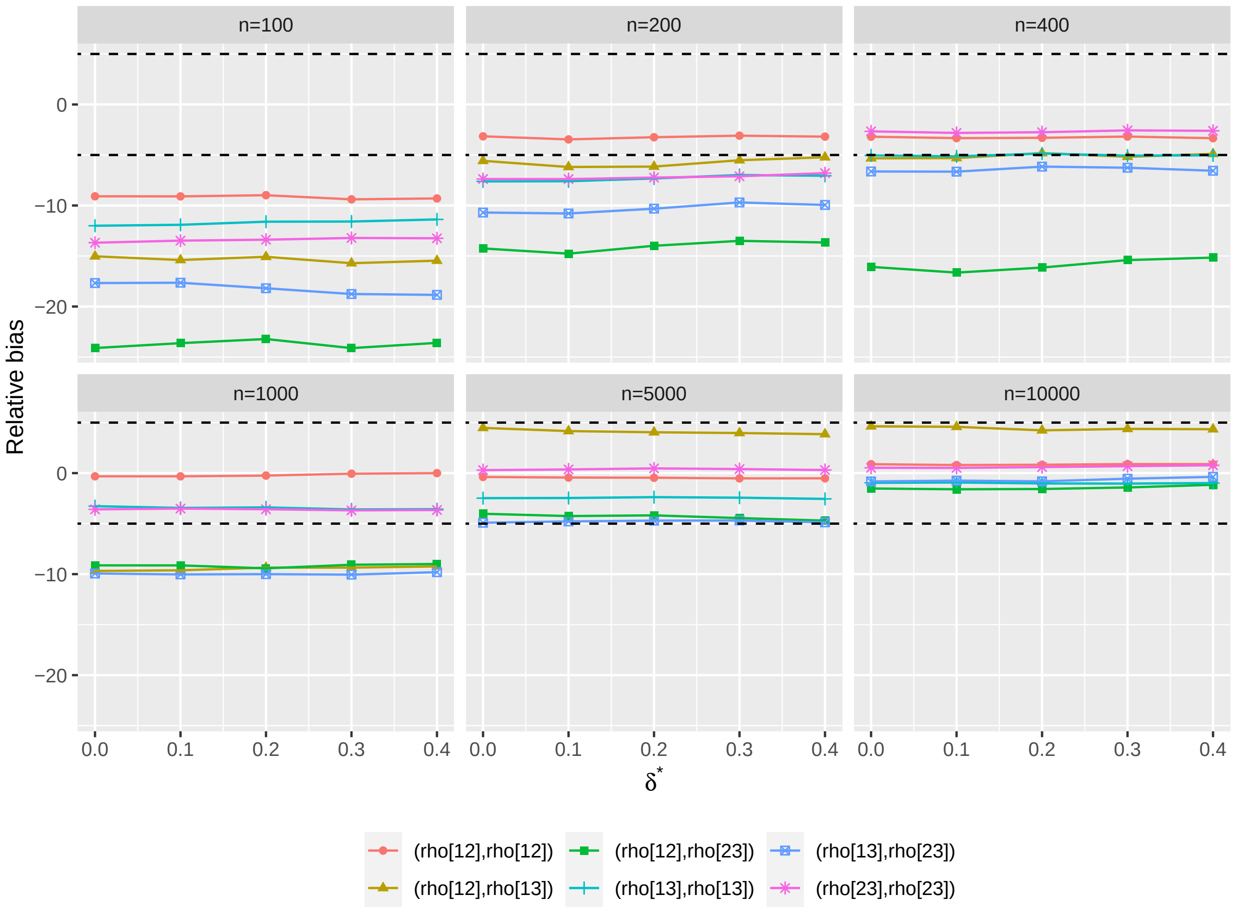

Figure 3. Relative bias of asymptotic covariance/variance estimators of polychoric correlation estimators. Different colors correspond to the asymptotic covariance between estimators of different correlation coefficients.

where $\boldsymbol{\mu}_{true} = \boldsymbol{\mu}\left(\boldsymbol{\theta}_0, \boldsymbol{\gamma}_0 + \boldsymbol{\delta}/\sqrt{n}\right)$, $\boldsymbol{\mu}_0 = \boldsymbol{\mu}\left(\boldsymbol{\theta}_0, \boldsymbol{\gamma}_0\right)$, $\boldsymbol{W} = \frac{\partial \boldsymbol{\mu}_0}{\partial \boldsymbol{\theta}^T} \boldsymbol{J}_{\theta\theta}^{-1} \boldsymbol{J}_{\theta\gamma} - \frac{\partial \boldsymbol{\mu}_0}{\partial \boldsymbol{\gamma}^T}$,
$\boldsymbol{K}^{-1} = \boldsymbol{J}_{\gamma\gamma} - \boldsymbol{J}_{\theta\gamma}^T \boldsymbol{J}_{\theta\theta}^{-1} \boldsymbol{J}_{\theta\gamma}$,$\boldsymbol{K}_s = \left(\boldsymbol{\pi}_s \boldsymbol{K}^{-1} \boldsymbol{\pi}_s^T\right)^{-1}$, $\boldsymbol{K}^{(s)} = \boldsymbol{\pi}_s^T \boldsymbol{K}_s \boldsymbol{\pi}_s$,
$\boldsymbol{D} = \boldsymbol{\delta} - \boldsymbol{K}\boldsymbol{J}_{\theta\gamma}^T \boldsymbol{J}_{\theta\theta}^{-1} \boldsymbol{M} + \boldsymbol{K}\boldsymbol{N}$, *and*

$$-\frac{1}{2\sqrt{n}} \frac{\partial F\left(\boldsymbol{\beta}_0\right)}{\partial \boldsymbol{\beta}} - \begin{pmatrix} \boldsymbol{J}_{\theta\gamma} \\ \boldsymbol{J}_{\gamma\gamma} \end{pmatrix} \boldsymbol{\delta} \xrightarrow{d} \begin{pmatrix} \boldsymbol{M} \\ \boldsymbol{N} \end{pmatrix}.$$

(Jin and Ankargren, 2019).

The fit function F used in Jin and Ankargren (2019) is the maximum likelihood fit function that will be denoted by F_{ML}. When the observed indicators are ordinal, the least

squares fit function is commonly used, which will be denoted by F_{LS}. Since both F_{ML} and F_{LS} can be viewed as distance functions between $\boldsymbol{\rho}$ and $\boldsymbol{\sigma}$, Lemma 1 remains valid for ordinal SEM, if $F = F_{LS}$ and Assumptions A1 to A5 also hold. Hereafter, we assume all these assumptions hold.

We first derive the joint distribution of $\boldsymbol{M}$ and $\boldsymbol{N}$ under the assumptions in Theorem 2. Here $\boldsymbol{M}$ and $\boldsymbol{N}$ are defined by

$$-\frac{1}{2\sqrt{n}}\frac{\partial F_{LS}\left(\boldsymbol{\beta}_0\right)}{\partial \boldsymbol{\beta}} - \begin{pmatrix} \boldsymbol{J}_{\theta\gamma} \\ \boldsymbol{J}_{\gamma\gamma} \end{pmatrix} \boldsymbol{\delta} \ \overset{d}{\to} \ \begin{pmatrix} \boldsymbol{M} \\ \boldsymbol{N} \end{pmatrix},$$

simply replacing F by F_{LS} in all partial derivatives.

Proof of joint distribution of $\boldsymbol{M}$ and $\boldsymbol{N}$. Note that

$$\frac{\partial F_{LS}\left(\boldsymbol{\beta}\right)}{\partial \boldsymbol{\beta}} = -2n\left(\frac{\partial \boldsymbol{\sigma}\left(\boldsymbol{\beta}\right)}{\partial \boldsymbol{\beta}^T}\right)^T \hat{\boldsymbol{V}}\left(\hat{\boldsymbol{\rho}} - \boldsymbol{\sigma}\left(\boldsymbol{\beta}\right)\right),$$

where $\hat{\boldsymbol{V}}$ is a consistent estimator of $\boldsymbol{V}$. By Theorem 1, $\hat{\boldsymbol{\rho}}$ is a consistent estimator of $\boldsymbol{\sigma}_0 = \boldsymbol{\sigma}\left(\boldsymbol{\beta}_0\right)$. Then,

$$\frac{1}{n}\frac{\partial^2 F\left(\boldsymbol{\beta}_0\right)}{\partial \boldsymbol{\beta}\partial \boldsymbol{\beta}^T} \ \overset{p}{\to} \ 2\boldsymbol{J}_{full},$$

where

$$\boldsymbol{J}_{full} = \left(\frac{\partial \boldsymbol{\sigma}_0}{\partial \boldsymbol{\beta}^T}\right)^T \boldsymbol{V}\frac{\partial \boldsymbol{\sigma}_0}{\partial \boldsymbol{\beta}^T}.$$

Hence,

$$\begin{aligned}
-\frac{1}{2\sqrt{n}}\frac{\partial F_{LS}\left(\boldsymbol{\beta}_0\right)}{\partial \boldsymbol{\beta}} - \begin{pmatrix} \boldsymbol{J}_{\theta\gamma} \\ \boldsymbol{J}_{\gamma\gamma} \end{pmatrix} \boldsymbol{\delta} &= \sqrt{n}\left(\frac{\partial \boldsymbol{\sigma}_0}{\partial \boldsymbol{\beta}^T}\right)^T \hat{\boldsymbol{V}}\left(\hat{\boldsymbol{\rho}} - \boldsymbol{\sigma}_0\right) - \begin{pmatrix} \boldsymbol{J}_{\theta\gamma} \\ \boldsymbol{J}_{\gamma\gamma} \end{pmatrix} \boldsymbol{\delta} \\
&= \begin{pmatrix} \left(\frac{\partial \boldsymbol{\sigma}_0}{\partial \boldsymbol{\theta}^T}\right)^T \hat{\boldsymbol{V}}\sqrt{n}\left(\hat{\boldsymbol{\rho}} - \boldsymbol{\sigma}_0 - \frac{\partial \boldsymbol{\sigma}_0}{\partial \gamma^T}\boldsymbol{\delta}/\sqrt{n}\right) \\ \left(\frac{\partial \boldsymbol{\sigma}_0}{\partial \gamma^T}\right)^T \hat{\boldsymbol{V}}\sqrt{n}\left(\hat{\boldsymbol{\rho}} - \boldsymbol{\sigma}_0 - \frac{\partial \boldsymbol{\sigma}_0}{\partial \gamma^T}\boldsymbol{\delta}/\sqrt{n}\right) \end{pmatrix} \\
&= \begin{pmatrix} \left(\frac{\partial \boldsymbol{\sigma}_0}{\partial \boldsymbol{\theta}^T}\right)^T \boldsymbol{V}\sqrt{n}\left(\hat{\boldsymbol{\rho}} - \boldsymbol{\sigma}_{true}\right) \\ \left(\frac{\partial \boldsymbol{\sigma}_0}{\partial \gamma^T}\right)^T \boldsymbol{V}\sqrt{n}\left(\hat{\boldsymbol{\rho}} - \boldsymbol{\sigma}_{true}\right) \end{pmatrix} + o_{\mathrm{P}}\left(1\right),
\end{aligned}$$

where the last equality holds from (3) and Assumption A5. By Theorem 2,

$$\begin{pmatrix} \left(\frac{\partial\boldsymbol{\sigma}_0}{\partial\boldsymbol{\theta}^T}\right)^T \boldsymbol{V}\sqrt{n}\left(\hat{\boldsymbol{\rho}}-\boldsymbol{\sigma}_{true}\right) \\ \left(\frac{\partial\boldsymbol{\sigma}_0}{\partial\gamma^T}\right)^T \boldsymbol{V}\sqrt{n}\left(\hat{\boldsymbol{\rho}}-\boldsymbol{\sigma}_{true}\right) \end{pmatrix} \xrightarrow{d} N\left(\mathbf{0}, \left(\frac{\partial\boldsymbol{\sigma}_0}{\partial\boldsymbol{\beta}^T}\right)^T \boldsymbol{V}\boldsymbol{\Upsilon}\boldsymbol{V}\frac{\partial\boldsymbol{\sigma}_0}{\partial\boldsymbol{\beta}^T}\right).$$

Hence, the joint distribution of $\boldsymbol{M}$ and $\boldsymbol{N}$ is multivariate normal with mean $\mathbf{0}$ and the covariance matrix

$$\left(\frac{\partial\boldsymbol{\sigma}_0}{\partial\boldsymbol{\beta}^T}\right)^T \boldsymbol{V}\boldsymbol{\Upsilon}\boldsymbol{V}\frac{\partial\boldsymbol{\sigma}_0}{\partial\boldsymbol{\beta}^T}.$$

□

Derivation of the qudratic programming $Q(\boldsymbol{c})$. By (49), the mean of the limiting distribution of $\sqrt{n}\left(\bar{\boldsymbol{\mu}}-\boldsymbol{\mu}_{true}\right)$ is $\boldsymbol{\zeta}=\boldsymbol{W}\left\{\boldsymbol{I}-\left(\sum_s c_s\boldsymbol{K}^{(s)}\right)\boldsymbol{K}^{-1}\right\}\boldsymbol{\delta}$ and the corresponding covariance matrix is

$$\begin{aligned} \boldsymbol{\Omega} \;=\; & \frac{\partial\boldsymbol{\mu}_0}{\partial\boldsymbol{\theta}^T}\boldsymbol{J}_{\theta\theta}^{-1}\mathrm{var}\left(\boldsymbol{M}\right)\boldsymbol{J}_{\theta\theta}^{-1}\left(\frac{\partial\boldsymbol{\mu}_0}{\partial\boldsymbol{\theta}^T}\right)^T \\ & +\boldsymbol{W}\left(\sum_s c_s\boldsymbol{K}^{(s)}\right)\boldsymbol{K}^{-1}\mathrm{var}\left(\boldsymbol{D}\right)\boldsymbol{K}^{-1}\left(\sum_s c_s\boldsymbol{K}^{(s)}\right)\boldsymbol{W}^T \\ & -\frac{\partial\boldsymbol{\mu}_0}{\partial\boldsymbol{\theta}^T}\boldsymbol{J}_{\theta\theta}^{-1}\mathrm{cov}\left(\boldsymbol{M},\boldsymbol{D}^T\right)\boldsymbol{K}^{-1}\left(\sum_s c_s\boldsymbol{\pi}_s^T\boldsymbol{K}_s\boldsymbol{\pi}_s\right)\boldsymbol{W}^T \\ & -\boldsymbol{W}\left(\sum_s c_s\boldsymbol{K}^{(s)}\right)\boldsymbol{K}^{-1}\mathrm{cov}\left(\boldsymbol{D},\boldsymbol{M}^T\right)\boldsymbol{J}_{\theta\theta}^{-1}\left(\frac{\partial\boldsymbol{\mu}_0}{\partial\boldsymbol{\theta}^T}\right)^T. \end{aligned}$$

The limit of $nE\left(\bar{\boldsymbol{\mu}}-\boldsymbol{\mu}_{true}\right)^T\left(\bar{\boldsymbol{\mu}}-\boldsymbol{\mu}_{true}\right)$ is then

$$\begin{aligned}
\operatorname{tr}\left\{\boldsymbol{\Omega}+\boldsymbol{\zeta}\boldsymbol{\zeta}^T\right\} &= \operatorname{tr}\left\{\frac{\partial\boldsymbol{\mu}_0}{\partial\boldsymbol{\theta}^T}\boldsymbol{J}_{\theta\theta}^{-1}\text{var}\left(\boldsymbol{M}\right)\boldsymbol{J}_{\theta\theta}^{-1}\left(\frac{\partial\boldsymbol{\mu}_0}{\partial\boldsymbol{\theta}^T}\right)^T\right\} \\
&+\operatorname{tr}\left\{\boldsymbol{W}\left(\sum_s c_s\boldsymbol{K}^{(s)}\right)\boldsymbol{K}^{-1}\text{var}\left(\boldsymbol{D}\right)\boldsymbol{K}^{-1}\left(\sum_s c_s\boldsymbol{K}^{(s)}\right)\boldsymbol{W}^T\right\} \\
&-\operatorname{tr}\left\{\frac{\partial\boldsymbol{\mu}_0}{\partial\boldsymbol{\theta}^T}\boldsymbol{J}_{\theta\theta}^{-1}\text{cov}\left(\boldsymbol{M},\boldsymbol{D}^T\right)\boldsymbol{K}^{-1}\left(\sum_s c_s\boldsymbol{\pi}_s^T\boldsymbol{K}_s\boldsymbol{\pi}_s\right)\boldsymbol{W}^T\right\} \\
&-\operatorname{tr}\left\{\boldsymbol{W}\left(\sum_s c_s\boldsymbol{K}^{(s)}\right)\boldsymbol{K}^{-1}\text{cov}\left(\boldsymbol{D},\boldsymbol{M}^T\right)\boldsymbol{J}_{\theta\theta}^{-1}\left(\frac{\partial\boldsymbol{\mu}_0}{\partial\boldsymbol{\theta}^T}\right)^T\right\} \\
&+\operatorname{tr}\left\{\boldsymbol{W}\left[\boldsymbol{I}-\left(\sum_s c_s\boldsymbol{K}^{(s)}\right)\boldsymbol{K}^{-1}\right]\boldsymbol{\delta}\boldsymbol{\delta}^T\left[\boldsymbol{I}-\left(\sum_s c_s\boldsymbol{K}^{(s)}\right)\boldsymbol{K}^{-1}\right]^T\boldsymbol{W}^T\right\} \\
&= \operatorname{tr}\left\{\frac{\partial\boldsymbol{\mu}_0}{\partial\boldsymbol{\theta}^T}\boldsymbol{J}_{\theta\theta}^{-1}\text{var}\left(\boldsymbol{M}\right)\boldsymbol{J}_{\theta\theta}^{-1}\left(\frac{\partial\boldsymbol{\mu}_0}{\partial\boldsymbol{\theta}^T}\right)^T\right\} \\
&+\operatorname{tr}\left\{\boldsymbol{W}\left[\sum_s\sum_t c_sc_t\boldsymbol{K}^{(s)}\boldsymbol{K}^{-1}\text{var}\left(\boldsymbol{D}\right)\boldsymbol{K}^{-1}\boldsymbol{K}^{(t)}\right]\boldsymbol{W}^T\right\} \\
&-2\operatorname{tr}\left\{\frac{\partial\boldsymbol{\mu}_0}{\partial\boldsymbol{\theta}^T}\boldsymbol{J}_{\theta\theta}^{-1}\text{cov}\left(\boldsymbol{M},\boldsymbol{D}^T\right)\boldsymbol{K}^{-1}\left(\sum_s c_s\boldsymbol{K}^{(s)}\right)\boldsymbol{W}^T\right\} \\
&+\operatorname{tr}\left\{\boldsymbol{W}\left[\boldsymbol{\delta}\boldsymbol{\delta}^T-2\left(\sum_s c_s\boldsymbol{K}^{(s)}\right)\boldsymbol{K}^{-1}\boldsymbol{\delta}\boldsymbol{\delta}^T\right]\boldsymbol{W}^T\right\} \\
&+\operatorname{tr}\left\{\boldsymbol{W}\left(\sum_s c_s\boldsymbol{K}^{(s)}\right)\boldsymbol{K}^{-1}\boldsymbol{\delta}\boldsymbol{\delta}^T\boldsymbol{K}^{-1}\left(\sum_t c_t\boldsymbol{K}^{(t)}\right)\boldsymbol{W}^T\right\},
\end{aligned}$$

which can be simplified to

$$\begin{aligned}
&\operatorname{tr}\left\{\frac{\partial\boldsymbol{\mu}_0}{\partial\boldsymbol{\theta}^T}\boldsymbol{J}_{\theta\theta}^{-1}\text{var}\left(\boldsymbol{M}\right)\boldsymbol{J}_{\theta\theta}^{-1}\left(\frac{\partial\boldsymbol{\mu}_0}{\partial\boldsymbol{\theta}^T}\right)^T+\boldsymbol{W}\boldsymbol{\delta}\boldsymbol{\delta}^T\boldsymbol{W}^T\right\} \\
&+2\sum_s c_s\operatorname{tr}\left\{-\boldsymbol{W}\boldsymbol{K}^{(s)}\boldsymbol{K}^{-1}\boldsymbol{\delta}\boldsymbol{\delta}^T\boldsymbol{W}^T-\frac{\partial\boldsymbol{\mu}_0}{\partial\boldsymbol{\theta}^T}\boldsymbol{J}_{\theta\theta}^{-1}\text{cov}\left(\boldsymbol{M},\boldsymbol{D}^T\right)\boldsymbol{K}^{-1}\boldsymbol{K}^{(s)}\boldsymbol{W}^T\right\} \\
&+\sum_s\sum_t c_sc_t\operatorname{tr}\left\{\boldsymbol{W}\left[\boldsymbol{K}^{(s)}\boldsymbol{K}^{-1}\left(\text{var}\left(\boldsymbol{D}\right)+\boldsymbol{\delta}\boldsymbol{\delta}^T\right)\boldsymbol{K}^{-1}\boldsymbol{K}^{(t)}\right]\boldsymbol{W}^T\right\}.
\end{aligned}$$

Recall that $\boldsymbol{D}=\boldsymbol{\delta}-\boldsymbol{K}\boldsymbol{J}_{\theta\gamma}^T\boldsymbol{J}_{\theta\theta}^{-1}\boldsymbol{M}+\boldsymbol{K}\boldsymbol{N}$, so that

$$\begin{aligned}
\text{cov}\left(\boldsymbol{M},\boldsymbol{D}^T\right) &= \text{cov}\left(\boldsymbol{M},\boldsymbol{N}^T\right)\boldsymbol{K}-\text{var}\left(\boldsymbol{M}\right)\boldsymbol{J}_{\theta\theta}^{-1}\boldsymbol{J}_{\theta\gamma}\boldsymbol{K} \\
\text{var}\left(\boldsymbol{D}\right) &= \boldsymbol{K}\text{var}\left(\boldsymbol{N}\right)\boldsymbol{K}+\boldsymbol{K}\boldsymbol{J}_{\theta\gamma}^T\boldsymbol{J}_{\theta\theta}^{-1}\text{var}\left(\boldsymbol{M}\right)\boldsymbol{J}_{\theta\theta}^{-1}\boldsymbol{J}_{\theta\gamma}\boldsymbol{K} \\
&-\boldsymbol{K}\text{cov}\left(\boldsymbol{N},\boldsymbol{M}^T\right)\boldsymbol{J}_{\theta\theta}^{-1}\boldsymbol{J}_{\theta\gamma}\boldsymbol{K}-\boldsymbol{K}\boldsymbol{J}_{\theta\gamma}^T\boldsymbol{J}_{\theta\theta}^{-1}\text{cov}\left(\boldsymbol{M},\boldsymbol{N}^T\right)\boldsymbol{K}.
\end{aligned}$$

Hence,

$$\begin{aligned}\operatorname{tr}\left\{\boldsymbol{\Omega}+\boldsymbol{\zeta}\boldsymbol{\zeta}^T\right\} &= \operatorname{tr}\left\{\frac{\partial\boldsymbol{\mu}_0}{\partial\boldsymbol{\theta}^T}\boldsymbol{J}_{\theta\theta}^{-1}\operatorname{var}\left(\boldsymbol{M}\right)\boldsymbol{J}_{\theta\theta}^{-1}\left(\frac{\partial\boldsymbol{\mu}_0}{\partial\boldsymbol{\theta}^T}\right)^T+\boldsymbol{W}\boldsymbol{\delta}\boldsymbol{\delta}^T\boldsymbol{W}^T\right\}\\ &\quad +2\sum_s c_s\operatorname{tr}\left\{\boldsymbol{\Delta}_1\boldsymbol{K}^{(s)}\boldsymbol{W}^T\right\}\\ &\quad +\sum_s\sum_t c_sc_t\operatorname{tr}\left\{\boldsymbol{W}\left[\boldsymbol{K}^{(s)}\boldsymbol{\Delta}_2\boldsymbol{K}^{(t)}\right]\boldsymbol{W}^T\right\},\end{aligned}$$

where

$$\begin{aligned}\boldsymbol{\Delta}_1 =&\frac{\partial\boldsymbol{\mu}_0}{\partial\boldsymbol{\theta}^T}\boldsymbol{J}_{\theta\theta}^{-1}\left(Var\left(\boldsymbol{M}\right)\boldsymbol{J}_{\theta\theta}^{-1}\boldsymbol{J}_{\theta\gamma}-Cov\left(\boldsymbol{M},\boldsymbol{N}^T\right)\right)-\boldsymbol{W}\boldsymbol{\delta}\boldsymbol{\delta}^T\boldsymbol{K}^{-1},\\ \boldsymbol{\Delta}_2 =&\boldsymbol{J}_{\theta\gamma}^T\boldsymbol{J}_{\theta\theta}^{-1}Var\left(\boldsymbol{M}\right)\boldsymbol{J}_{\theta\theta}^{-1}\boldsymbol{J}_{\theta\gamma}+Var\left(\boldsymbol{N}\right)-2\boldsymbol{J}_{\theta\gamma}^T\boldsymbol{J}_{\theta\theta}^{-1}Cov\left(\boldsymbol{M},\boldsymbol{N}^T\right)+\boldsymbol{K}^{-1}\boldsymbol{\delta}\boldsymbol{\delta}^T\boldsymbol{K}^{-1}.\end{aligned}$$

The first term in $\operatorname{tr}\left\{\boldsymbol{\Omega}+\boldsymbol{\zeta}\boldsymbol{\zeta}^T\right\}$ does not depend on the weights. Hence, minimizing $\operatorname{tr}\left\{\boldsymbol{\Omega}+\boldsymbol{\zeta}\boldsymbol{\zeta}^T\right\}$ is equivalent to minimizing

$$Q\left(\boldsymbol{c}\right)=\sum_s c_s tr\left\{\boldsymbol{\Delta}_1\boldsymbol{K}^{(s)}\boldsymbol{W}^T\right\}+\frac{1}{2}\sum_s\sum_t c_sc_t tr\left\{\boldsymbol{W}\boldsymbol{K}^{(s)}\boldsymbol{\Delta}_2\boldsymbol{K}^{(t)}\boldsymbol{W}^T\right\}. \tag{50}$$

The proof is completed.

□

Unbiased estimator of $\boldsymbol{\delta}$ *and* $\boldsymbol{\delta}\boldsymbol{\delta}^T$. Using the inverse of a 2 × 2 block matrix,

$$\boldsymbol{J}_s^{-1} = \begin{pmatrix}\boldsymbol{J}_{\theta\theta}^{-1}+\boldsymbol{J}_{\theta\theta}^{-1}\boldsymbol{J}_{\theta\gamma}\boldsymbol{\pi}_s^T\boldsymbol{K}_s\boldsymbol{\pi}_s\boldsymbol{J}_{\theta\gamma}^T\boldsymbol{J}_{\theta\theta}^{-1} & -\boldsymbol{J}_{\theta\theta}^{-1}\boldsymbol{J}_{\theta\gamma}\boldsymbol{\pi}_s^T\boldsymbol{K}_s\\ -\boldsymbol{K}_s\boldsymbol{\pi}_s\boldsymbol{J}_{\theta\gamma}^T\boldsymbol{J}_{\theta\theta}^{-1} & \boldsymbol{K}_s\end{pmatrix}.$$

Then, equation (48) implies that

$$\hat{\boldsymbol{\delta}}=\sqrt{n}\left(\hat{\boldsymbol{\gamma}}_{full}-\boldsymbol{\gamma}_0\right)\ \overset{d}{\to}\ \boldsymbol{\delta}-\boldsymbol{K}\boldsymbol{J}_{\theta\gamma}^T\boldsymbol{J}_{\theta\theta}^{-1}\boldsymbol{M}+\boldsymbol{K}\boldsymbol{N}\sim N\left(\boldsymbol{\delta},\ \boldsymbol{G}\boldsymbol{H}\boldsymbol{G}^T\right),$$

where $\boldsymbol{G}=\begin{pmatrix}-\boldsymbol{K}\boldsymbol{J}_{\theta\gamma}^T\boldsymbol{J}_{\theta\theta}^{-1} & \boldsymbol{K}\end{pmatrix}$ and

$$\boldsymbol{H} = \operatorname{var}\begin{pmatrix}\boldsymbol{M}\\ \boldsymbol{N}\end{pmatrix}=\left(\frac{\partial\boldsymbol{\sigma}_0}{\partial\boldsymbol{\beta}^T}\right)^T\boldsymbol{V}\boldsymbol{\Upsilon}\boldsymbol{V}\frac{\partial\boldsymbol{\sigma}_0}{\partial\boldsymbol{\beta}^T}.$$

Hence, the mean of the asymptotic distribution of $\hat{\boldsymbol{\delta}}$ is still $\boldsymbol{\delta}$. The mean of the asymptotic distribution of $\hat{\boldsymbol{\delta}}\hat{\boldsymbol{\delta}}^T$ is $\boldsymbol{\delta}\boldsymbol{\delta}^T+\boldsymbol{G}\boldsymbol{H}\boldsymbol{G}^T$. An asymptotically unbiased estimator of $\boldsymbol{\delta}\boldsymbol{\delta}^T$ is then $\hat{\boldsymbol{\delta}}\hat{\boldsymbol{\delta}}^T-\hat{\boldsymbol{G}}\hat{\boldsymbol{H}}\hat{\boldsymbol{G}}$.

□

Heuristic proof of the joint convergence. In practice, $\boldsymbol{c}$ is estimated by maximizing

$$\hat{Q}(\boldsymbol{c}) = \sum_s c_s \mathrm{tr}\left\{\hat{\boldsymbol{\Delta}}_1 \hat{\boldsymbol{K}}^{(s)} \hat{\boldsymbol{W}}^T\right\} + \frac{1}{2}\sum_s \sum_t c_s c_t \mathrm{tr}\left\{\hat{\boldsymbol{W}} \hat{\boldsymbol{K}}^{(s)} \hat{\boldsymbol{\Delta}}_2 \hat{\boldsymbol{K}}^{(t)} \hat{\boldsymbol{W}}^T\right\},$$

subject to the unit simplex, where $\hat{\boldsymbol{W}}$ and $\hat{\boldsymbol{K}}^{(s)}$ are consistent estimators of $\boldsymbol{W}$ and $\boldsymbol{K}^{(s)}$ respectively. Since $\hat{\boldsymbol{\delta}} = \sqrt{n}\left(\hat{\gamma}_{full} - \gamma_0\right) \xrightarrow{d} \boldsymbol{D}$, $\hat{\boldsymbol{\Delta}}_1$ and $\hat{\boldsymbol{\Delta}}_2$ are not consistent estimators of $\boldsymbol{\Delta}_1$ and $\boldsymbol{\Delta}_2$. They are only asymptotically unbiased. Hence, $\hat{Q}(\boldsymbol{c}) \xrightarrow{d} Q^*(\boldsymbol{c})$ for some $Q^*(\boldsymbol{c})$, of which the distribution depends on the joint distribution of $\boldsymbol{M}$ and $\boldsymbol{N}$. If the quadratic programming is positive definite, then $\boldsymbol{c}^*$, the minimizer of $Q^*(\boldsymbol{c})$, is unique. In the context of linear regression, Liu (2015) showed that

$$\sqrt{n}\left(\bar{\boldsymbol{\mu}}(\hat{\boldsymbol{c}}) - \boldsymbol{\mu}_{true}\right) \xrightarrow{d} \frac{\partial \boldsymbol{\mu}_0}{\partial \boldsymbol{\theta}^T} \boldsymbol{J}_{\theta\theta}^{-1} \boldsymbol{M} + \boldsymbol{W}\left\{\boldsymbol{\delta} - \left(\sum_s c_s^* \boldsymbol{K}^{(s)}\right) \boldsymbol{K}^{-1} \boldsymbol{D}\right\}, \tag{51}$$

where $\hat{\boldsymbol{c}}$ is the minimizer of $\hat{Q}(\boldsymbol{c})$ and $\boldsymbol{c}^*$ is the minimizer of $Q^*(\boldsymbol{c})$. Suppose that $\hat{\boldsymbol{c}} \xrightarrow{d} \boldsymbol{c}^*$. Since the distribution of $Q^*(\boldsymbol{c})$ depends on the joint distribution of $\boldsymbol{M}$ and $\boldsymbol{N}$, the distribution of $\boldsymbol{c}^*$ also depends on $\boldsymbol{M}$ and $\boldsymbol{N}$. Recall that the asymptotic distribution of $\sqrt{n}\left(\hat{\boldsymbol{\mu}}_s - \boldsymbol{\mu}_{true}\right)$ also depends on the joint distribution of $\boldsymbol{M}$ and $\boldsymbol{N}$ by Lemma 1. Therefore, there is joint convergence in the distribution of $\hat{\boldsymbol{c}}$ and $\sqrt{n}\left(\hat{\boldsymbol{\mu}}_s - \boldsymbol{\mu}_{true}\right)$, and (51) holds.

A rigorous proof of $\hat{\boldsymbol{c}} \xrightarrow{d} \boldsymbol{c}^*$ should be based on the argmax continuous mapping theorem (e.g., Van der Vaart, and Wellner, 1996, Theorem 3.2.2). Intuitively speaking, since $\hat{\boldsymbol{c}}$ is the minimizer of $\hat{Q}(\boldsymbol{c})$, the distribution of $\hat{\boldsymbol{c}}$ depends on the distribution of $\hat{\boldsymbol{\delta}}$. Hence, the asymptotic distribution of $\hat{\boldsymbol{c}}$ depends on the joint distribution of $\boldsymbol{M}$ and $\boldsymbol{N}$. If the quadratic programming is positive definite, then its minimizer $\hat{\boldsymbol{c}}$ is unique and $\hat{\boldsymbol{c}} = O_{\mathrm{P}}(1)$. As the minimizer, $\hat{Q}(\hat{\boldsymbol{c}}) \leq \inf_c \hat{Q}(\boldsymbol{c}) + o_{\mathrm{P}}(1)$. Hence, the assumption $\hat{\boldsymbol{c}} \xrightarrow{d} \boldsymbol{c}^*$ is likely to hold.

□

Proof of the equivalence of confidence intervals. Equation (49) indicates that

$$\sqrt{n}\left(\hat{\boldsymbol{\mu}}_{full} - \boldsymbol{\mu}_{true}\right) = \frac{\partial \boldsymbol{\mu}_0}{\partial \boldsymbol{\theta}^T} \boldsymbol{J}_{\theta\theta}^{-1} \boldsymbol{M} + \boldsymbol{W}(\boldsymbol{\delta} - \boldsymbol{D}) + O_{\mathrm{P}}\left(n^{-1/2}\right).$$

Hence, the full model confidence interval for μ_i is

$$\left[\hat{\mu}_{i,full} - z_{1-\alpha/2}\frac{\hat{\kappa}_i}{\sqrt{n}}, \qquad \hat{\mu}_{i,full} + z_{1-\alpha/2}\frac{\hat{\kappa}_i}{\sqrt{n}}\right],$$

where $\hat{\mu}_{i,full}$ is the ith entry of $\hat{\boldsymbol{\mu}}_{full}$, $z_{1-\alpha/2}$ is the $1-\alpha/2$ quantile of the standard normal distribution, and κ_i is (i,i)th entry of the covariance matrix of $\frac{\partial \mu_0}{\partial \boldsymbol{\theta}^T}\boldsymbol{J}_{\theta\theta}^{-1}\boldsymbol{M} - \boldsymbol{W}\boldsymbol{D}$ with $\hat{\kappa}_i$ being its estimator. Contrasting it with equation (6) in the main text, it suffices to show that

$$\bar{\boldsymbol{\mu}}(\hat{\boldsymbol{c}}) - \hat{\boldsymbol{W}}\left[\boldsymbol{I} - \left(\sum_s \hat{c}_s \hat{\boldsymbol{K}}^{(s)}\right)\hat{\boldsymbol{K}}^{-1}\right]\hat{\boldsymbol{\delta}}/\sqrt{n} \;=\; \hat{\boldsymbol{\mu}}_{full} + o_{\mathrm{P}}\left(n^{-1/2}\right). \tag{52}$$

Since $\hat{\boldsymbol{W}}$, $\hat{\boldsymbol{K}}$ and $\hat{\boldsymbol{K}}^{(s)}$ are consistent estimators of $\boldsymbol{W}$, $\boldsymbol{K}$ and $\boldsymbol{K}^{(s)}$ respectively, we get $\hat{\boldsymbol{W}} = \boldsymbol{W} + o_{\mathrm{P}}(1)$ and $\hat{\boldsymbol{K}}^{(s)}\hat{\boldsymbol{K}}^{-1} = \boldsymbol{K}^{(s)}\boldsymbol{K}^{-1} + o_{\mathrm{P}}(1)$. Since the entries of $\hat{\boldsymbol{c}}$ are bounded between 0 and 1, $\hat{\boldsymbol{c}}o_{\mathrm{P}}(1) = o_{\mathrm{P}}(1)$. Hence,

$$\begin{aligned}
\hat{\boldsymbol{W}}\left[\boldsymbol{I} - \left(\sum_s \hat{c}_s \hat{\boldsymbol{K}}^{(s)}\hat{\boldsymbol{K}}^{-1}\right)\right]\hat{\boldsymbol{\delta}}/\sqrt{n} &= (\boldsymbol{W} + o_{\mathrm{P}}(1))\left[\boldsymbol{I} - \sum_s \hat{c}_s \boldsymbol{K}^{(s)}\boldsymbol{K}^{-1} + \sum_s \hat{c}_s o_{\mathrm{P}}(1)\right]\hat{\boldsymbol{\delta}}/\sqrt{n} \\
&= \boldsymbol{W}\left[\boldsymbol{I} - \sum_s \hat{c}_s \boldsymbol{K}^{(s)}\boldsymbol{K}^{-1}\right]\hat{\boldsymbol{\delta}}/\sqrt{n} + o_{\mathrm{P}}\left(n^{-1/2}\right), \qquad (53)
\end{aligned}$$

where the last equality holds since $\hat{\boldsymbol{\delta}} \xrightarrow{d} \boldsymbol{D}$ and $o_{\mathrm{P}}(1)\,\hat{\boldsymbol{\delta}} \xrightarrow{p} 0$. In the likelihood context, Wang and Zhou (2013) showed that

$$\bar{\boldsymbol{\mu}}(\hat{\boldsymbol{c}}) - \boldsymbol{W}\left[\boldsymbol{I} - \left(\sum_s \hat{c}_s \boldsymbol{K}^{(s)}\right)\boldsymbol{K}^{-1}\right]\hat{\boldsymbol{\delta}}/\sqrt{n} \;=\; \hat{\boldsymbol{\mu}}_{full} + o_{\mathrm{P}}\left(n^{-1/2}\right). \tag{54}$$

Even though they only considered the likelihood context, it turns out that their proof is directly applicable to the context of SEM with ordinal data, which means that (54) also holds in ordinal SEM. Hence, (53) and (54) lead to (52).

□

Theorem 3. *Let the focus parameter be* $\boldsymbol{\mu} = \boldsymbol{\beta}$. *Support that the assumptions in Lemma 1 hold, that the Assumptions* A1 *to* A5 *hold, and that the joint convergence in distribution* (51) *holds. Then,* $T_{FMA} = T_{full} + o_P(1)$, *where* $T_{full} = n\left(\hat{\boldsymbol{\rho}} - \hat{\boldsymbol{\sigma}}_{full}\right)^T \hat{\boldsymbol{V}}\left(\hat{\boldsymbol{\rho}} - \hat{\boldsymbol{\sigma}}_{full}\right)$ *is the test statistic for the full model.*

Proof of Theorem 3. Since both T_{FMA} and T_{full} are quadratic forms, it suffices to show that

$$\sqrt{n}\left(\hat{\boldsymbol{\rho}}-\bar{\boldsymbol{\sigma}}\right)+\frac{\partial\boldsymbol{\sigma}\left(\hat{\boldsymbol{\beta}}_{full}\right)}{\partial\boldsymbol{\beta}^T}\hat{\boldsymbol{W}}\left(\boldsymbol{I}-\sum_s\hat{c}_s\hat{\boldsymbol{K}}^{(s)}\hat{\boldsymbol{K}}^{-1}\right)\hat{\boldsymbol{\delta}} \;=\; \sqrt{n}\left(\hat{\boldsymbol{\rho}}-\boldsymbol{\sigma}\left(\hat{\boldsymbol{\mu}}_{full}\right)\right)+o_{\mathrm{P}}\left(1\right).$$

Because of (53), we get

$$\begin{aligned}
&\frac{\partial\boldsymbol{\sigma}\left(\hat{\boldsymbol{\beta}}_{full}\right)}{\partial\boldsymbol{\beta}^T}\hat{\boldsymbol{W}}\left(\boldsymbol{I}-\sum_s\hat{c}_s\hat{\boldsymbol{K}}^{(s)}\hat{\boldsymbol{K}}^{-1}\right)\hat{\boldsymbol{\delta}}\\
=\;&\left(\frac{\partial\boldsymbol{\sigma}\left(\boldsymbol{\beta}_0\right)}{\partial\boldsymbol{\beta}^T}+o_{\mathrm{P}}\left(1\right)\right)\left[\boldsymbol{W}\left(\boldsymbol{I}-\sum_s\hat{c}_s\boldsymbol{K}^{(s)}\boldsymbol{K}^{-1}\right)\hat{\boldsymbol{\delta}}+o_{\mathrm{P}}\left(1\right)\right]\\
=\;&\frac{\partial\boldsymbol{\sigma}\left(\boldsymbol{\beta}_0\right)}{\partial\boldsymbol{\beta}^T}\boldsymbol{W}\left(\boldsymbol{I}-\sum_s\hat{c}_s\boldsymbol{K}^{(s)}\boldsymbol{K}^{-1}\right)\hat{\boldsymbol{\delta}}+o_{\mathrm{P}}\left(1\right), \qquad (55)
\end{aligned}$$

where the second equality holds since $\hat{\boldsymbol{c}}o_{\mathrm{P}}\left(1\right)=o_{\mathrm{P}}\left(1\right)$, $\hat{\boldsymbol{\delta}}\xrightarrow{d}\boldsymbol{D}$ and $o_{\mathrm{P}}\left(1\right)\hat{\boldsymbol{\delta}}\xrightarrow{p}0$. Note that

$$\bar{\boldsymbol{\sigma}} \;=\; \boldsymbol{\sigma}_0+\frac{\partial\boldsymbol{\sigma}\left(\boldsymbol{\mu}_0\right)}{\partial\boldsymbol{\mu}^T}\left[\bar{\boldsymbol{\mu}}\left(\hat{\boldsymbol{c}}\right)-\boldsymbol{\mu}_0\right]+O_{\mathrm{P}}\left(n^{-1}\right)$$

by (51), and

$$\boldsymbol{\sigma}\left(\hat{\boldsymbol{\mu}}_{full}\right) \;=\; \boldsymbol{\sigma}_0+\frac{\partial\boldsymbol{\sigma}\left(\boldsymbol{\mu}_0\right)}{\partial\boldsymbol{\mu}^T}\left(\hat{\boldsymbol{\mu}}_{full}-\boldsymbol{\mu}_0\right)+O_{\mathrm{P}}\left(n^{-1}\right)$$

by (49). Then

$$\bar{\boldsymbol{\sigma}} \;=\; \boldsymbol{\sigma}\left(\hat{\boldsymbol{\mu}}_{full}\right)+\frac{\partial\boldsymbol{\sigma}\left(\boldsymbol{\mu}_0\right)}{\partial\boldsymbol{\mu}^T}\left[\bar{\boldsymbol{\mu}}\left(\hat{\boldsymbol{c}}\right)-\hat{\boldsymbol{\mu}}_{full}\right]+O_{\mathrm{P}}\left(n^{-1}\right). \qquad (56)$$

Hence, using (55), (56), and (54), we further obtain

$$\begin{aligned}
&\sqrt{n}\left(\hat{\boldsymbol{\rho}}-\bar{\boldsymbol{\sigma}}\right)+\frac{\partial\boldsymbol{\sigma}\left(\hat{\beta}_{full}\right)}{\partial\boldsymbol{\beta}^T}\hat{\boldsymbol{W}}\left(\boldsymbol{I}-\sum_s\hat{c}_s\hat{\boldsymbol{K}}^{(s)}\hat{\boldsymbol{K}}^{-1}\right)\hat{\boldsymbol{\delta}}\\
=\;&\sqrt{n}\left(\hat{\boldsymbol{\rho}}-\bar{\boldsymbol{\sigma}}\right)+\frac{\partial\boldsymbol{\sigma}\left(\boldsymbol{\beta}_0\right)}{\partial\boldsymbol{\beta}^T}\boldsymbol{W}\left(\boldsymbol{I}-\sum_s\hat{c}_s\boldsymbol{K}^{(s)}\boldsymbol{K}^{-1}\right)\hat{\boldsymbol{\delta}}+o_{\mathrm{P}}\left(1\right)\\
=\;&\sqrt{n}\left(\hat{\boldsymbol{\rho}}-\boldsymbol{\sigma}\left(\hat{\boldsymbol{\mu}}_{full}\right)\right)-\sqrt{n}\frac{\partial\boldsymbol{\sigma}\left(\boldsymbol{\mu}_0\right)}{\partial\boldsymbol{\mu}^T}\left[\bar{\boldsymbol{\mu}}\left(\hat{\boldsymbol{c}}\right)-\hat{\boldsymbol{\mu}}_{full}\right]+\frac{\partial\boldsymbol{\sigma}\left(\boldsymbol{\beta}_0\right)}{\partial\boldsymbol{\beta}^T}\boldsymbol{W}\left(\boldsymbol{I}-\sum_s\hat{c}_s\boldsymbol{K}^{(s)}\boldsymbol{K}^{-1}\right)\hat{\boldsymbol{\delta}}+o_{\mathrm{P}}\left(1\right)\\
=\;&\sqrt{n}\left(\hat{\boldsymbol{\rho}}-\boldsymbol{\sigma}\left(\hat{\boldsymbol{\mu}}_{full}\right)\right)+o_{\mathrm{P}}\left(1\right),
\end{aligned}$$

which completes the proof.

□

4 References

Ferguson, T. S. (1996). *A course in large sample theory.* Chapman & Hall, London, UK.

Gong, G. and Samaniego, F. J. (1981). Pseudo maximum likelihood estimation: theory and applications. *The Annals of Statistics*, 9:861–869.

Jiang, J. (2010). *Large sample techniques for statistics.* Springer, New York, NY.

Jin, S. and Ankargren, S. (2019). Frequentist model averaging in structural equation modeling. *Psychometrika*, 84:84–104.

Jöreskog, K. G. (1994). On the estimation of polychoric correlations and their asymptotic covariance matrix. *Psychometrika*, 59:381–389.

Kamat, A. R. (1953). Incomplete and absolute moments of the multivariate normal distribution with some applications. *Biometrika*, 40:20–34.

Liu, C.-A. (2015). Distribution theory of the least squares averaging estimator. *Journal of Econometrics*, 186:142–159.

Olsson, U. (1979). Maximum likelihood estimation of the polychoric correlation coefficient. *Psychometrika*, 44:443–460.

Rhemtulla, M., Brosseau-Liard, P. E., and Savalei, V. (2012). When can categorical variables be treated as continuous? A comparison of robust continuous and categorical sem estimation methods under suboptimal conditions. *Psychological Methods*, 17:354–373.

Richmond, T. (2020). *General topology.* De Gruyter, Berlin, Germany.

Rosseel, Y. (2012). lavaan: An R package for structural equation modeling. *Journal of Statistical Software*, 48:1–36.

Rudin, W. (1976). *Principles of Mathematical Analysis*. McGraw-Hill, New York, NY, 3rd edition.

Van der Vaart,, A. and Wellner, J. (1996). *Weak Convergence and Empirical Processes*. Springer-Verlag, New York, NY.

van der Vaart, A. W. (1998). *Asymptotic Statistics*. Cambridge University Press, Cambridge, UK.

Wang, H. and Zhou, S. Z. F. (2013). Interval estimation by frequentist model averaging. *Communications in Statistics: Theory and Methods*, 42:4342–4356.
